# Solid-Phase Synthesis of Boranophosphate/Phosphorothioate/Phosphate Chimeric Oligonucleotides and Their Potential as Antisense Oligonucleotides

**DOI:** 10.1021/acs.joc.1c01812

**Published:** 2021-12-15

**Authors:** Yuhei Takahashi, Kazuki Sato, Takeshi Wada

**Affiliations:** Department of Medicinal and Life Science, Faculty of Pharmaceutical Sciences, Tokyo University of Science, 2641 Yamazaki, Noda, Chiba 278-8510, Japan

## Abstract

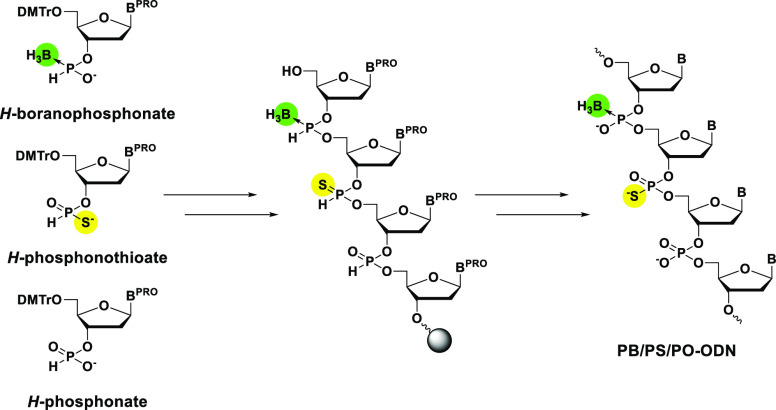

In this study, we successfully synthesized boranophosphate (PB), phosphorothioate (PS), and phosphate (PO) chimeric oligonucleotides (ODNs) as a candidate for the antisense oligonucleotides (ASOs). The PB/PS/PO-ODNs were synthesized utilizing *H*-boranophosphonate, *H*-phosphonothioate, and *H*-phosphonate monomers. Each monomer was condensed with a hydroxy group to create *H*-boranophosphonate, *H*-phosphonothioate, and *H*-phosphonate diester linkages, which were oxidized into PB, PS, and PO linkages in the final stage of the synthesis, respectively. As for condensation of an *H*-phosphonothioate monomer, regulating chemoselectivity was necessary since the monomer has two nucleophilic centers: S and O atoms. To deal with this problem, we used phosphonium-type condensing reagents, which could control the chemoselectivity. In this strategy, we could synthesize PB/PS/PO oligomers, including a 2′-OMe gapmer-type dodecamer. The physiological and biological properties of the synthesized chimeric ODNs were also evaluated. Insights from the evaluation of physiological and biological properties suggested that the introduction of suitable *P*-modification and sugar modification at proper sites of ODNs would control the duplex stability, nuclease resistance, RNase H-inducing ability, and one base mismatch discrimination ability, which are critical properties as potent ASOs.

## Introduction

Many efforts have been devoted to the development of antisense oligonucleotides (ASOs) since it is demonstrated that an oligonucleotide that is complementary to a target mRNA could control the translation of mRNA into a protein.^1,2^ There are two kinds of ASOs according to a mechanism of translation regulation, namely, a steric blocking type and an RNase H*-*dependent type. RNase H is an endonuclease located mainly in the nucleus, recognizes DNA/RNA duplexes, and selectively cleaves the RNA strand.^3,4^ Required properties for potent RNase H-dependent ASO include high nuclease resistance, duplex stability, RNase H-inducing ability, high retention in tissues, and low cytotoxicity. Since the properties of ASOs can be modulated by introducing chemical modifications to phosphate (PO) moieties, sugar, and the nucleobase of nucleotides,^5^ many groups have investigated a wide variety of modifications. A phosphorothioate (PS) backbone, in which one of the nonbridging oxygen atoms is replaced with a sulfur atom, is the most used chemical modification applied to ASOs. PS is still widely used as a chemically modified analogue owing to its high nuclease resistance and the facility of its synthesis. In addition to this, it has been demonstrated that PS linkages are crucial for improving the pharmacokinetics of ASOs due to their high affinity with certain kinds of protein such as serum albumin.^6,7^ For example, it has been found that PS linkages prevent ASOs from glomerular filtration clearance, which led to the extension of blood retention. In addition to this, ASOs containing PS linkages bind specific proteins outside the cell, which leads to the uptake of ASOs into cells,^8^ and intracellular proteins determining the intracellular distribution of ASOs.^9^ However, it has been reported that PS linkages reduce duplex stabilities of ASOs with target mRNAs. Moreover, some PS oligonucleotides (ODNs) are cytotoxic and trigger undesired immunoresponse events, which are major obstacles for clinical trials.^10,11^

To improve duplex stability, nucleotides with sugar modifications have been introduced, such as 2′-*O*-modifications including 2′-OMe and 2′-*O*-MOE,^12^ and locked nucleic acids (LNAs).^13−15^ These modifications are typically used for gapmer-type ASOs, namely, the nucleotides with a sugar modification are placed in the 5′- and 3′-end (wing) regions of an ASO to acquire a duplex stability with a complementary RNA while deoxyribonucleotides are located in the central (gap) region to maintain an RNase H-inducing ability^16^ Some ASOs that were approved by the Food and Drug Administration (FDA) contain both PS linkages and sugar modifications.^17^ This suggests that a combination of *P*-modifications and sugar modifications dramatically improves their properties as ASOs. Alternatively, although some reports suggested that 2′-*O*-MOE gapmers containing PS linkages lower their cytotoxicity,^18^ further suppression of cytotoxicity would be needed for the more secured ASOs.

Under these circumstances, boranophosphate (PB), in which a nonbridging oxygen atom of a PO linkage is replaced with a borano group, has received a lot of attention as another promising ASO candidate since PB modification offers higher nuclease resistance than the PS counterpart^19^ and exhibits low cytotoxicity.^20,21^ However, a full PB modification reduces the duplex stability of ASOs with target mRNAs and RNase H-inducing activities.^19,22−24^

To overcome these problems, Caruthers et al.^24−26^ and our group^27^ introduced both PB and PO linkages in ODNs (PB/PO chimeric ODNs). PB/PO chimeric ODNs have improved duplex stability and some of them show higher RNase H*-*inducing activity compared to the fully PB-modified counterparts. Notably, PB/PO chimeric ODNs have substantial nuclease resistance.^25^ These indicate that *P*-modified chimeric ODNs can take advantage of each other’s strength. This strategy utilizing chimeric ODNs also works for ODNs containing PS linkages. The research from IONIS Pharmaceuticals, Inc. revealed that replacing a few PS linkages with alkylphosphonate linkages significantly reduces toxicity while maintaining the antisense activity of ASOs.^28^ Furthermore, replacing a few PS linkage with mesylphosphoramidate^29^ linkages can also suppress toxicity and in certain cases improve the antisense activity of ASOs.^30^ Taking that into consideration, we expected that introducing PB and PO linkages to PS-ODNs (PB/PS/PO and/or PB/PS chimeric ODNs) would be a promising way to modulate cytotoxicity along with maintaining the favorable pharmacokinetics of ODNs.

Although PB derivatives have been viewed as promising ASO candidates, examples for their synthesis are still limited. The biggest hurdle for the synthesis of PB derivatives is the fact that acyl-type amino protecting groups on nucleobases are not compatible with the synthesis of boranophosphate by the general phosphoramidite method since *N*-acyl groups were easily reduced with a boronating reagent to *N*-alkyl groups^31^ which cannot be removed. To deal with this problem, Caruthers et al. and our group obtained PB derivatives in different synthetic strategies. Caruthers et al. have synthesized PB-ODNs via the phosphoramidite method using *N*-di-*tert*-butylisobutylsilyl (*N*-BIBS)-protected phosphoramidite monomers. This protecting group is stable under the general reaction conditions of the phosphoramidite method^26^ and tolerant toward boronation. In contrast, we have developed the *H*-boranophosphonate method using an *H-*boranophosphonate monoester, which contains characteristic H–P → BH_3_ groups as monomer units.^32,33^ In this method, an *H-*boranophosphonate monomer unit is condensed with a 5′-hydroxy group using a condensing reagent to form an *H*-boranophosphonate diester linkage followed by detritylation step without a transformation of the resultant internucleotidic linkages. These two steps are repeated and after the designed length is achieved, all internucleotidic *H-*boranophosphonate diesters are oxidized to PB linkages by treatment with CCl_4_ and water in the presence of a base, followed by the removal of amino protecting groups and release from a solid support. Caruthers’s and our synthetic strategies were also applicable to the synthesis of PB/PO chimeric ODNs. We have synthesized PB/PO chimeric ODNs using *H-*boranophosphonate monomers and *H*-phosphonate monomers.^27^ Briefly, we utilized *H-*boranophosphonate monomers and *H*-phosphonate monomers to the chain elongation reactions with a condensing reagent to give *H-*boranophosphonate and *H*-phosphonate diester linkages, and these synthetic intermediates were simultaneously oxidized by treatment with CCl_4_ in the presence of water to PB and PO linkages, respectively. Alternatively, to the best of our knowledge, the synthesis of PB/PS/PO chimeric ODNs has never been reported.

**Scheme 1:**
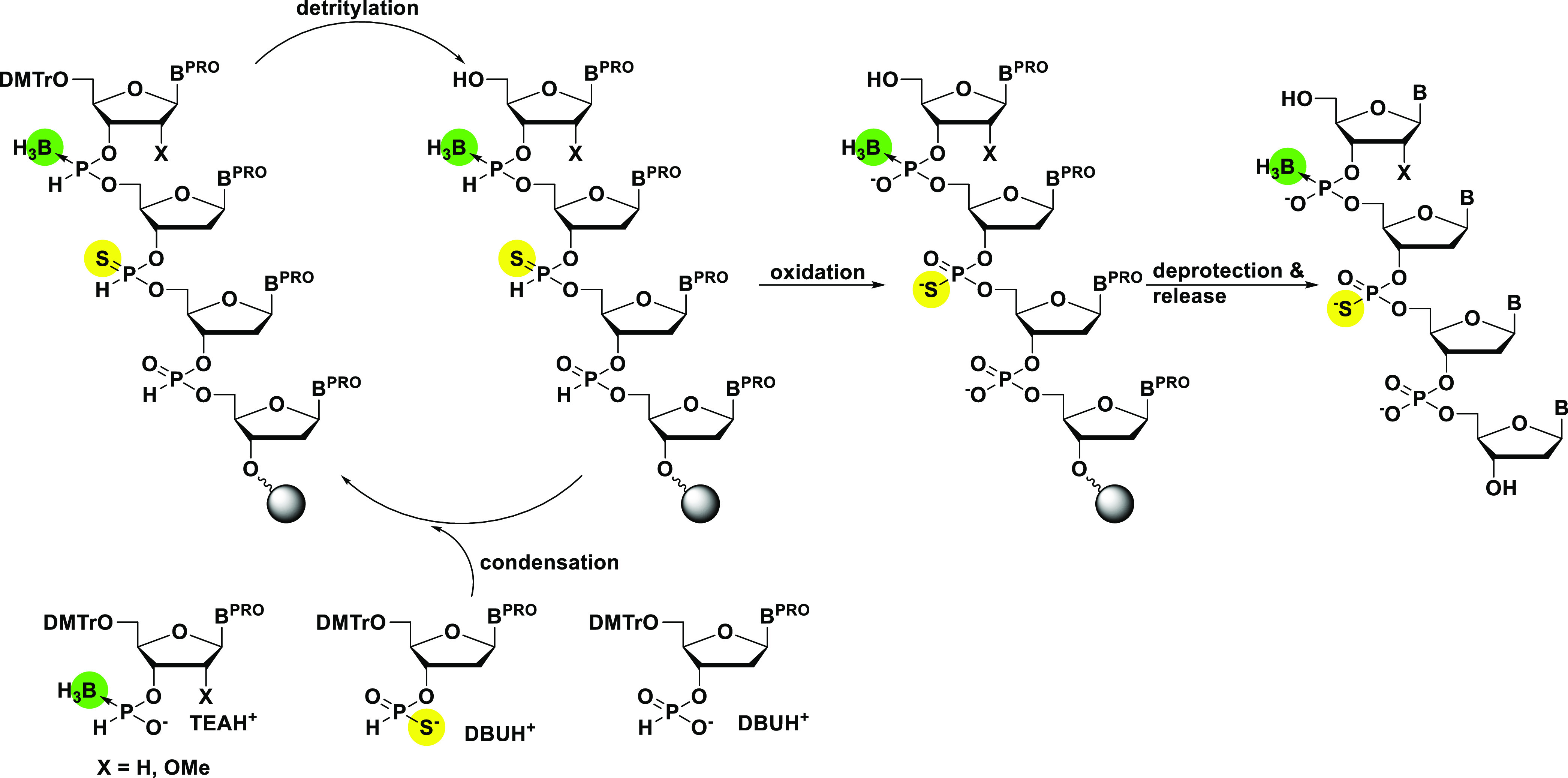
Strategy for the Synthesis of PB/PS/PO Chimeric ODNs in This Study

In this research, we introduced *H*-phosphonothioate monomers into our previous synthetic strategy for PB/PO chimeric ODNs to synthesize PB/PS/PO chimeric ODNs (Scheme 1). This method also allows the synthesis of PB/PS chimeric ODNs. *H*-Phosphonothioate intermediates were synthesized by Stawinski et al. for the first time^34,35^ as the thio analogues of *H*-phosphonate. They condensed the *H*-phosphonothioate monoesters with 5′-hydroxy groups of thymidine derivatives and then oxidized the resultant *H*-phosphonothioate internucleotidic linkages to synthesize sulfur-containing nucleotide analogues such as a PS and a phosphorodithioate.^34,36^ From these insights, we expected that an introduction of PS linkages would be possible by utilizing *H*-phosphonothioate monoesters and the proper oxidation conditions. The major problem associated with using *H*-phosphonothioate monoesters is the chemoselectivity of a condensation reaction since *H*-phosphonothioate monoesters have two different nucleophilic centers, namely, sulfur and oxygen atoms. Desired PS derivatives are obtained by the *O*-activation followed by oxidation while the *S*-activation results in the formation of PO derivatives as byproducts. Stawinski et al. solved the problem using a chlorophosphate derivative, as a condensing reagent since soft *S*-nucleophiles have low reactivity toward the hard phosphorus centers.^36^ However, there are few reports using an *H*-phosphonothioate monomer for solid-phase synthesis.^37−39^ Hence, we tried to synthesize *H*-PS internucleotidic linkages on a solid support using phosphonium-type condensing reagents that is expected to avoid the *S*-activation since these have a hard character. In this research, we demonstrated that this synthetic strategy was applicable to the synthesis of PB/PS/PO chimeric ODNs including gapmer-type ODNs, which have 2′-OMe-modified nucleotides in the wing region.

## Results and Discussion

### Synthesis of Monomer Units

**Scheme 2:**
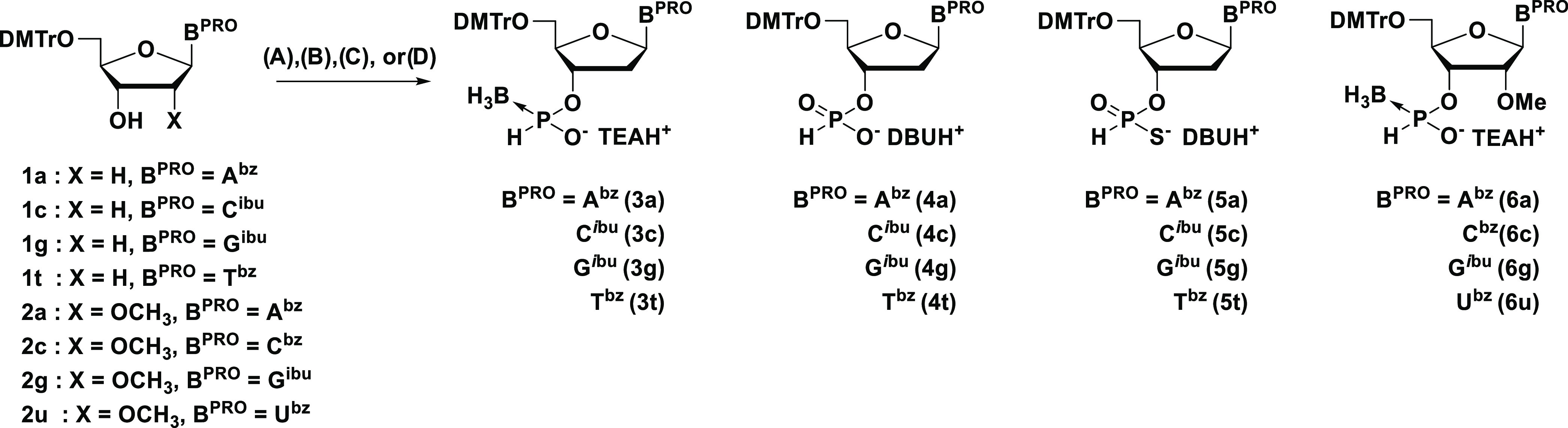
Synthesis of the Monomers Used in This Study Method A (i) **1a**, **1c**, **1g**, or **1t** (1.0 equiv), pyridinium *H-*boranophosphonate (2.0 equiv), bis(2-oxo-3-oxazolidinyl) phosphinic chloride (2.0 equiv), pyridine, 0 °C to room temperature (rt), 1 h (ii) 1.0 M triethylammonium bicarbonate (TEAB) buffer; Method B (i) **1a**, **1c**, **1g**, or **1t** (1.0 equiv), diphenyl *H*-phosphonate (7.0 equiv), pyridine, 0 °C to rt, (ii) H_2_O–TEA (1:1, v/v), rt, (iii) 0.2 M DBU hydrogen carbonate aqueous solution; Method C (i) **1a**, **1c**, **1g**, or **1t** (1.5 equiv), triethylammonium phosphinate (1.0 equiv), pivaloyl chloride (1.5 equiv), 0 °C to rt, 15 min, (ii) elemental sulfur (1.5 equiv), rt, 1 h, (iii) 0.2 M DBU hydrogen carbonate aqueous solution; Method D (i) **2a**, **2c**, **2g**, or **2u** (1.0 equiv), pyridinium *H-*boranophosphonate (2.0 equiv), bis(2-oxo-3-oxazolidinyl) phosphinic chloride (2.0 equiv), pyridine, 0 °C to rt, 1 h, (ii) 1.0 M TEAB buffer.

Deoxyribonucleoside 3′-*H-*boranophosphonate monomers **3a**, **3c**, **3g**, and **3t** were synthesized by following our previous report from the nucleosides **1a**, **1c**, **1g**, and **1t**.^27,33^ An *O*^6^-unprotected deoxyguanosine derivative **3g** was chosen as a monomer unit since it has been shown that **3g** did not cause detectable side reactions^27^ (Scheme 2, method A). The 3′-*H*-phosphonate monomers **4a**, **4c**, **4g**, and **4t** were also synthesized according to a procedure in our preceding publication from the nucleosides **1a**, **1c**, **1g**, and **1t**.^27,40^ The monomers **4a**, **4c**, **4g**, and **4t** were isolated as 1,8-diazabicyclo undecanium salts (DBU salts) because DBU salts were reported to be more stable than the commonly used triethylammonium counterparts (TEA salts)^41^ (Scheme 2, method B). The 3′-*H*-phosphonothioate monomers **5a**, **5c**, **5g**, and **5t** were synthesized by following the literature from the nucleosides **1a**, **1c**, **1g**, and **1t**.^35^ Briefly, the nucleosides **1a**, **1c**, **1g**, and **1t** were condensed with triethylammonium phosphinic acid using PivCl as a condensing reagent, followed by oxidation with elemental sulfur. The 3′-*H*-phosphonothioate monomers **5a**, **5c**, **5g**, and **5t** were also isolated as DBU salts because of the above-mentioned reason. (Scheme 2, method C) (Yields; **5a** 95%, **5c** 99%, **5g** 64%, and **5t** 95%). The 2′-*O*-methyl-ribonucleoside 3′-*H-*boranophosphonate monomers **6a**, **6c**, **6g**, and **6u** were synthesized in a similar way to the synthesis of deoxyribonucleoside 3′-*H-*boranophosphonate monomers^27,33^ with good yields (Scheme 2, method D) (Yields: **6a** 95%, **6c** 90%, **6g** 92%, and **6u** 74%).

### Solid-Phase Synthesis of Phosphorothioate Dimers

**Scheme 3:**
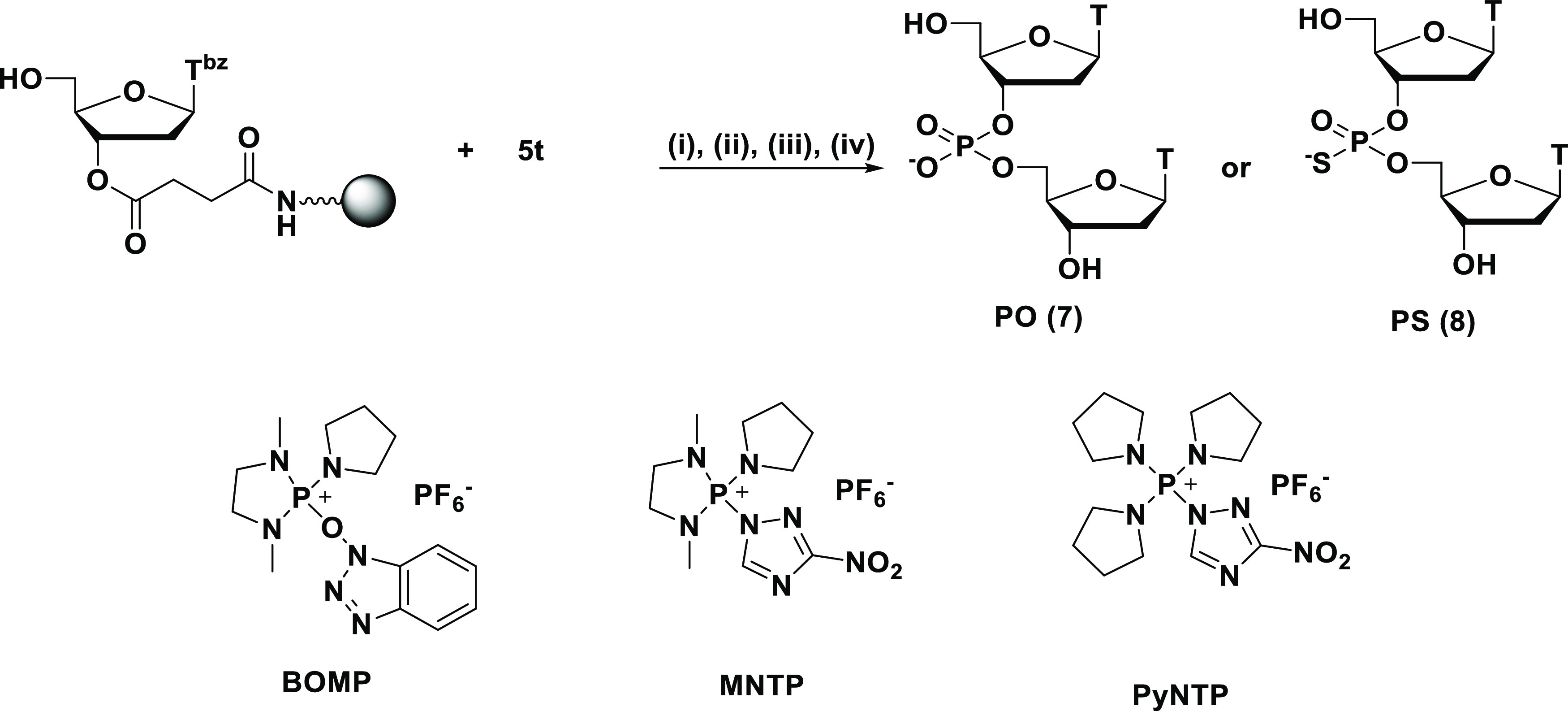
Solid-Phase Synthesis of T_PS_T Dimer^a^ Reagents and conditions: (i) 0.1 M monomer, 0.25 M condensing reagent, 0.6 M base, CH_3_CN, rt, 3 min; (ii) 3% DCA, CH_2_Cl_2_–Et_3_SiH (1:1, v/v), rt, 4 × 15 s; (iii) 0.1 M Et_3_N in CCl_4_–2,6-lutidine–H_2_O (5:12.5:1, v/v/v), rt, 90 min; (iv) concentrated aqueous NH_3_–EtOH (3:1, v/v), 30 °C, 3 h.

Next, we investigated the reaction conditions for solid-phase synthesis using the *H*-phosphonothioate monomer **5t** (Scheme 3). The reaction conditions for the solid-phase synthesis using *H-*boranophosphonate and *H*-phosphonate monomers had already been optimized in our previous report.^27^ First, the *H*-phosphonothioate monomer **5t** (0.1 M) was condensed with the 5′-hydroxy group of thymidine on a highly cross-linked polystyrene (HCP) support^42^ via a succinyl linker using CH_3_CN as a solvent and a phosphonium-type condensing reagent (0.25 M) in the presence of a base, then detritylation was conducted by treatment with 3% dichloroacetic acid (DCA) in CH_2_Cl_2_ in the presence of Et_3_SiH as a trityl cation scavenger.^43^ Afterward, oxidation of the resultant *H*-phosphonothioate linkage was carried out using a mixture of CCl_4_ and H_2_O in the presence of triethylamine as a base and 2,6-lutidine as a cosolvent. Finally, deprotection of the *N*^3^-benzoyl group and cleavage of the linker by treatment with concentrated aqueous NH_3_ and EtOH afforded the PS dimer. The crude mixture was analyzed by reversed-phase high-performance liquid chromatography (RP-HPLC). The chemoselectivity was estimated by the area ratios of the PS diester and the PO diester which were derived from the *S*-activation. In addition to this, the HPLC yield was estimated by the area ratios of the PS diester (**8**) to unreacted thymidine and the PO diester (**7**).

First, the effect of condensing reagents on the reaction was investigated (Table 1, entries 1–3). In these experiments, 2,6-lutidine was used as a base in the condensation reaction. When the *H*-phosphonothioate monomer was condensed by 2-(benzotriazol-1-yloxy)-1,3-dimethyl-2-pyrrolidin-1-yl-1,3,2-diazaphospholidinium hexafluorophosphate (BOMP)^44^ which has HOBt as a leaving group, only a trace amount of the PS and the PO diester was obtained (entry 1). On the other hand, the use of condensing reagents such as 1,3-dimethyl-2-(3-nitro-1,2,4-triazol-1-yl)-2-pyrrolidin-1-yl-1,3,2-diazaphospholidinium hexafluorophosphate (MNTP)^45^ (entry 2) and 3-nitro1,2,4-triazol-1-yl-tris(pyrrolidin-1-yl) phosphonium hexafluorophosphate (PyNTP)^45^ (entry 3) which have 3-nitro 1,2,4-triazole (NT) as a leaving group afforded PS diester with over 90% HPLC yields. These results suggested that the presence of NT was critical for the condensation of the *H*-phosphonothioate monomer and the 5′-hydroxy group. Compared with MNTP, PyNTP gave better chemoselectivity and condensation efficiency. Therefore, PyNTP was chosen as a condensing reagent for *H*-phosphonothioate monomers in the following investigations.

**Table 1:** Solid-Phase Synthesis of T_PS_T Dimer

	condensation conditions			
entry	condensing reagents	base (0.6 M)	chemoselectivity (PO:PS)^a^	HPLC yield (%)^b^
1	BOMP	2,6-lutidine		<1
2	MNTP	2,6-lutidine	6:94	91
3	PyNTP	2,6-lutidine	4:96	94
4	PyNTP	pyridine	3:97	94
5	PyNTP	quinoline	2:98	94
6	PyNTP	quinoline^c^	3:97	95
7	PyNTP		1:99	97

a Determined by RP-HPLC: area ratio of T_PS_T and T_PO_T.

b Determined by RP-HPLC: area ratio of T_PS_T/(T_PS_T + T_PO_T + T).

c 1.8 M quinoline was used.

**Scheme 4:**
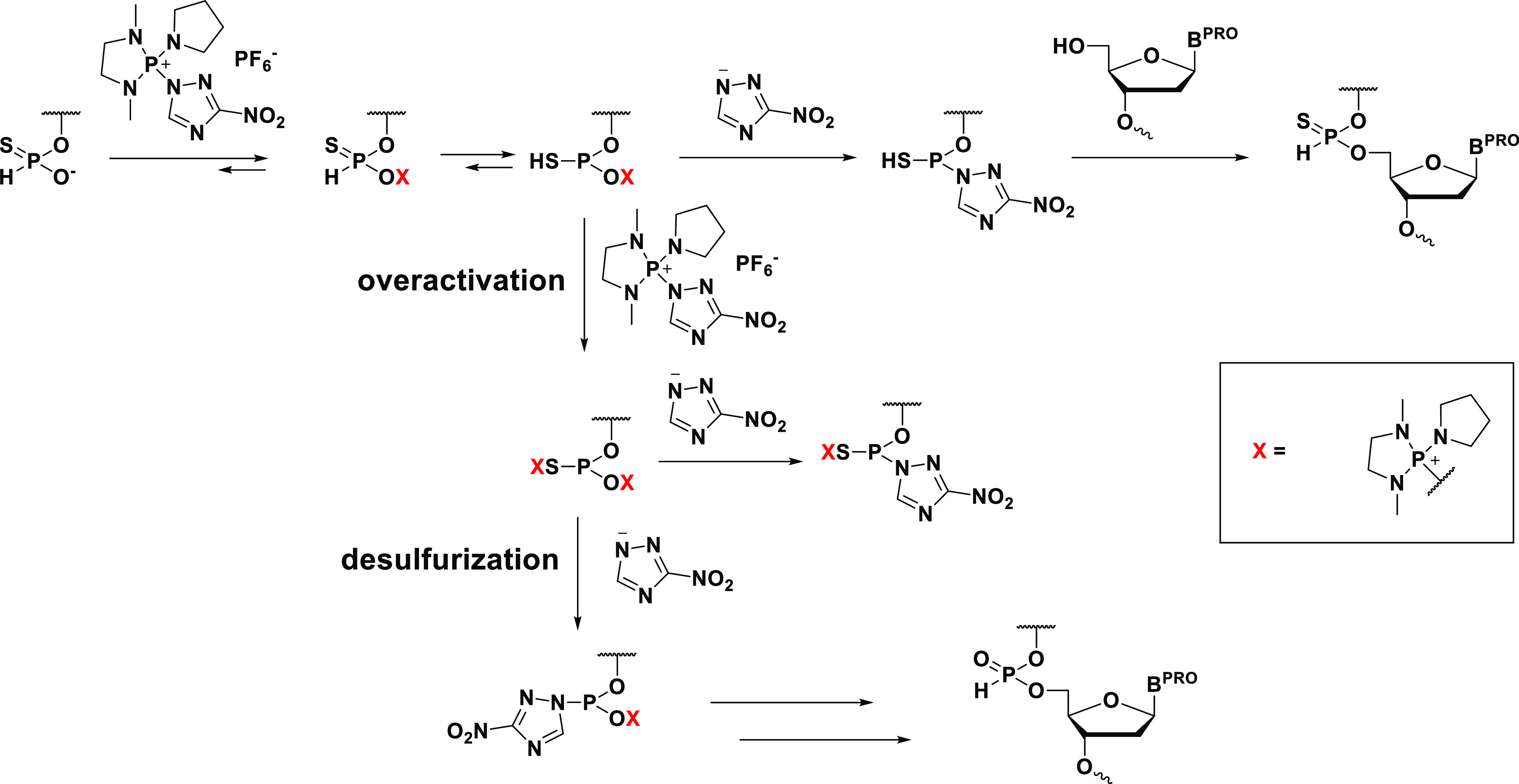
Plausible Mechanism for Overactivation and Formation of T_PO_T

Next, bases for the condensation reaction were examined (entries 3–7). It was found that the use of the weaker base (pyridine, entry 4, quinoline, entry 5) slightly improved chemoselectivities while maintaining high condensation efficiency. Raising the ratio of quinoline in the reaction solvent had a marginal effect on the reaction outcome (entry 6). Surprisingly, the condensation reaction in the absence of base improved both coupling yield and chemoselectivity (entry 7). Therefore, we concluded that the utilization of PyNTP in the presence of 1.8 M quinoline or without a base was the optimum conditions for the condensation reaction. It has been shown that MNTP had a higher activity as a condensing reagent for phosphorylation and phosphonylation reactions than PyNTP,^45^ likely due to less steric hindrance and the strained cyclic structure of the phosphonium center. However, for the condensation reaction using the *H*-phosphonothioate **5t**, PyNTP gave better chemoselectivity and condensation efficiency. In addition, the use of a stronger base for the condensation reaction was found to cause lower chemoselectivity and condensation efficiency. From these observations, an overactivation of the monomer **5t** seemed to affect the reaction outcome. The activation of **5t** by MNTP might be prone to lead the overactivation as shown in Scheme 4 and resulted in inferior chemoselectivity and condensation efficiency. High basicity of the reaction medium may also cause the overactivation of the monomer.

**Scheme 5:**
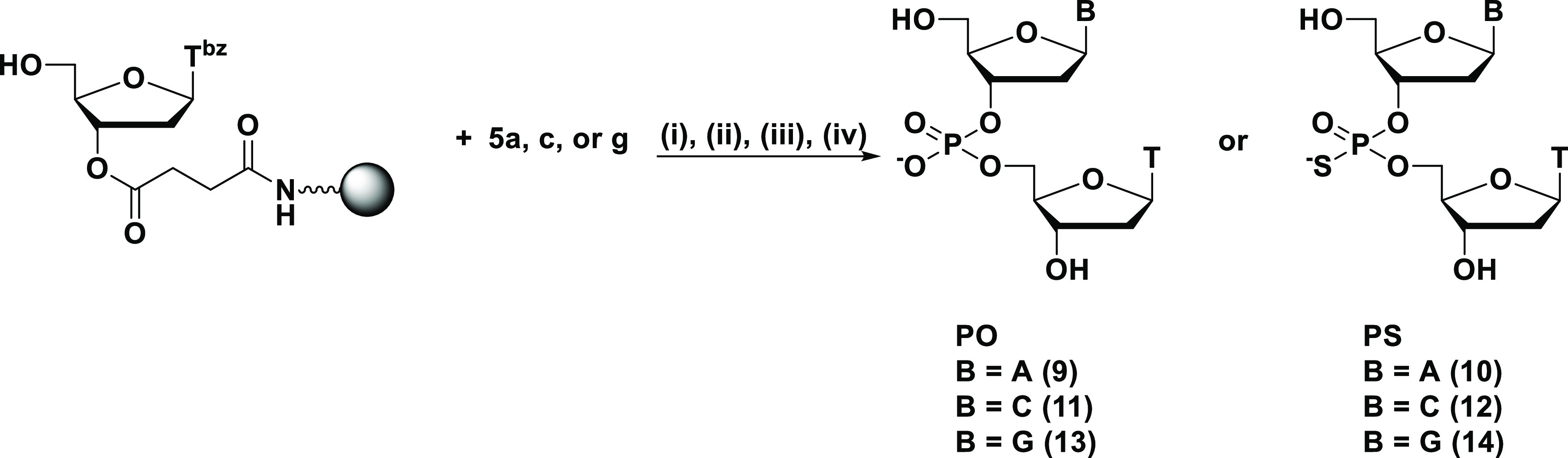
Solid-Phase Synthesis of N_PS_T Dimers Reagents and conditions: (i) 0.1 M monomer, 0.25 M condensing reagent in the presence of 1.8 M base or in the absence of base, CH_3_CN, rt, 3 min; (ii) 3% DCA, CH_2_Cl_2_–Et_3_SiH (1:1, v/v), rt, 4 × 15 s; (iii) 0.1 M Et_3_N in CCl_4_–2,6-lutidine–H_2_O (5:12.5:1, v/v/v), rt, 90 min; (iv) concentrated aqueous NH_3_–EtOH (3:1, v/v), 30 °C, 12 h for entries 1–4, 50 °C, 12 h for entries 5–7.

The optimized reaction conditions were then applied to monomers containing other nucleobases (Scheme 5 and Table 2). The deoxyadenosine **5a**, deoxycytidine **5c**, and deoxyguanosine **5g***H*-phosphonothioate monomers were used to synthesize the dimers **10**, **12**, and **14** following the procedures in entries 6 and 7 of Table 1. In contrast to the thymidine *H*-phosphonothioate monomer, the presence of quinoline as a base was critical for efficient condensation reactions of deoxyadenosine and deoxycytosine counterparts (Table 2, entries 1 vs 2, entries 3 vs 4, respectively). As for the deoxyguanosine *H*-phosphonothioate monomer **5g**, the coupling yield and chemoselectivity were better in the absence of a base, but still not satisfactory (Table 2, entries 5 vs 6). Doubling the equivalents of the deoxyguanosine *H*-phosphonothioate monomer and PyNTP afforded the product in 93% HPLC yield (entry 7). Thus, conditions in entries 1, 3, and 7 were chosen as the optimum conditions for each *H*-phosphonothioate monomer.

**Table 2:** Solid-Phase Synthesis of N_PS_T Dimers

	condensation reaction conditions				
entry	monomer	base	product	chemoselectivity (PO:PS)^a^	HPLC yield (%)^b^
1	**5a**	quinoline	dA_PS_T (**10**)	2:98	95
2	**5a**		dA_PS_T (**10**)	3:97	70
3	**5c**	quinoline	dC_PS_T (**12**)	2:98	96
4	**5c**		dC_PS_T (**12**)	2:98	94
5	**5g**	quinoline	dG_PS_T (**14**)	1:>99	88
6	**5g**		dG_PS_T (**14**)	1:>99	91
7^c^	**5g**		dG_PS_T (**14**)	1:>99	93

a Determined by RP-HPLC: area ratio of N_PS_T and N_PO_T.

b Determined by RP-HPLC: area ratio of N_PS_T/(N_PS_T + N_PO_T + T).

c 0.2 M monomer and 0.5 M condensing reagent were used.

**Scheme 6:**
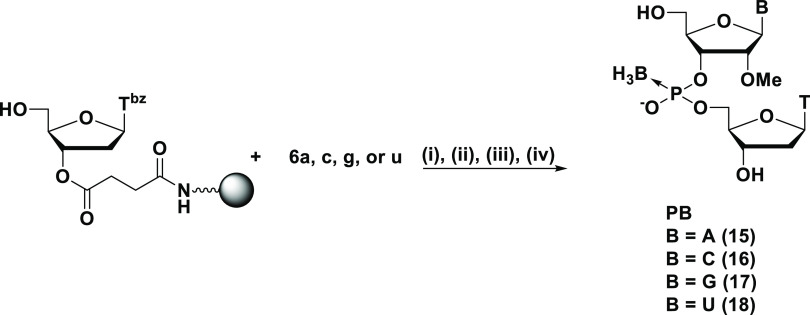
Solid-Phase Synthesis of N_PB_T Dimers Using 2′-OMe-3′-*H-*Boranophosphonate Monomers Reagents and conditions: (i) 0.1 M monomer, 0.25 M MNTP, 0.6 M 2,6-lutidine, CH_3_CN, rt, 3 min; (ii) 3% DCA, CH_2_Cl_2_–Et_3_SiH (1:1, v/v), rt, 4 × 15 s; (iii) 0.1 M Et_3_N in CCl_4_–2,6-lutidine–H_2_O (5:12.5:1, v/v/v), rt, 90 min; (iv) concentrated aqueous NH_3_–EtOH (3:1, v/v), 30 °C, 12 h for entries 1–2, 50 °C, 12 h for entry 3, 30 °C, 3 h for entry 4.

In addition to this, we carried out the solid-phase synthesis of PB dimers using 2′-*O*-methyl-3′-*H-*boranophosphonate monomers (**6a**, **6c**, **6g**, and **6u**) for the synthesis of gapmer-type PB/PS/PO chimeric oligonucleotides. 2′-*O*-Methyl-3′-*H-*boranophosphonate monomers were condensed with the 5′-hydroxy group of thymidine on an HCP support via a succinyl linker under the same conditions with 2′-deoxynucleoside counterparts using MNTP as a condensing reagent in the presence of 2,6-lutidine (Scheme 6). The following procedures were the same as in the synthesis of T_PS_T. Dimers A_PB_T, C_PB_T, G_PB_T, and U_PB_T (underline indicates 2′-OMe nucleoside) were obtained in 95–98% HPLC yields (Table 3), indicating that the condensation reaction proceeded efficiently regardless of the presence of 2′-*O*-modification.

**Table 3:** Solid-Phase Synthesis of N_PB_T Dimers Using 2′-OMe-3′-*H-*Boranophosphonate Monomers

entry	monomer	product^a^	HPLC yield (%)^b^
1	**6a**	A_PB_T (**15**)	95
2	**6c**	C_PB_T (**16**)	96
3	**6g**	G_PB_T (**17**)	98
4	**6u**	U_PB_T (**18**)	97

a Subscript PB = boranophosphate, underline = 2′-OMe modification.

b Determined by RP-HPLC: area ratio of N_PB_T/(N_PB_T + T).

### Solid-Phase Synthesis of PB/PS/PO Chimeric ODNs

Next, to elucidate whether the synthetic strategy is applicable to the synthesis of PB/PS/PO chimeric ODNs, the synthesis of tetramers (d(C_PO_A_PB_G_PS_T) (**19**) and d(C_PS_A_PB_G_PO_T) (**20**)) containing PB, PS, and PO linkages was conducted using the *H*-phosphonothioate, *H*-boranophosphonate, and *H*-phosphonate monomers. The cycle consisting of the condensation and detritylation was repeated, and oxidation of internucleotidic linkages followed by removal of the amino protecting groups and cleavage of the linker afforded the tetramers. It was confirmed that the tetramers were formed as main products by the RP-HPLC analysis of the reaction mixtures (Figure S4). These results indicated that these different internucleotidic linkages were simultaneously oxidized without side reactions, and thus this strategy enables the synthesis of PB/PS/PO chimeric ODNs.

These results prompted us to synthesize a PB/PS/PO chimeric DNA dodecamer, and d(C_PS_A_PS_G_PS_T_PS_C_PB_A_PB_G_PB_T_PB_C_PO_A_PO_G_PO_T) (**21**) was chosen as a synthetic target to demonstrate the potential of the strategy, since **21** contains almost all of the potential combination of internucleotidic linkages and nucleobases. The dodecamer **21** was synthesized by repeating the condensation and detritylation cycles, oxidation, and the deprotection and the release step and was isolated in 6% yield (Table 4, entry 3). Encouraged by the success, we designed and synthesized types of chimeric ODNs containing PB modifications (Table 4, entries 4–6). These sequences were antisense sequences to apoB protein mRNA^46^ and the site of chemical modifications of the PB/PS/PO-ODN **22** was designed as follows: (1) PB or PS linkages were placed at the 3′ and 5′-ends to gain resistance toward exonucleases; (2) four consecutive PO linkages were introduced at the central position of ODNs to improve duplex forming and RNase H-inducing abilities. As for the PB/PS-ODN **23**, PB or PS modifications were introduced at the central position of the sequence instead of PO linkages. Also, the PB/PO-ODN **24** was designed as shown in entry 6. These ODNs were successfully synthesized and isolated in 5–19% yields (Table 4, entries 4–6). Subsequently, we attempted to apply this synthetic strategy to a 2′-OMe gapmer and synthesized G_PB_C_PB_A_PB_ d(T_PS_T_PO_G_PO_G_PO_T_PS_A_PB_)U_PB_U_PB_C (**25**). The RP-HPLC analysis of crude mixture indicated that the desired oligonucleotide was formed as the main product and **25** was isolated in 13% yield (Table 4, entry 7). This result indicated that some 2′-*O*-modified gapmers would be synthesized by this synthetic strategy. It is worth noting that although we had purified PB containing ODNs by anion-exchange HPLC, it was found that RP-HPLC purification using a mixture of methanol and a buffer containing hexafluoroisopropanol and triethylamine as an eluent was effective for isolation of these ODNs.^47^ Thus, all of the ODNs but **22** were purified by RP-HPLC. Although there is room for further improvement of yields, some kinds of chimeric ODNs were successfully synthesized in the strategy.

**Table 4:** Results of the Solid-Phase Synthesis of PB/PS/PO Chimeric ODNs

			isolated yield^b^	*m*/*z*	
entry	abbreviation of ODNs	product^a^	(%)	calcd	found
1		d(C_PS_A_PB_G_PO_T) (**19**)		592.6236	592.6209
2		d(C_PO_A_PB_G_PS_T) (**20**)		592.6236	592.6217
3		d(C_PS_A_PS_G_PS_T_PS_C_PB_A_PB_G_PB_T_PB_C_PO_A_PO_G_PO_T) (**21**)	6	615.7786	615.7767
4	PB/PS/PO	d(G_PB_C_PS_A_PB_T_PO_T_PO_G_PO_G_PO_T_PS_A_PB_T_PS_T_PB_C) (**22**)	19	614.1138	614.1136
5	PB/PS	d(G_PB_C_PS_A_PB_T_PS_T_PS_G_PB_G_PB_T_PS_A_PB_T_PS_T_PB_C) (**23**)	5	618.6194	618.6175
6	PB/PO	d(G_PB_C_PB_A_PB_T_PO_T_PO_G_PO_G_PO_T_PB_A_PB_T_PB_T_PB_C) (**24**)	19	604.9781	604.9774
7	PB/PS/PO-gapmer	G_PB_C_PB_A_PB_ d(T_PS_T_PO_G_PO_G_PO_T_PS_A_PB_)U_PB_U_PB_C (**25**)	13	635.9695	635.9670

a Subscript PB = boranophosphate, PS = phosphorothioate, PO = phosphate, underline = 2′-OMe modification.

b Determined by UV absorbance 260 nm.

### Hybridization Properties

**Figure 1:**
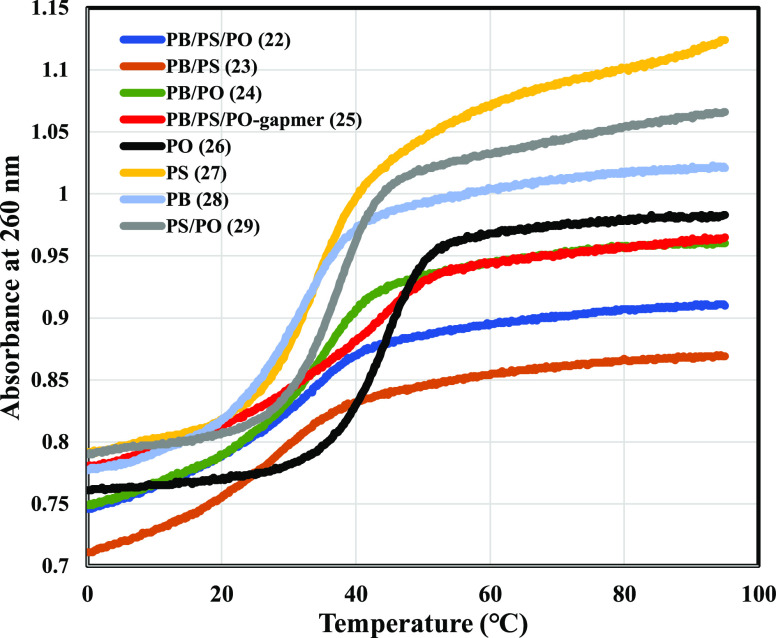
UV Melting Curves of ODNs (**22**–**29**) and cRNA (**30**) Duplexes.

Next, we moved on to the evaluation of properties that are crucial for ASOs. First, hybridization properties of the obtained ODNs were investigated by thermal denaturation tests. The UV melting curves and *T*_m_ values of the ODNs **22–29** with the complementary RNA (cRNA) are shown in Figure 1 and Table 5, respectively. As for PO, PS, and PB-ODNs, the order of hybridization ability was PO-ODN (*T*_m_ value of the duplex with cRNA: 45.0 °C) > PS-ODN (34.0 °C) > PB-ODN (32.2 °C), which was in good agreement with the previous reports.^19,27^ Introduction of PO linkages to PB or PS-ODNs (PB/PO or PS/PO-ODN) improved the duplex stability (PB (*T*_m_ value of the duplex with cRNA: 32.2 °C) vs PB/PO-ODNs (35.0 °C) and PS (34.0 °C) vs PS/PO-ODNs (37.3 °C)). Although PB/PS chimeric ODN reduced its duplex stability compared to the other ODNs, replacing PB or PS linkages with PO linkages (PB/PS/PO chimeric ODN) also improved the duplex stability and the *T*_m_ value was as almost the same as the PS-ODN (PB/PS (*T*_m_ value of the duplex with cRNA: 28.8 °C) vs PB/PS/PO-ODNs (34.0 °C)). In addition, PB/PS/PO chimeric gapmers having 2′-OMe modifications on the wing region showed improved duplex stability (42.3 °C). From these results, it was verified incorporation of sugar modifications and introducing suitable *P*-modifications into proper positions of ASOs could regulate the duplex stability.

**Table 5:** *T*_m_ Values of The ODN/cRNA Duplexes^a^

entry	abbreviation of ODNs	sequence^b^	*T*_m_ (°C)	Δ*T*_m_ (°C)^c^
1	PO	d(G_PO_C_PO_A_PO_T_PO_T_PO_G_PO_G_PO_T_PO_A_PO_T_PO_T_PO_C) (**26**)	45.0	
2	PS	d(G_PS_C_PS_A_PS_T_PS_T_PS_G_PS_G_PS_T_PS_A_PS_T_PS_T_PS_C) (**27**)	34.0	–11.0
3	PB	d(G_PB_C_PB_A_PB_T_PB_T_PB_G_PB_G_PB_T_PB_A_PB_T_PB_T_PB_C) (**28**)	32.2	–12.8
4	PB/PS/PO	d(G_PB_C_PS_A_PB_T_PO_T_PO_G_PO_G_PO_T_PS_A_PB_T_PS_T_PB_C) (**22**)	34.0	–11.0
5	PB/PS	d(G_PB_C_PS_A_PB_T_PS_T_PS_G_PB_G_PB_T_PS_A_PB_T_PS_T_PB_C) (**23**)	28.8	–16.2
6	PB/PO	d(G_PB_C_PB_A_PB_T_PO_T_PO_G_PO_G_PO_T_PB_A_PB_T_PB_T_PB_C) (**24**)	35.0	–10.0
7	PS/PO	d(G_PS_C_PS_A_PS_T_PO_T_PO_G_PO_G_PO_T_PS_A_PS_T_PS_T_PS_C) (**29**)	37.3	–7.7
8	PB/PS/PO-gapmer	G_PB_C_PB_A_PB_ d(T_PS_T_PO_G_PO_G_PO_T_PS_A_PB_)U_PB_U_PB_C (**25**)	42.3	–2.7

a cRNA (**30**): r(G_PO_A_PO_A_PO_U_PO_A_PO_C_PO_C_PO_A_PO_A_PO_U_PO_G_PO_C).

b Subscript PB = boranophosphate, PS = phosphorothioate, PO = phosphate, underline = 2′-OMe modification.

c The difference in *T*_m_ value relative to that of the PO-ODN/cRNA duplex.

### Nuclease Resistance

**Figure 2:**
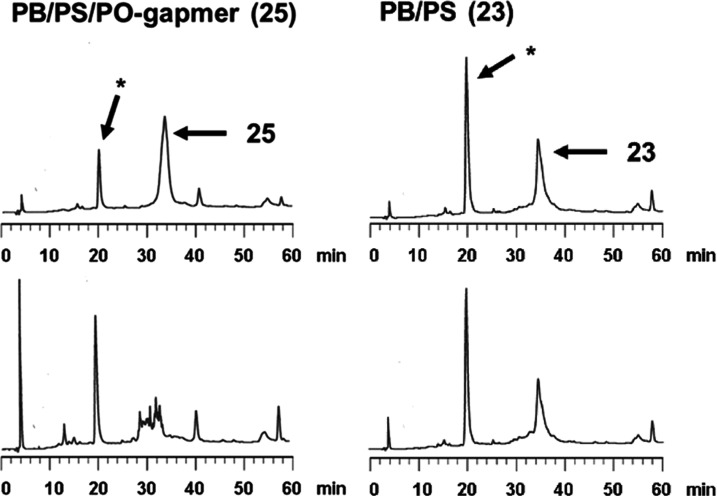
RP-HPLC profiles of ODN before (top) and after (bottom) the treatment with snake venom phosphodiesterase (SVPDE) for 12 h at 37 °C. (* indicates an artifact peak).

Second, nuclease digestion experiments were conducted using snake venom phosphodiesterase (SVPDE) from *Crotalus adamanteus* venom, a representative of 3′-exonuclease. Aqueous solutions of each ODN (**22**–**29**) were treated with SVPDE solution (0.4 U/mL) at 37 °C for 12 h. After SVPDE was denatured at 95 °C, the mixtures were analyzed by RP-HPLC. The results are shown in Figures 2 and S21. The PO-ODN was completely digested while the PS-ODN was partially degraded in the same conditions, respectively (Figure S21). On the other hand, the PB-ODN remained almost intact (Figure S21). Therefore, it was suggested that the order of nuclease resistance toward SVPDE was PB-ODN > PS-ODN > PO-ODN, which was in good agreement with the previous report.^19^ On the other hand, the PB/PO-ODN, PS/PO-ODN, PB/PS/PO-ODN, and PB/PS/PO-gapmer, which have three or four consecutive PO linkages at the central positions, were completely digested and their full-length ODNs were not detected by RP-HPLC (Figures 2 and S21). Although SVPDE is known as a 3′-exonuclease, there are reports that SVPDE also has endonuclease activity.^48−50^ Hence, SVPDE may recognize consecutive PO linkages at the central position as endonuclease. In sharp contrast, there was a slight sign of decomposition of the PB/PS-ODN indicating that combination of PB and PS modifications confers substantial nuclease resistance to ODNs (Figure 2). In the nuclease digestion experiments, it was suggested that the ODNs containing a higher ratio of PO linkages would significantly reduce nuclease resistance.

### RNase H Activity

Finally, we studied the effect of ODNs on RNase H activity, which is crucial for the efficacy of RNase H-dependent antisense therapeutics. *Escherichia coli* RNase H was used for the experiments. Aqueous solutions of each ODN (**22**–**29**) and 10 equiv of cRNA were treated with 25 or 50 U/mL RNase H at 37 °C for 30 min. After RNase H was denatured at 95 °C, the mixtures were analyzed by RP-HPLC. In the assay using 25 U/mL RNase H, although almost all of the ODNs did not show clear effects on RNase H activities, the PO-ODN (**26**) and the PB/PS/PO-gapmer (**25**) were able to induce cleavage of the cRNA strand by RNase H to some extent since small fragments of cRNA were detected by RP-HPLC (Figure S22). Compared with the PO-ODN, the PB/PS/PO-gapmer showed slightly better RNase H activity. In the assay using 50 U/mL RNase H, almost all cRNA was digested by RNase H with all ODNs (Figures 4 and S23). Hence, we calculated the amount of the intact cRNA compared with benzamide as an internal standard based on the area ratio of each peak in HPLC profiles. In the presence of PB/PS/PO-gapmer, PB/PS/PO, PS/PO, PS, and PO-ODNs, over 95% of cRNA was cleaved by RNase H. In contrast, when using PB/PS, PB/PO, and PB-ODN, approximately 90% of cRNA was cleared by RNase H. From these results, it was suggested that increasing the ratio of PB linkages suppressed RNase H activity.

**Figure 3:**
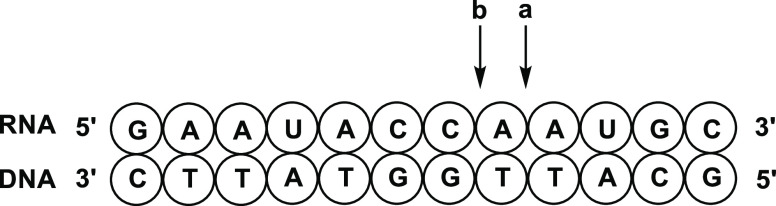
Plausible Cleavage Site of the DNA/RNA Duplex.

**Figure 4:**
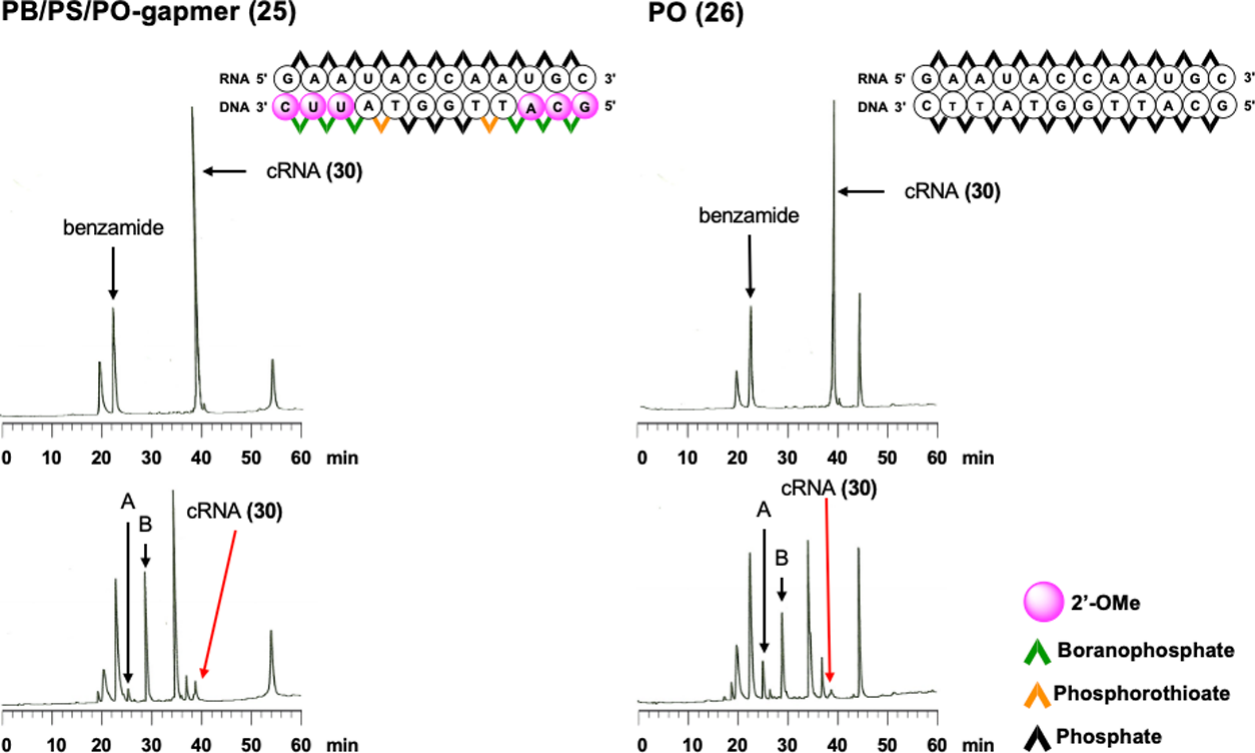
RP-HPLC profiles of ODN/cRNA (**30**) before (top) and after (bottom) the treatment with RNase H for 30 min at 37 °C.

On the other hand, in the RNase H assay, two pairs of cleaved fragments, namely, p4mer and 8mer (cleavage site **a** in Figure 3) and p5mer and 7mer (cleavage site **b** in Figure 3) (p indicates phosphate group at the 5′ end of fragments) were mainly detected by RP-HPLC and electrospray ionization mass spectrometry (ESI-MS) analysis. The peaks derived from the fragments were shown in Figure 3 (fragment **A** was from cleavage of RNA (**30**) at cleavage site **a**; fragment **B** was from cleavage of RNA (**30**) at cleavage site **b**). The area ratios of the peaks **A** and **B** in HPLC profiles were shown in Table 6 and the representative profiles were shown in Figure 4. In the presence of ODNs without PB modification, the area ratios of peaks **A** and **B** were approximately 1:2 (Table 6, entries 1, 2, 7), whereas ODNs containing PB linkages offered varied cleavage site preferences, except for the PB/PS/PO-ODN (**22**). Therefore, it was suggested that ODNs containing PB linkages would change the cleavage site preference. Hence, the effect of PB linkages on the recognition of one base mismatch was studied as follows.

**Table 6:** Ratio of Digested Fragments^a^

			area ratio^b^	
entry	abbreviation of ODNs	sequence^b^	A	B
1	PO	d(G_PO_C_PO_A_PO_T_PO_T_PO_G_PO_G_PO_T_PO_A_PO_T_PO_T_PO_C) (**26**)	1	2.3
2	PS	d(G_PS_C_PS_A_PS_T_PS_T_PS_G_PS_G_PS_T_PS_A_PS_T_PS_T_PS_C) (**27**)	1	2.2
3	PB	d(G_PB_C_PB_A_PB_T_PB_T_PB_G_PB_G_PB_T_PB_A_PB_T_PB_T_PB_C) (**28**)	1	3.5
4	PB/PS/PO	d(G_PB_C_PS_A_PB_T_PO_T_PO_G_PO_G_PO_T_PS_A_PB_T_PS_T_PB_C) (**22**)	1	2.5
5	PB/PS	d(G_PB_C_PS_A_PB_T_PS_T_PS_G_PB_G_PB_T_PS_A_PB_T_PS_T_PB_C) (**23**)	1	>4.5
6	PB/PO	d(G_PB_C_PB_A_PB_T_PO_T_PO_G_PO_G_PO_T_PB_A_PB_T_PB_T_PB_C) (**24**)	1	1.3
7	PS/PO	d(G_PS_C_PS_A_PS_T_PO_T_PO_G_PO_G_PO_T_PS_A_PS_T_PS_T_PS_C) (**29**)	1	2.0
8	PB/PS/PO-gapmer	G_PB_C_PB_A_PB_ d(T_PS_T_PO_G_PO_G_PO_T_PS_A_PB_)U_PB_U_PB_C (**25**)	1	4.0

a Fragment A: r(pAUGC); fragment B: r(pAAUGC).

b Determined by the area ratio of A and B in RP-HPLC profile.

**Figure 5:**
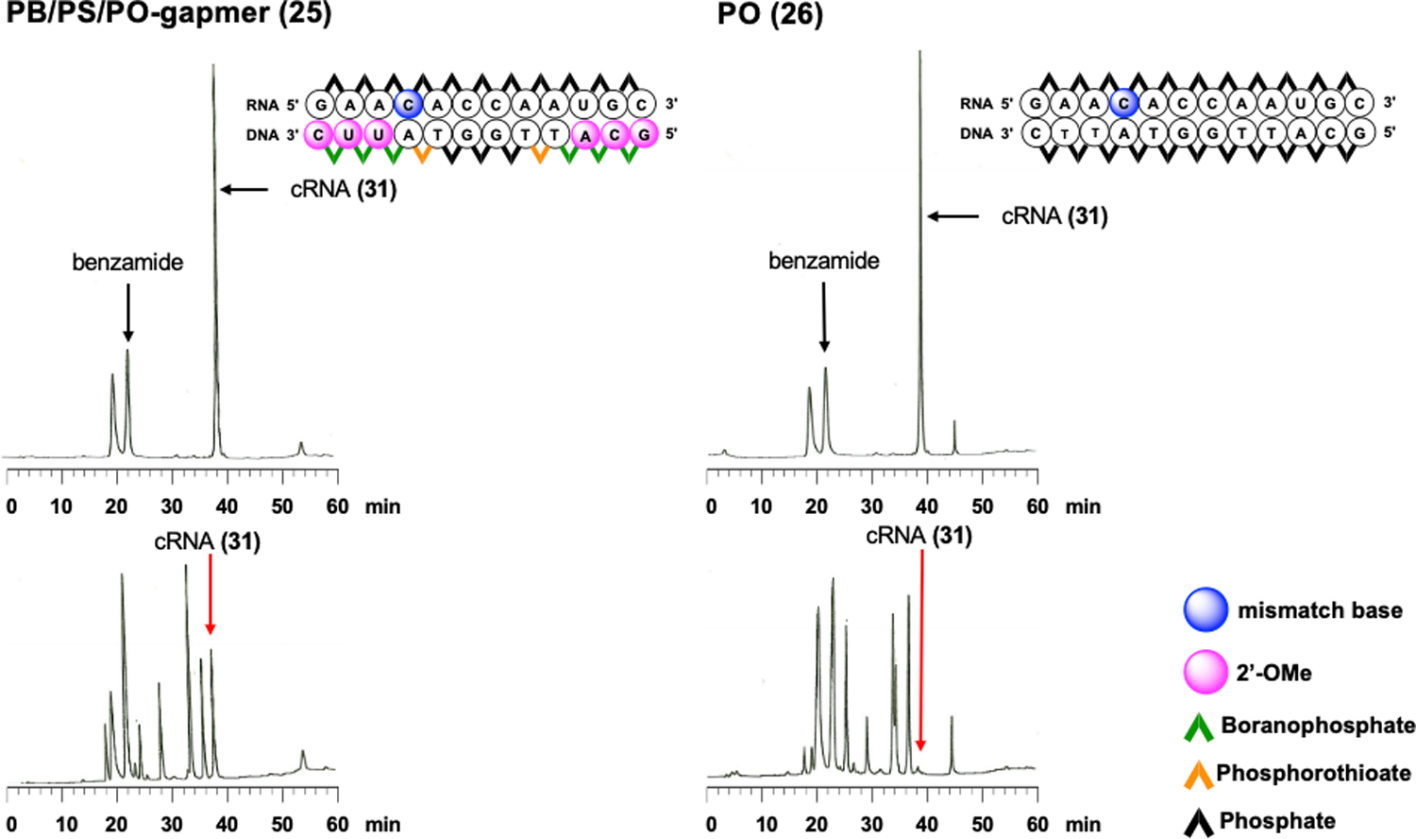
RP-HPLC profiles of ODN/cRNA (**31**) before (top) and after (bottom) the treatment with RNase H for 30 min at 37 °C.

Similar RNase H cleavage experiments were conducted using PB/PS/PO-gapmer (**25**) or PO-ODN (**26**) and the RNA (**31**, r(GAA**C**ACCAAUGC), boldface indicates a mismatch to the ODNs) having one base mismatch to the ODNs. In the presence of PO-ODN, less than 5% of the RNA (**31**) remained, whereas with PB/PS/PO-gapmer, approximately 20% of the RNA (**31**) was intact (Figure 5). To evaluate the duplex stability of PB/PS/PO-gapmer (**25**) and PO-ODN (**26**) with the RNA (**31**), thermal denaturation tests were conducted in a similar way. The UV melting curves of PB/PS/PO-gapmer (**25**) and PO-ODN (**26**) with the RNA (**31**) are shown in Figure S20. The calculated *T*_m_ value of PO-ODN (**26**) and RNA (**31**) duplex was 33.4 °C, 11.6 °C lower than that of the matched duplex, whereas the melting curve of the mixture of the PB/PS/PO-gapmer (**25**) and the RNA (**31**) had no apparent inflection point, indicating the low duplex stability of PB/PS/PO-gapmer (**25**) with the RNA (**31**). Taking these results into consideration, the reduced cleavage of the mismatched RNA strand by RNase H in the presence of the PB/PS/PO-gapmer would be attributed to both the cleavage preference induced by the PB/PS/PO-gapmer and the inferior duplex stability of the PB/PS/PO-gapmer with the complementary RNA with a mismatched base. Thus, although the experiment was a preliminary study, it was demonstrated that proper chemical modification would improve the ability of one base mismatch recognition.

## Conclusions

We developed an efficient synthetic strategy for PB/PS/PO chimeric ODNs without a limitation of the nucleobase and position of *P*-modification utilizing *H*-boranophosphonate, *H*-phosphonothioate, and *H*-phosphonate monomers. In addition to this, we showed that the strategy was applicable to the synthesis of a PB/PS/PO chimeric 2′-OMe gapmer. One of the key points pertaining to the synthesis of these chimeric ODNs was regulating the chemoselectivity of the condensation reaction using an *H*-phosphonothioate monomer. It was found that phosphonium-type condensing reagents having NT as a leaving group, especially PyNTP, provided excellent chemoselectivity. The properties of obtained ODNs were also evaluated. As for a thermal denaturation study, introducing PO linkages to PS-ODN, PB-ODN, and PB/PS-ODN (PS/PO, PB/PO, and PB/PS/PO chimeric ODNs) improved their duplex stability. An SVPDE assay revealed that the introduction of three or four consecutive PO linkages reduced their nuclease resistance significantly. In the RNase H activity assay, it was suggested that ODNs containing a higher ratio of the PB linkages reduced their RNase H activity to some extent, and altered the cleavage site preference of RNase H. From these results, it was expected that introducing proper *P*-modifications at appropriate sites of ASOs would regulate the duplex stability, nuclease resistance, RNase H activity, and one base mismatch discrimination. In addition, this synthetic strategy would also work for the synthesis of any other gapmers containing LNA or 2′-*O*-MOE which are widely used sugar modifications of ASOs. Thus, we expect that a PB/PS/PO chimeric ODN is one of the promising candidates of potent ASOs. Hence, the details of the biological and physiological properties of PB/PS/PO chimeric ODNs are under investigation.

## Experimental Section

### General Information

All reactions were conducted under an Ar atmosphere. Dry organic solvents were prepared by appropriate procedures. A thermostatic chamber was used for the reaction run at 50 °C. ^1^H NMR spectra were recorded at 400 MHz with tetramethylsilane (δ 0.0) as an internal standard in CDCl_3_. ^13^C NMR spectra were recorded at 100 MHz with CDCl_3_ (δ 77.0) as an internal standard in CDCl_3_. ^31^P NMR spectra were recorded at 162 MHz with H_3_PO_4_ (δ 0.0) as an external standard in CDCl_3_. Analytical thin-layer chromatography was performed on commercial glass plates with a 0.25 mm thickness silica gel layer. Silica gel column chromatography was performed using spherical, neutral, 63–210 μm silica gel. Manual solid-phase synthesis was carried out using a glass filter (10 mm × 50 mm) with a stopper at the top and a stopcock at the bottom as a reaction vessel. Synthesized dimers were analyzed by reversed-phase HPLC. Synthesized oligomers (tetramer and dodecamers) were analyzed by reversed-phase HPLC and/or anion-exchange HPLC, purified by reverse-phase HPLC or anion-exchange HPLC, and identified by electrospray ionization (ESI) mass spectroscopy. Isolated yields of oligomers were estimated by measuring UV–vis spectra. PO-ODN (**26**), PS-ODN (**27**), PS/PO-ODN (**29**), and cRNA (**30**) were purchased and used for denaturation, nuclease resistance, and RNase H activity tests without further purification.

### General Procedure for the Synthesis of 5′-*O*-Dimethoxytrityl-nucleoside 3′-*H*-phosphonothioates (**5a**, **5c**, **5g**, and **5t**)

Nucleoside **1a** (0.90 g, 1.5 mmol, 1.5 equiv), **1c** (0.98 g, 1.5 mmol, 1.5 equiv), **1g** (0.92 g, 1.5 mmol, 1.5 equiv), or **1t** (0.96 g, 1.5 mmol, 1.5 equiv) was dried by repeated coevaporation with dry pyridine. On the other hand, dry triethylamine was added to phosphinic acid (0.19 mL, 1 mmol, 1.0 equiv) and the solution was dried by coevaporation with dry pyridine. Thereafter, nucleoside and triethylammonium phosphinate were dissolved in the same solvent (5 mL). Pivaloyl chloride (0.17 mL, 1.5 mmol, 1.5 equiv) was added to the solution at 0 °C while stirring. After 15 min, the solution was warmed to rt and after further 5 min, elemental sulfur (48 mg, 1.5 mmol, 1.5 equiv) was added. Stirring was continued for 1 h, and then the mixture was diluted with CHCl_3_ (30 mL) and washed with 1.0 M TEAB buffers (3 × 30 mL). The aqueous layers were combined and back-extracted with CHCl_3_ (3 × 30 mL). The combined organic layers were dried over Na_2_SO_4_, filtered, and concentrated under reduced pressure. The residue was purified by silica gel column chromatography (20 g of neutral silica gel, 2 × 10 cm) using AcOEt–MeOH–Et_3_N and CH_2_Cl_2_-MeOH*-*Et_3_N as the eluent. The fractions containing **5a**, **5c**, **5g**, or **5t** were collected and concentrated under reduced pressure. CHCl_3_ (30 mL) was added to the residue and washed with 0.2 M DBU hydrogen carbonate aqueous solutions. The aqueous layers were combined and back-extracted with CHCl_3_. The organic layers were combined, dried over Na_2_SO_4_, filtered, and concentrated under reduced pressure to yield **5a**, **5c**, **5g**, or **5t**.

#### 1,8-Diazabicyclo [5.4.0] undec-7-enium 5′-*O*-dimethoxytrityl*-N*^6^-benzoyladenosine 3′-*H*-phosphonothioate as a Mixture of (*S*p)- and (*R*p)-Diastereomers (**5a**)

The crude mixture containing **5a** was purified by silica gel column chromatography using AcOEt–MeOH–Et_3_N (100:0:1–100:4:1, v/v/v) and then CHCl_3_–MeOH–Et_3_N (100:2:1, v/v/v) as the eluent. Then, the fractions containing **5a** were collected and concentrated under reduced pressure. CHCl_3_ (30 mL) was added to the residue and washed with 0.2 M DBU hydrogen carbonate aqueous solutions (3 × 30 mL). The aqueous layers were combined and back-extracted with CHCl_3_ (3 × 30 mL). **5a** was obtained as colorless foam (0.88 g, 0.95 mmol, 95% yield). ^1^H NMR (400 MHz, CDCl_3_) δ 11.9–11.7 (br, 1H), 9.2–8.9 (br, 1H), 8.71 (s, 1H), 8.20 (s, 1H), 8.17 (d, *J* = 574 Hz, 0.5H), 8.13 (d, *J* = 579 Hz, 0.5H), 8.01 (d, *J* = 7.3 Hz, 2H), 7.62–7.57 (m, 1H), 7.54–7.48 (m, 2H), 7.46–7.38 (m, 3H), 7.34–7.10 (m, 6H), 6.79 (d, *J* = 8.7 Hz, 4H), 6.65–6.57 (m, 1H), 5.43–5.35 (m, 1H), 4.57–4.54 (m, 0.5H), 4.48–4.44 (m, 0.5H), 3.76 (s, 6H), 3.52–3.36 (m, 8H), 2.98–2.84 (m, 4H), 1.99 (quintet, *J* = 5.8 Hz, 2H), 1.80–1.60 (m, 6H); ^13^C{^1^H} NMR (100 MHz, CDCl_3_) δ 166.0, 164.5, 158.3, 152.4, 151.5, 149.2, 144.5, 141.4, 135.6, 135.6, 133.6, 132.6, 130.0, 128.8, 128.1, 127.8, 127.7, 126.7, 123.2, 113.1, 86.4, 86.4, 86.2 (d, ^3^*J*_C–P_ = 5.8 Hz), 85.5 (d, ^3^*J*_C–P_ = 6.7 Hz), 84.6, 84.5, 75.4 (d, ^2^*J*_C–P_ = 4.8 Hz), 74.8 (d, ^2^*J*_C–P_ = 4.8 Hz), 63.6, 63.5, 55.1, 54.2, 48.6, 39.8, 38.1, 32.2, 29.7, 28.9, 26.8, 24.0, 19.5; ^31^P{^1^H} NMR (162 MHz, CDCl_3_) δ 53.0, 52.9. HRMS (ESI/Q-TOF) *m*/*z* calcd for C_38_H_35_N_5_O_7_PS^–^ [M–DBU–H]^−^, 736.2000; found 736.1982.

#### 1,8-Diazabicyclo [5.4.0] undec-7-enium 5′-*O*-dimethoxytrityl-*N*^4^-isobutyrylcytidine 3′-*H*-phosphonothioate as a Mixture of (*S*p)- and (*R*p)-diastereomers (**5c**)

The crude mixture containing **5c** was purified by silica gel column chromatography using AcOEt–MeOH–Et_3_N (100:0:1–100:4:1, v/v/v) and then CHCl_3_–MeOH–Et_3_N (100:4:1, v/v/v) as the eluent. Then, the fractions containing **5c** were collected and concentrated under reduced pressure. CHCl_3_ (20 mL) was added to the residue and washed with 0.2 M DBU hydrogen carbonate aqueous solutions (3 × 20 mL). The aqueous layers were combined and back-extracted with CHCl_3_ (3 × 20 mL). **5c** was obtained as colorless foam (0.89 g, 0.99 mmol, 99% yield). ^1^H NMR (400 MHz, CDCl_3_) δ 11.7–11.4 (br, 1H), 8.8–8.5 (br, 1H), 8.19 (d, *J* = 5.0 Hz, 0.5H), 8.17 (d, *J* = 5.0 Hz, 0.5H), 8.13 (d, *J* = 574 Hz, 0.5H), 8.10 (d, *J* = 581 Hz, 0.5H), 7.46–7.39 (m, 2H), 7.37–7.20 (m, 7H), 7.13 (d, *J* = 6.0 Hz, 0.5H), 7.11 (d, *J* = 6.0 Hz, 0.5H), 6.90–6.81 (m, 4H), 6.30 (t, *J* = 6.0 Hz, 0.5H), 6.29 (t, *J* = 6.0 Hz, 0.5H), 5.37–5.29 (m, 0.5 H), 5.25–5.17 (m, 0.5 H), 4.48 (q, *J* = 3.0 Hz, 0.5 H), 4.36 (q, *J* = 3.3 Hz, 0.5 H), 3.80 (s, 6H), 3.54–3.39 (m, 8H), 2.92–2.82 (m, 2H), 2.69–2.61 (m, 1H), 2.38–2.26 (m, 1H), 2.00 (quintet, *J* = 5.8 Hz, 2H), 1.82–1.60 (m, 7H), 1.25–1.17 (m, 6H); ^13^C{^1^H} NMR (100 MHz, CDCl_3_) δ 176.7, 166.0, 162.1, 158.4, 155.0, 144.6, 144.1, 135.4, 135.2, 130.0, 129.9, 128.1, 127.9, 126.9, 113.2, 96.0, 87.1, 87.0, 86.7, 86.0 (d, ^3^*J*_C–P_ = 6.7 Hz), 85.3 (d, ^3^*J*_C–P_ = 5.8 Hz), 74.2 (d, ^2^*J*_C–P_ = 4.8 Hz), 73.1 (d, ^2^*J*_C–P_ = 5.8 Hz), 62.6, 62.5, 55.1, 54.2, 48.5, 41.2, 40.8, 38.1, 36.5, 32.3, 28.9, 26.7, 24.0, 19.4, 19.0, 18.9; ^31^P{^1^H} NMR (162 MHz, CDCl_3_) δ 53.6, 52.6. HRMS (ESI/Q-TOF) *m*/*z* calcd for C_34_H_37_N_3_O_8_PS^–^ [M–DBU–H]^−^, 678.2044; found 678.2032.

#### 1,8-Diazabicyclo [5.4.0] undec-7-enium 5′-*O*-dimethoxytrityl-*N*^2^-isobutyrylguanosine 3′-*H*-phosphonothioate as a Mixture of (*S*p)- and (*R*p)-diastereomers (**5g**)

The crude mixture containing **5g** was purified by silica gel column chromatography twice using CHCl_3_–MeOH–Et_3_N (100:2:1–100:4:1, v/v/v) as the eluent for the first time, and AcOEt–MeOH–Et_3_N (100:2:1, v/v/v) and then CHCl_3_–MeOH–Et_3_N (100:3:1–100:5:1, v/v/v) as the eluent for the second time. Then, the fractions containing **5g** were collected and concentrated under reduced pressure. CHCl_3_ (20 mL) was added to the residue and washed with 0.2 M DBU hydrogen carbonate aqueous solutions (3 × 20 mL). The aqueous layers were combined and back-extracted with CHCl_3_ (3 × 20 mL). **5g** was obtained as colorless foam (0.58 g, 0.64 mmol, 64% yield). ^1^H NMR (400 MHz, CDCl_3_) δ 8.12 (d, *J* = 581 Hz, 0.5H), 8.05 (d, *J* = 574 Hz, 0.5H), 7.82 (s, 0.5H), 7.80 (s, 0.5H), 7.44–7.31 (m, 3H), 7.31–7.22 (m, 4H), 7.22–7.13 (m, 2H), 6.79–6.67 (m, 4H), 6.18 (t, *J* = 6.0 Hz, 0.5H), 6.18 (t, *J* = 6.4 Hz, 0.5H), 5.75–5.62 (m, 0.5H), 5.62–5.51 (m, 0.5H), 4.37 (q, *J* = 3.7 Hz, 0.5H), 4.26 (q, *J* = 3.8 Hz, 0.5H), 3.75 (s, 6H), 3.50–3.25 (m, 8H), 3.02–2.83 (m, 1H), 2.83–2.74 (m, 2H), 2.74–2.58 (m, 2H), 2.03–1.87 (m, 2H), 1.83–1.53 (m, 6H), 1.20–1.07 (m, 6H); ^13^C{^1^H} NMR (100 MHz, CDCl_3_) δ 179.8, 166.0, 158.3, 155.9, 148.1, 148.0, 147.7, 147.6, 144.6, 144.6, 138.2, 137.9, 135.6, 130.0, 130.0, 128.1, 128.1, 127.7, 126.7, 121.3, 121.2, 113.0, 86.2, 86.1, 85.4 (d, ^3^*J*_C–P_ = 5.8 Hz), 84.6 (d, ^3^*J*_C–P_ = 5.8 Hz), 84.1, 84.1, 74.4, 73.5, 70.5, 63.4, 63.1, 55.1, 54.3, 48.6, 46.0, 39.0, 38.1, 35.8, 35.8, 32.4, 28.9, 26.7, 23.9; ^31^P{^1^H} NMR (162 MHz, CDCl_3_) δ 52.8, 52.5. HRMS (ESI/Q-TOF) *m*/*z* calcd for C_35_H_37_N_5_O_8_PS^–^ [M–DBU–H]^−^, 718.2106; found 718.2087.

#### 1,8-Diazabicyclo [5.4.0] undec-7-enium 5′-*O*- dimethoxytrityl-*N*^3^-benzoylthymidine 3′-*H*-phosphonothioate as a Mixture of (*S*p)- and (*R*p)-diastereomers (**5t**)

The crude mixture containing **5t** was purified by silica gel column chromatography using AcOEt–MeOH–Et_3_N (100:0:1–100:1:1, v/v/v) and then CHCl_3_–MeOH–Et_3_N (100:0:1–100:3:1, v/v/v) as the eluent. Then, the fractions containing **5t** were collected and concentrated under reduced pressure. CHCl_3_ (20 mL) was added to the residue and washed with 0.2 M DBU hydrogen carbonate aqueous solutions (3 × 20 mL). The aqueous layers were combined and back-extracted with CHCl_3_ (2 × 20 mL). **5t** was obtained as yellow foam (0.85 g, 0.95 mmol, 95% yield). ^1^H NMR (400 MHz, CDCl_3_) δ 11.63–11.49 (br, 1H), 8.13 (d, *J* = 572 Hz, 0.5H), 8.08 (d, *J* = 578 Hz, 0.5H), 7.92 (d, *J* = 8.2 Hz, 2H), 7.78 (s, 0.5H), 7.77 (s, 0.5H), 7.64 (t, *J* = 7.3 Hz, 1H), 7.53–7.39 (m, 5H), 7.38–7.27 (m, 5H), 7.26–7.18 (m, 1H), 6.91–6.82 (m, 4H), 6.44 (t, *J* = 8.7 Hz, 0.5H), 6.43 (t, *J* = 8.9 Hz, 0.5H), 5.51–5.41 (m, 0.5H), 5.41–5.32 (m, 0.5H), 4.41 (d, *J* = 1.4 Hz, 0.5H), 4.30 (d, *J* = 2.3 Hz, 0.5H), 3.79 (s, 6H), 3.63–3.46 (m, 1H), 3.45–3.31 (m, 7H), 2.87–2.62 (m, 3H), 2.54–2.38 (m, 1H), 1.93 (quintet, *J* = 5.8 Hz, 2H), 1.78–1.54 (m, 6H), 1.37 (d, *J* = 6.9 Hz, 3H); ^13^C{^1^H} NMR (100 MHz, CDCl_3_) δ 169.1, 165.9, 162.8, 158.5, 158.5, 149.2, 144.3, 144.2, 135.7, 135.4, 135.3, 135.2, 135.1, 134.9, 131.5, 130.3, 130.0, 129.0, 128.2, 127.9, 126.9, 113.2, 111.0, 111.0, 86.9, 86.0 (d, ^3^*J*_C–P_ = 5.8 Hz), 85.2 (d, ^3^*J*_C–P_ = 6.7 Hz), 84.8, 75.5 (d, ^2^*J*_C–P_ = 4.8 Hz), 74.6 (d, ^3^*J*_C–P_ = 4.8 Hz), 63.5, 63.4, 55.1, 54.1, 48.4, 40.2 (d, ^3^*J*_C–P_ = 2.9 Hz), 39.7 (d, ^3^*J*_C–P_ = 2.9 Hz), 38.0, 32.1, 28.8, 26.6, 23.9, 19.3, 11.4; ^31^P{^1^H} NMR (162 MHz, CDCl_3_) δ 54.5, 53.4. HRMS (ESI/Q-TOF) *m*/*z* calcd for C_38_H_36_N_2_O_9_PS^–^ [M–DBU–H]^−^, 727.1885; found 727.1884.

### General Procedure for the Synthesis of 5′-*O*-Dimethoxytrityl-2′-*O*-methyl-nucleoside 3′-*H-*boranophosphonate

Nucleoside **2a** (0.69 g, 1.0 mmol, 1.0 equiv), **2c** (0.68 g, 1 mmol, 1.0 equiv), **2g** (0.70 g, 1 mmol, 1.0 equiv), or **2u** (0.67 g, 1 mmol, 1.0 equiv) and pyridinium *H-*boranophosphonate (0.28 g, 2.0 mmol, 2.0 equiv) were dried individually by repeated coevaporation with dry pyridine and dissolved together in dry pyridine (25 mL) at 0 °C under argon. Bis(2-oxo-3-oxazolidinyl) phosphinic chloride (Bop-Cl) (0.51 g, 2.0 mmol, 2.0 equiv) was added at 0 °C, and the mixture was stirred for 1 h at 0 °C. The mixture was warmed to rt and diluted with CH_2_Cl_2_ (30 mL) and washed with 1 M triethylammonium bicarbonate (TEAB) buffers (pH 7) (3 × 30 mL). The aqueous layers were combined and back-extracted with CH_2_Cl_2_ (3 × 30 mL). The organic layers were combined, dried over Na_2_SO_4_, filtered, and concentrated under reduced pressure. The residue was then purified by silica gel column chromatography (20 g of neutral silica gel) using AcOEt–MeOH–Et_3_N and CHCl_3_–MeOH–Et_3_N as the eluent. The residue was dissolved in CHCl_3_ (30 mL for **6a**, **6c**, and **6g**) or CH_2_Cl_2_ (30 mL for **6u**) and washed with 1 M TEAB buffers (3 × 30 mL). The aqueous layers were combined and back-extracted with CHCl_3_ (3 × 30 mL for **6a**, **6c**, and **6g**) or CH_2_Cl_2_ (30 mL for **6u**). The organic layers were combined, dried over Na_2_SO_4_, filtered, and concentrated under reduced pressure to yield **6a**, **6c**, **6g**, or **6u**

#### Triethylammonium 5′-*O*-Dimethoxytrityl*-N*^6^-benzoyl-2′-*O*-methyladenosine 3′-*H-*boranophosphonate as a Mixture of (*S*p)- and (*R*p)-diastereomers (**6a**)

The crude mixture containing **6a** was purified by silica gel column chromatography using AcOEt–MeOH–Et_3_N (100:0:1–100:4:1, v/v/v) and then CHCl_3_–MeOH–Et_3_N (100:4:1, v/v/v) as the eluent. **6a** was obtained as colorless foam (0.81 g, 0.95 mmol, 95%). ^1^H NMR (400 MHz, CDCl_3_) δ 9.09 (s, 1H), 8.70 (s, 0.5H), 8.69 (s, 0.5H), 8.24 (s, 0.5H), 8.20 (s, 0.5H), 8.02 (d, *J* = 7.8 Hz, 2H), 7.9–7.8 (br, 0.5H), 7.60 (t, *J* = 7.3 Hz, 1H), 7.51 (t, *J* = 7.8 Hz, 2H), 7.48–7.42 (m, 2H), 7.36–7.31 (m, 4H), 7.30–7.23 (m, 2H), 7.23–7.17 (m, 1H), 6.84–6.78 (m, 4H), 6.27 (t, *J* = 5.0 Hz, 0.5H), 6.27 (t, *J* = 5.0 Hz, 0.5H), 5.09–5.02 (m, 1H), 4.74–4.68 (m, 1H), 4.55–4.50 (m, 1H), 3.77 (s, 6H), 3.55–3.48 (m, 5H), 2.99 (q, *J* = 7.3 Hz, 6H), 1.27 (t, *J* = 7.3 Hz, 9H), 0.9–0.1 (br, 3H); ^13^C{^1^H} NMR (100 MHz, CDCl_3_) δ 164.5, 158.4, 152.5, 151.8, 151.7, 149.3, 144.4, 141.7, 135.6, 135.6, 135.5, 135.5, 133.6, 132.7, 130.0, 128.8, 128.1, 128.1, 127.8, 127.8, 127.7, 126.8, 123.4, 113.1, 113.1, 86.6, 86.5, 86.4, 86.4, 84.1 (d, ^3^*J*_C–P_ = 2.9 Hz), 84.0 (d, ^3^*J*_C–P_ = 2.9 Hz), 82.3 (d, ^3^*J*_C–P_ = 2.9 Hz), 81.9 (d, ^3^*J*_C–P_ = 2.9 Hz), 73.1 (d, ^2^*J*_C–P_ = 5.8 Hz), 72.2 (d, ^2^*J*_C–P_ = 5.8 Hz), 63.0, 62.8, 58.5, 58.4, 55.1, 45.2, 8.4; ^31^P{^1^H} NMR (162 MHz, CDCl_3_) δ 108.8–103.9 (br). HRMS (ESI/Q-TOF) *m*/*z* calcd for C_39_H_40_BN_5_O_8_P^–^ [M–Et_3_N–H]^−^, 748.2713; found 748.2691.

#### Triethylammonium 5′-*O*-Dimethoxytrityl-*N*^4^-benzoyl-2′-*O*-methylcytidine 3′-*H-*boranophosphonate as a Mixture of (*S*p)- and (*R*p)-diastereomers (**6c**)

The crude mixture containing **6c** was purified by silica gel column chromatography using AcOEt–MeOH–Et_3_N (100:0:1–100:4:1, v/v/v) and then CHCl_3_–MeOH–Et_3_N (100:4:1, v/v/v) as the eluent. **6c** was obtained as colorless foam (0.75 g, 0.90 mmol, 90%). ^1^H NMR (400 MHz, CDCl_3_) δ 8.65 (d, *J* = 7.8 Hz, 0.5H), 8.59 (d, *J* = 7.3 Hz, 0.5H), 7.95–7.86 (m, 2H), 7.86–7.77 (br, 0.5H), 7.51 (t, *J* = 7.8 Hz, 2H), 7.44 (dd, *J* = 7.1, 5.7 Hz, 2H), 7.39–7.24 (m, 7H), 7.20–7.02 (br, 0.5H), 6.92–6.85 (m, 4H), 6.05 (s, 0.5H), 6.02 (s, 0.5H), 5.05–4.92 (m, 0.5H), 4.82–4.75 (m, 0.5H), 4.40 (dd, *J* = 2.1, 8.9 Hz, 1H), 4.12–4.03 (m, 1H), 3.83 (s, 6H), 3.67 (s, 3H), 3.63–3.53 (m, 2H), 3.01 (q, *J* = 7.2 Hz, 6H), 1.27 (t, *J* = 7.3 Hz, 9H), 0.90–0.10 (br, 3H); ^13^C{^1^H} NMR (100 MHz, CDCl_3_) δ 166.1, 162.1, 158.5, 145.0, 144.0, 135.6, 135.5, 135.2, 135.1,133.1, 133.0, 130.2, 130.0, 130.0, 129.9, 128.9, 128.3, 128.1, 128.0, 128.0, 127.4, 127.0, 113.3, 113.2, 96.5, 96.4, 88.9, 88.7, 86.9, 86.9, 83.3, 82.7, 81.4 (d, ^3^*J*_C–P_ = 5.8 Hz), 81.3 (d, ^3^*J*_C–P_ = 5.2 Hz), 72.9 (d, ^2^*J*_C–P_ = 9.6 Hz), 69.2 (d, ^2^*J*_C–P_ = 4.8 Hz), 60.5, 58.5, 58.4, 55.1, 45.3, 8.4; ^31^P{^1^H} NMR (162 MHz, CDCl_3_) δ 111.8–102.0. HRMS (ESI/Q-TOF) *m*/*z* calcd for C_38_H_40_BN_3_O_9_P^–^ [M–Et_3_N–H]^−^, 724.2601; found 724.2591.

#### Triethylammonium 5′-*O*-Dimethoxytrityl-*N*^2^-isobutyryl-2′-*O*-methylguanosine 3′-*H-*boranophosphonate as a Mixture of (*S*p)- and (*R*p)-diastereomers (**6g**)

The crude mixture containing **6g** was purified by silica gel column chromatography using AcOEt–MeOH–Et_3_N (100:2:1, v/v/v) and then CHCl_3_–MeOH–Et_3_N (100:3:1–100:5:1, v/v/v) as the eluent. **6g** was obtained as colorless foam (0.78 g, 0.92 mmol, 92%). ^1^H NMR (400 MHz, CDCl_3_) δ 12.54–11.63 (br, 1H), 10.47–9.66 (br, 1H), 7.88 (s, 0.5H), 7.87 (s, 0.5H), 7.81–7.67 (br, 0.5 H), 7.53–7.36 (m, 2H), 7.34–7.28 (m, 4H), 7.26–7.13 (m, 3H), 6.93–6.83 (br, 0.5H), 6.83–6.66 (m, 4H), 5.93 (d, *J* = 4.6 Hz, 0.5H), 5.90 (d, *J* = 4.6 Hz, 0.5H), 5.37–5.29 (m, 0.5H), 5.29–5.20 (m, 0.5H), 4.58 (t, *J* = 4.8 Hz, 0.5H), 4.56 (t, *J* = 4.8 Hz, 0.5H), 4.44–4.36 (m, 0.5H), 4.36–4.28 (m, 0.5H), 3.75, 3.75, 3.74 (s, s, s, 6H), 3.54–3.37 (m, 4H), 3.37–3.22 (m, 1H), 3.00 (q, *J* = 7.2 Hz, 6H), 2.62–2.40 (m, 1H), 1.25 (t, *J* = 7.3 Hz, 9H), 1.11 (d, *J* = 6.9 Hz, 3H), 1.04 (d, *J* = 5.5 Hz, 3H), 0.95–0.10 (br, 3H); ^13^C{^1^H} NMR (100 MHz, CDCl_3_) δ179.6, 179.5, 158.3, 158.3, 155.7, 148.2, 147.6, 147.6, 144.5, 138.1, 137.9, 138.1, 137.9, 135.6, 135.5, 135.4 129.8, 127.9, 127.6, 127.6 126.6, 126.6, 121.3, 121.2, 112.9, 112.8, 86.5, 86.3, 86.2, 86.1, 83.2, 82.9, 82.3, 82.2, 72.5 (d, ^2^*J*_C–P_ = 4.8 Hz), 72.0 (d, ^2^*J*_C–P_ = 3.9 Hz), 62.7, 62.4, 58.4, 55.0, 45.4, 35.6, 35.6, 18.6, 8.5; ^31^P{^1^H} NMR (162 MHz, CDCl_3_) δ 108.0–102.0. HRMS (ESI/Q-TOF) *m*/*z* calcd for C_36_H_42_BN_5_O_9_P^–^ [M–Et_3_N–H]^−^, 730.2819; found 730.2791.

#### Triethylammonium 5′-*O*-Dimethoxytrityl-*N*^3^-benzoyl-2′-*O*-methyluridine 3′-*H-*boranophosphonate as a Mixture of (*S*p)- and (*R*p)-diastereomers (**6u**)

The crude mixture containing **6u** was purified by silica gel column chromatography using AcOEt–MeOH–Et_3_N (100:0:1, v/v/v) and then CHCl_3_–MeOH–Et_3_N (100:0:1–100:1:1, v/v/v) as the eluent. **6u** was obtained as colorless foam (0.62 g, 0.74 mmol, 74%). ^1^H NMR (400 MHz, CDCl_3_) δ 8.23 (d, *J* = 8.2 Hz, 0.5H), 8.17 (d, *J* = 8.2 Hz, 0.5H), 8.01–7.90 (m, 2H), 7.65 (t, *J* = 7.3 Hz, 1H), 7.51 (t, *J* = 7.8 Hz, 2H), 7.47–7.38 (m, 2H), 7.38–7.21 (m, 7H), 6.87 (m, 4H), 5.97 (d, *J* = 2.3 Hz, 0.5 H) 5.97 (d, *J* = 2.1 Hz, 0.5 H), 5.30 (d, *J* = 2.7 Hz, 0.5H), 5.27 (d, *J* = 2.7 Hz, 0.5H), 5.10–5.03 (m, 0.5 H), 4.95–4.86 (m, 0.5 H), 4.36 (m, 1H), 4.15–3.91 (m, 1H), 3.80 (s, 6H), 3.73–3.58 (m, 1H), 3.55 (d, *J* = 4.6 Hz, 4H), 3.14–2.91 (m, 6H), 1.44–1.18 (m, 9H), 1.03–0.16 (m, 3H); ^13^C{^1^H} NMR (100 MHz, CDCl_3_) δ 168.9, 168.8, 162.0, 158.6, 158.6, 149.1, 144.2, 144.2, 140.1, 135.2, 135.1, 135.1, 134.9, 134.8, 131.3, 130.5, 130.2, 130.1, 130.0, 129.1, 128.2, 128.0, 128.0, 127.1, 113.3, 101.8, 101.8, 87.7, 87.1, 83.4, 82.9, 82.1, 73.1 (d, ^2^*J*_C–P_ = 8.7 Hz), 69.9 (d, ^2^*J*_C–P_ = 4.8 Hz), 61.0, 60.5, 58.4, 58.3, 55.2, 45.3, 8.5; ^31^P{^1^H} NMR (162 MHz, CDCl_3_) δ 111.0–103.0. HRMS (ESI/Q-TOF) *m*/*z* calcd for C_38_H_39_BN_2_O_10_P^–^ [M–Et_3_N–H]^−^, 725.2441; found 725.2427.

### General Procedure for the Manual Solid-Phase Synthesis of T_PS_T Dimer (Table 1)

HCP-loaded 5′-*O*-DMTr-*N*^3^-benzoylthymidine, via a succinyl linker (0.5 μmol), in a reaction vessel was treated with 3% DCA in dry CH_2_Cl_2_ (5 × 12 s, 1 mL each) and washed with dry CH_2_Cl_2_ (4 × 1 mL) and CH_3_CN (4 × 1 mL). Thereafter, it was dried *in vacuo* for 10 min. To the reaction vessel, the 3′-*H*-phosphonothioate thymidine monomer **5t** (19.7 mg, 20 μmol, 40 equiv, 0.1 M) and condensing reagent (50 μmol, 100 equiv, 0.25 M) (BOMP, 23.3 mg, for entry 1; MNTP, 22.3 mg for entry 2, PyNTP, 25.0 mg for entries 3–7) were added. Then, a solution of 2,6-lutidine (0.6 M, 120 μmol for entry 1–3), pyridine (0.6 M, 120 μmol for entry 4), quinoline (0.6 M, 120 μmol for entry 5, 1.8 M, 355 μmol for entry 6), or without base (entry 7) in dry CH_3_CN (200 μL) was added under Ar, and the reaction vessel was stirred slowly with hands for 3 min. The HCP was washed with dry CH_3_CN (4 × 1 mL) and CH_2_Cl_2_ (4 × 1 mL), and the detritylation reaction was carried out by treatment with 3% DCA in dry CH_2_Cl_2_–Et_3_SiH (1:1, v/v) (5 × 12 s, 1 mL each). Thereafter, the HCP was washed with dry CH_2_Cl_2_ (4 × 1 mL) and CH_3_CN (4 × 1 mL), and dried *in vacuo* for 10 min. The *H*-phosphonothioate internucleotide linkage was oxidized by treatment with a solution (500 μL) of 0.1 M TEA in CCl_4_–2,6-lutidine–H_2_O (5:12.5:1, v/v/v) for 90 min. The HCP was washed with dry CH_3_CN (4 × 1 mL) and CH_2_Cl_2_ (4 × 1 mL), and dried *in vacuo*. The HCP was treated with concentrated aqueous NH_3_–EtOH (3:1, v/v, 5 mL) at rt for 3 h, filtered, and washed with EtOH (3 × 1 mL). The filtrate and washings were combined and concentrated under reduced pressure. The residue was analyzed by RP-HPLC. RP-HPLC was performed with a linear gradient of 0–30% CH_3_CN for 60 min in 0.1 M triethylammonium acetate (TEAA) buffer (pH 7.0) at 30 °C with a flow rate of 0.5 mL/min using a C18 column (100 Å, 3.9 mm × 150 mm).

### General Procedure for the Manual Solid-Phase Synthesis of N_PS_T Dimers (Table 2)

HCP-loaded 5′-*O*-DMTr-*N*^3^-benzoylthymidine, via a succinyl linker (0.5 μmol, 30.65 μmol/g), in a reaction vessel was treated 3% DCA in dry CH_2_Cl_2_ (5 × 12 s, 1 mL each) and washed with dry CH_2_Cl_2_ (4 × 1 mL) and CH_3_CN (4 × 1 mL). Thereafter, it was dried *in vacuo* for 10 min. To the reaction vessel, the 3′-*H*-phosphonothioate monomer unit (**5a** (19.0 mg, 20 μmol, 40 equiv, 0.1 M), **5c** (17.8 mg, 20 μmol, 40 equiv, 0.1 M), or **5g** (18.1 mg, 20 μmol, 40 equiv, 0.1 M for entries 5 and 6, 40 μmol, 80 equiv, 0.2 M for entry 7)) and PyNTP (25 mg, 50 μmol, 100 equiv, 0.25 M for entries 1–6; 50 mg, 100 μmol, 200 equiv, 0.5 M for entry 7) as condensing reagent were added. Then, a solution (200 μL) of quinoline (1.8 M, 355 μmol for entries 1, 3, and 5) or without base (for entries 2, 4, 6, and 7) in dry CH_3_CN was added under Ar, and the reaction vessel was stirred slowly with hands for 3 min. The HCP was washed with dry CH_3_CN (4 × 1 mL) and CH_2_Cl_2_ (4 × 1 mL), and the detritylation reaction was carried out by treatment with 3% DCA in dry CH_2_Cl_2_–Et_3_SiH (1:1, v/v) (4 × 15 s, 1 mL each). Therefore, the HCP was washed with dry CH_2_Cl_2_ (4 × 1 mL) and CH_3_CN (4 × 1 mL), and dried *in vacuo* for 10 min. The *H*-phosphonothioate internucleotide linkage was oxidized by treatment with a solution (500 μL) of 0.1 M TEA in CCl_4_–2,6-lutidine–H_2_O (5:12.5:1, v/v/v) for 90 min. The HCP was washed with dry CH_3_CN (4 × 1 mL) and CH_2_Cl_2_ (4 × 1 mL), and dried *in vacuo*. The HCP was treated with concentrated aqueous NH_3_–EtOH (3:1, v/v, 5 mL) at rt for 12 h (entries 1–4) or at 50 °C in a thermostatic chamber for 12 h (entries 5–7), filtered, and washed with EtOH (3 × 1 mL). The filtrate and washings were combined and concentrated under reduced pressure. The residue was analyzed by RP-HPLC. RP-HPLC was performed with a linear gradient of 0–30% CH_3_CN for 60 min in 0.1 M triethylammonium acetate (TEAA) buffer (pH 7.0) at 30 °C with a flow rate of 0.5 mL/min using a C18 column (100 Å, 3.9 mm × 150 mm).

### General Procedure for the Manual Solid-Phase Synthesis of N_PB_T Dimers Using 5′-*O*-Dimethoxytrityl-2′-*O*-methyl nucleoside 3′-*H-*boranophosphonate (Table 3)

HCP-loaded 5′-*O*-DMTr-*N*^3^-benzoylthymidine, via a succinyl linker (0.5 μmol, 30.65 μmol/g), in a reaction vessel was treated 3% DCA in dry CH_2_Cl_2_ (5 × 12 s, 1 mL each) and washed with dry CH_2_Cl_2_ (4 × 1 mL) and CH_3_CN (4 × 1 mL). Thereafter, it was dried *in vacuo* for 10 min. To the reaction vessel, the 2′-*O*-methyl-nucleoside 3′-*H-*boranophosphonate monomer unit (**6a** (17.1 mg, 20 μmol, 40 equiv, 0.1 M), **6c** (16.7 mg, 20 μmol, 40 equiv, 0.1 M), **6g** (16.9 mg, 20 μmol, 40 equiv, 0.1 M), or **6u** (16.7 mg, 20 μmol, 40 equiv, 0.1 M)) and MNTP (22.3 mg, 50 μmol, 100 equiv) as a condensing reagent were added and 2,6-lutidine (0.6 M, 120 μmol) in dry CH_3_CN (200 μL) was added under Ar, and the reaction vessel was stirred slowly with hands for 3 min. The HCP was washed with dry CH_3_CN (4 × 1 mL) and CH_2_Cl_2_ (4 × 1 mL), and the detritylation reaction was carried out by treatment with 3% DCA in dry CH_2_Cl_2_–Et_3_SiH (1:1, v/v) (4 × 15 s, 1 mL each). Therefore, the HCP was washed with dry CH_2_Cl_2_ (4 × 1 mL) and CH_3_CN (4 × 1 mL), and dried *in vacuo* for 10 min. The *H-*boranophosphonate internucleotide linkage was oxidized by treatment with a solution (500 μL) of 0.1 M TEA in CCl_4_–2,6-lutidine–H_2_O (5:12.5:1, v/v/v) for 90 min. The HCP was washed with dry CH_3_CN (4 × 1 mL) and CH_2_Cl_2_ (4 × 1 mL), and dried *in vacuo*. The HCP was treated with concentrated aqueous NH_3_–EtOH (3:1, v/v, 5 mL) at rt for 12 h (entries 1 and 2), at 50 °C in a thermostatic chamber for 12 h (entry 3), or rt for 3 h (entry 4), filtered, and washed with EtOH (3 × 1 mL). The filtrate and washings were combined and concentrated under reduced pressure. The residue was analyzed by RP-HPLC. RP-HPLC was performed with a linear gradient of 0–30% CH_3_CN for 60 min in 0.1 M triethylammonium acetate (TEAA) buffer (pH 7.0) at 30 °C with a flow rate of 0.5 mL/min using a C18 column (100 Å, 3.9 mm × 150 mm).

HRMS (ESI/Q-TOF): *m/z* calcd for A_PB_T (**15**) [M–H]^−^, 582.1890; found 582.1879. calcd for C_PB_T (**16**) [M–H]^−^, 558.1778; found 558.1765. calcd for G_PB_T (**17**) [M–H]^−^, 598.1839; found 598.1824. calcd for U_PB_T (**18**) [M–H]^−^, 559.1618; found 559.1607.

#### Synthesis of PB/PS/PO Chimeric Tetramer ODNs and Chimeric Dodecamer ODNs Containing PB Modifications (PB/PS/PO, PB/PO, PB/PS-ODNs, and PB/PS/PO-gapmer) (Table 4)

HCP-loaded 5′-*O*-DMTr-*N*^3^–benzoylthymidine (0.5 μmol, 30.65 μmol/g, entries 1–3), 5′-*O*-DMTr-*N*^4^-isobutyrylcytidine (0.5 μmol, 22.03 μmol/g, entries 4–6) or 5′-*O*-DMTr-2′-*O*-methyl *N*^4^-benzoylcytidine (0.5 μmol, 30.78 μmol/g, entry 7) via a succinyl linker, was treated with 3% DCA in dry CH_2_Cl_2_–Et_3_SiH (1:1, v/v, 4 × 15 s, 1 mL each) and washed with dry CH_2_Cl_2_ (4 × 1 mL) and CH_3_CN (4 × 1 mL), and then dried *in vacuo* for 10 min. Chain elongations were conducted by repeating steps (a) and (b) 3 times (for the synthesis of **19** and **20**) or 11 times (for the synthesis of **21**–**25**).

(a) Condensation step: to introduce a PB, PS, and PO linkages, the *H-*boranophosphonate, *H*-phosphonothioate, or *H*-phosphonate monomer and the condensing reagent were added to the reaction vessel and a condensation reaction was performed with optimized condensation conditions as shown in Table 7 in CH_3_CN for 3 min.

**Table 7:** Condensation Reaction Conditions of Each Monomer

monomer	quantity	condensing reagent	quantity	base	concentration
**3a, c, g**, and **t**	40 equiv, 20 μmol	MNTP	100 equiv, 50 μmol	2,6-lutidine	0.6 M
**4a, c, g**, and **t**	40 equiv, 20 μmol	MNTP	100 equiv, 50 μmol	2,6-lutidine	0.6 M
**5a, c**	40 equiv, 20 μmol	PyNTP	100 equiv, 50 μmol	quinoline	1.8 M
**5g**	80 equiv, 40 μmol	PyNTP	200 equiv, 100 μmol		
**5t**	40 equiv, 20 μmol	PyNTP	100 equiv, 50 μmol		
**6a, c, g**, and **u**	40 equiv, 20 μmol	MNTP	100 equiv, 50 μmol	2,6-lutidine	0.6 M

(b) Detritylation step: to the reaction vessel, 3% DCA in dry CH_2_Cl_2_–Et_3_SiH (1:1, v/v) (4 × 15 s, 1 mL) was added, and the HCP was washed with dry CH_2_Cl_2_ (4 × 1 mL) and CH_3_CN (4 × 1 mL), and then dried *in vacuo* for 10 min.

After the designed length was achieved, the resultant internucleotide linkages were oxidized with a solution (500 μL) of 0.1 M Et_3_N in CCl_4_–2,6-lutidine–H_2_O (5:12.5:1, v/v/v) for 90 min. The HCP was washed with dry CH_2_Cl_2_ (4 × 1 mL) and CH_3_CN (4 × 1 mL), and then dried *in vacuo* for 10 min. The HCP was treated with concentrated aqueous NH_3_–EtOH (3:1, v/v, 5 mL) at 50 °C in a thermostatic chamber for at least 20 h, filtered, and washed with EtOH. The filtrate and washings were combined and concentrated under reduced pressure. The residue was analyzed by RP-HPLC or anion-exchange HPLC, and then purified by anion-exchange HPLC (for **22**) or reverse-phase HPLC (for **21**, **23**–**25**). RP-HPLC was performed with a linear gradient of 5–40% MeOH in a buffer containing 100 mM hexafluoroisopropanol (HFIP) and 8 mM triethylamine at 60 °C for 20 min with a flow rate of 0.5 mL/min. Anion-exchange HPLC was performed with a linear gradient of 0–0.5 M NaCl of 30% *i*POH in a 10 mM Tris-HCl buffer (pH 7.5) at 30 °C for 40 min.

Isolated yields: 6% (d(C_PS_A_PS_G_PS_T_PS_C_PB_A_PB_G_PB_T_PB_C_PO_A_PO_G_PO_T) (**21**)); 19% (d(G_PB_C_PS_A_PB_T_PO_T_PO_G_PO_G_PO_T_PS_A_PB_T_PS_T_PB_C) (**22**)); 5% (d(G_PB_C_PS_A_PB_T_PS_T_PS_G_PB_G_PB_T_PS_A_PB_T_PS_T_PB_C) (**23**)); 19% (d(G_PB_C_PB_A_PB_T_PO_T_PO_G_PO_G_PO_T_PB_A_PB_T_PB_T_PB_C) (**24**)); 13% (G_PB_C_PB_A_PB_ d(T_PS_T_PO_G_PO_G_PO_T_PS_A_PB_)U_PB_U_PB_C (**25**)); HRMS (ESI/Q-TOF): *m/z* calcd for d(C_PS_A_PB_G_PO_T) (**19**) [M–2H]^2–^ 592.6236; found 592.6209. calcd for d(C_PO_A_PB_G_PS_T) (**20**) [M–2H]^2–^, 592.6236; found 592.6217. calcd for d(C_PS_A_PS_G_PS_T_PS_C_PB_A_PB_G_PB_T_PB_C_PO_A_PO_G_PO_T) (**21**) [M–6H]^6–^, 615.7786; found 615.7767. calcd for d(G_PB_C_PS_A_PB_T_PO_T_PO_G_PO_G_PO_T_PS_A_PB_T_PS_T_PB_C) (**22**) [M–6H]^6–^, 614.1138; found 614.1136. calcd for d(G_PB_C_PS_A_PB_T_PS_T_PS_G_PB_G_PB_T_PS_A_PB_T_PS_T_PB_C) (**23**) [M–6H]^6–^, 618.6194; found 618.6175. calcd for d(G_PB_C_PB_A_PB_T_PO_T_PO_G_PO_G_PO_T_PB_A_PB_T_PB_T_PB_C) (**24**) [M–6H]^6–^, 604.9781; found 604.9774. calcd for G_PB_C_PB_A_PB_ d(T_PS_T_PO_G_PO_G_PO_T_PS_A_PB_)U_PB_U_PB_C (**25**) [M–6H]^6–^, 635.9695; found. 635.9670.

### Thermal Denaturation Study

A solution containing pairs of complementary strands (4.0 μM, 0.6 nmol each) and 100 mM NaCl in 10 mM NaH_2_PO_4_–Na_2_HPO_4_ buffer (pH 7.0) was heated for 10 min at 95 °C and cooled to 0 °C at a rate of 0.5 °C/min, and then left at 0 °C for 10 min. Denaturation tests were carried out in a 1 cm path length quartz cell. The UV absorbance values at 260 nm were recorded at a rate of 0.2 °C/min from 0 to 95 °C. The *T*_m_ value was determined from the peak value of the first derivative of the thermal melting curve. The sequence of cRNA was r(G_PO_A_PO_A_PO_U_PO_A_PO_C_PO_C_PO_A_PO_A_PO_U_PO_G_PO_C) or r(G_PO_A_PO_A_PO_C_PO_A_PO_C_PO_C_PO_A_PO_A_PO_U_PO_G_PO_C); subscript PO = phosphate.

### Nuclease Resistance Assay

In the nuclease resistance assay, snake venom phosphodiesterase (SVPDE) from *C. adamanteus* was used. An aqueous solution of SVPDE solution (4 × 10^–4^ U in 45 μL) and a 200 mM Tris-HCl buffer (pH 8.5) at 37 °C containing 30 mM MgCl_2_ (50 μL) were successively added to 0.1 mM aqueous solution of each ODNs (5 μL, 0.5 nmol). After 12 h, the solution was heated to 95 °C for 1 min to denaturate SVPDE and then cooled to 4 °C. The mixture was diluted with 0.1 M TEAA buffer (80 μL) and CH_3_CN (20 μL) and then analyzed using RP-HPLC. RP-HPLC was performed with a linear gradient of 0–40% CH_3_CN for 60 min in 0.1 M TEAA buffer (pH 7.0) at 30 °C with a flow rate of 0.5 mL/min.

### RNase H Activity Evaluation

Cleavage experiments of cRNA by RNase H were conducted using 0.5 μM ODN (**22**–**29**, 0.05 nmol) and 5 μM cRNA (**30**, 0.5 nmol) in a 10 mM Tris buffer containing 100 mM NaCl, 0.5 mM MgCl_2_, and 0.1 mM benzamide as an internal standard at pH 7.2, and the final volume of the solution was 100 μL. A solution of each one of ODNs (**22**–**29**) and 10 equiv of cRNA in the buffer was heated for 10 min at 95 °C, cooled to 0 °C at a rate of 0.5 °C/min, and then left at 37 °C for 10 min. A solution of 60 U/μL RNase H from *E. coli* in the buffer was added to the solution to afford a final concentration of 50 or 25 U/mL and left at 37 °C for 30 min. RNase H was denatured by heating at 95 °C for 1 min, and then the mixture was cooled and analyzed by RP-HPLC. RP-HPLC was performed with a linear gradient of 0–11% MeCN over 44 min followed by 11–40% over 16 min in 0.1 M TEAA buffer (pH 7.0) at 50 °C with a flow rate of 0.5 mL/min.

### RNase H Activity Evaluation (One Base Mismatch)

Cleavage experiments of cRNA by RNase H were conducted using 0.5 μM ODN (**25** or **26**, 0.05 nmol) and 5 μM RNA (**31**, 0.5 nmol) in a 10 mM Tris buffer containing 100 mM NaCl, 0.5 mM MgCl_2_, and 0.1 mM benzamide as an internal standard at pH 7.2, and the final volume of the solution was 100 μL. A solution of each one of the ODNs (**25, 26**) and 10 equiv of RNA (**31**) in the buffer was heated for 10 min at 95 °C, cooled to 0 °C at a rate of 0.5 °C/min, and then left at 37 °C for 10 min. A solution of 60 U/μL RNase H from *Escherichia coli* in the buffer was added to the solution to afford a final concentration of 50 U/mL and left at 37 °C for 30 min. RNase H was denatured by heating at 95 °C for 1 min, and then the mixture was cooled and analyzed by RP-HPLC. RP-HPLC was performed with a linear gradient of 0–11% MeCN over 44 min followed by 11–40% over 16 min in 0.1 M TEAA buffer (pH 7.0) at 50 °C with a flow rate of 0.5 mL/min.

## Supporting Information Available

Copies of ^1^H, ^13^C, ^31^P NMR spectra for new compounds, HPLC profiles, and results of thermal denaturation tests (PDF)
