# Supplementary material for: Solid-Phase Synthesis of Boranophosphate/Phosphorothioate/Phosphate Chimeric Oligonucleotides and Their Potential as Antisense Oligonucleotides
Source: J Org Chem. 2021 Dec 15;87(6):3895–909. doi: 10.1021/acs.joc.1c01812 (PMC8938928; doi:10.1021/acs.joc.1c01812)

Solid-Phase Synthesis of Boranophosphate/Phosphorothioate/Phosphate Chimeric Oligonucleotides and Their Potential as Antisense Oligonucleotides

Yuhei Takahashi[†], *Kazuki Sato*[†]*, *Takeshi Wada*[†]*

[†] Department of Medicinal and Life Science, Faculty of Pharmaceutical Sciences,

Tokyo University of Science, 2641 Yamazaki, Noda, Chiba 278-8510, Japan.

E-mail: kazuki_sato@rs.tus.ac.jp, twada@rs.tus.ac.jp

Support information

Table of Contents

RP-HPLC profiles of $T_{PS}T$ dimer (Figure S1)	S3
RP-HPLC profiles of $N_{PS}T$ dimers (Figure S2)	S5
RP-HPLC profiles of $N_{PB}T$ dimers (Figure S3)	S7
RP-HPLC profiles of tetramers (Figure S4)	S8
RP-HPLC profiles of dodecamers (Figures S5–S18)	S9
Thermal denaturation test (Figure S19–S20)	S19
Nuclease resistance (Figure S21)	S21
RNase H activity (25 U/mL, Figure S22)	S24
RNase H activity (50 U/mL, Figure S23)	S26
NMR spectra	S28

RP-HPLC profiles of T$_{PS}$T dimer (Table 1)

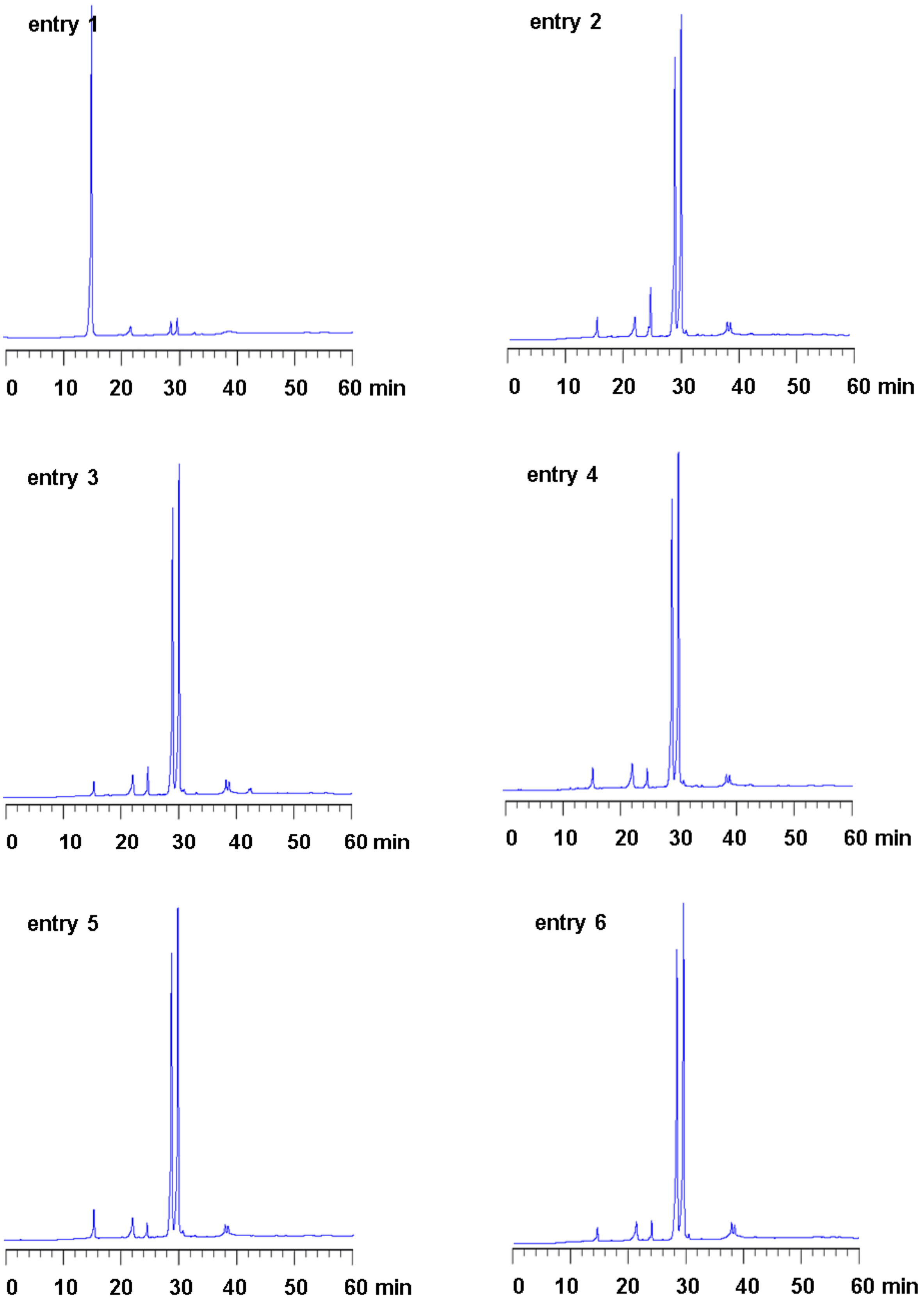

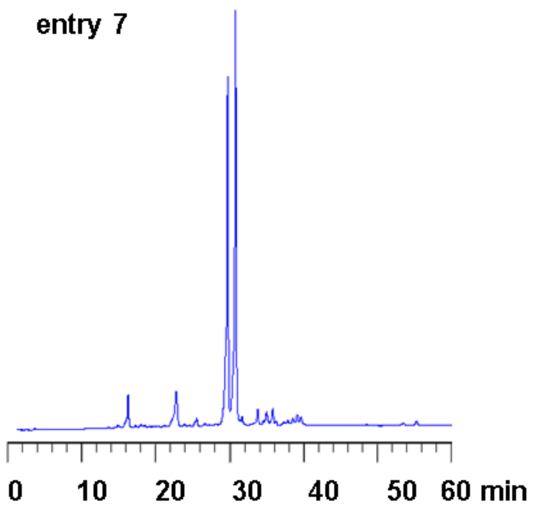

Figure S1. RP-HPLC profiles of crude $T_{PS}T$. RP-HPLC was performed with a linear gradient of 0%–30% acetonitrile for 60 min in 0.1 M triethylammonium acetate buffer (pH 7.0) at 30 °C at a flow rate of 0.5 mL/min using a C18 column.

RP-HPLC profiles of $B_{PS}T$ dimers (Table 2)

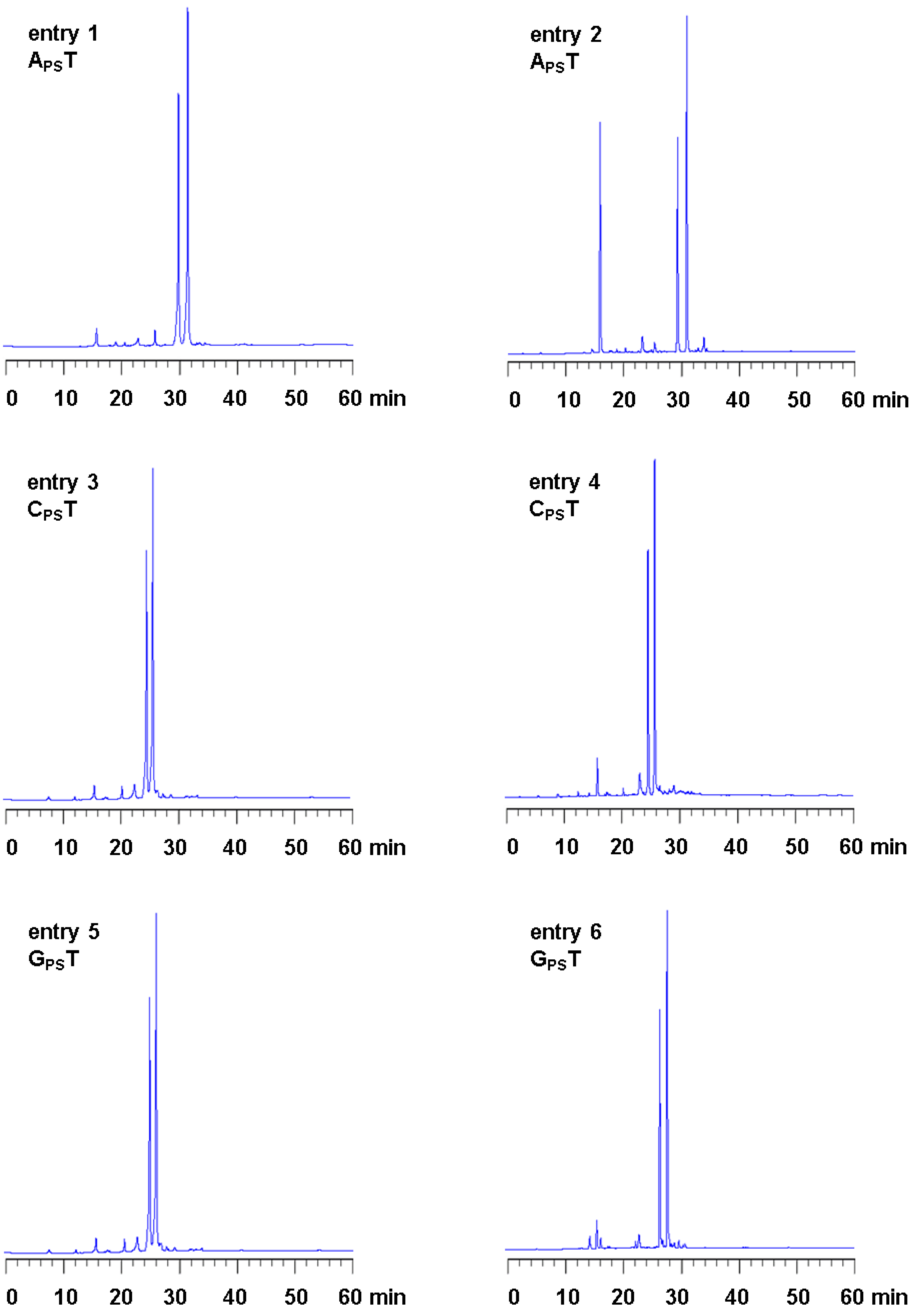

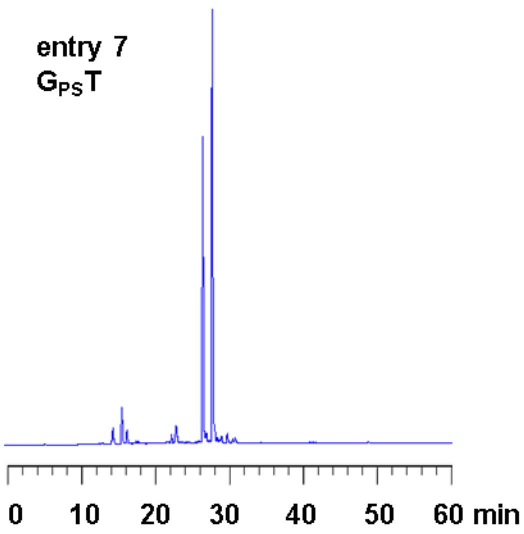

Figure S2. RP-HPLC profiles of crude $B_{PS}T$. RP-HPLC was performed with a linear gradient of 0%–30% acetonitrile for 60 min in 0.1 M triethylammonium acetate buffer (pH 7.0) at 30 °C at a flow rate of 0.5 mL/min using a C18 column.

RP-HPLC profiles of $\underline{B}_{PB}T$ dimers (Table 3)

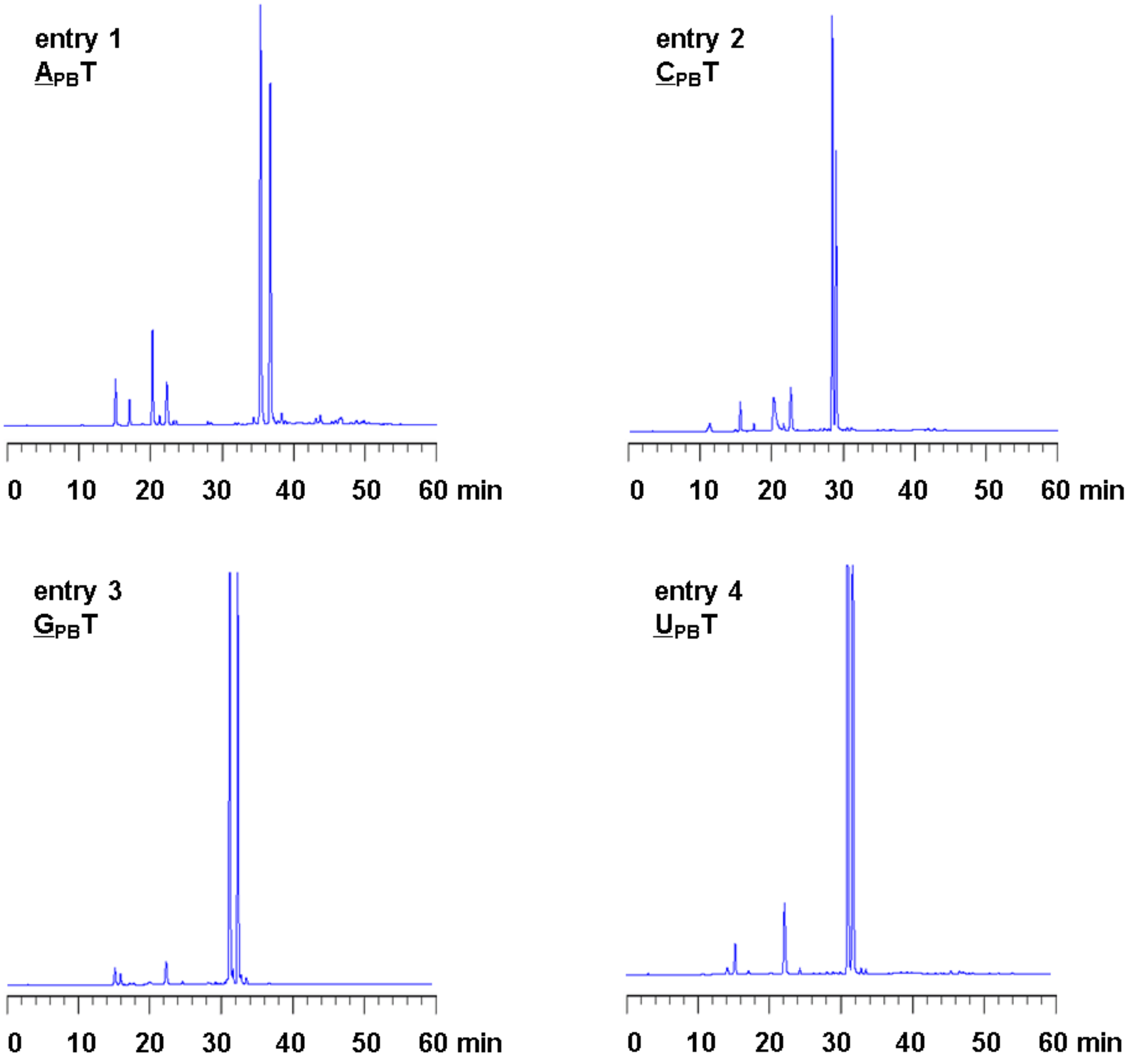

Figure S3. RP-HPLC profiles of crude $\underline{B}_{PB}T$. RP-HPLC was performed with a linear gradient of 0%–30% acetonitrile for 60 min in 0.1 M triethylammonium acetate buffer (pH 7.0) at 30 °C at a flow rate of 0.5 mL/min using a C18 column.

RP-HPLC profiles of tetramers (Table 4, entries 1 and 2)

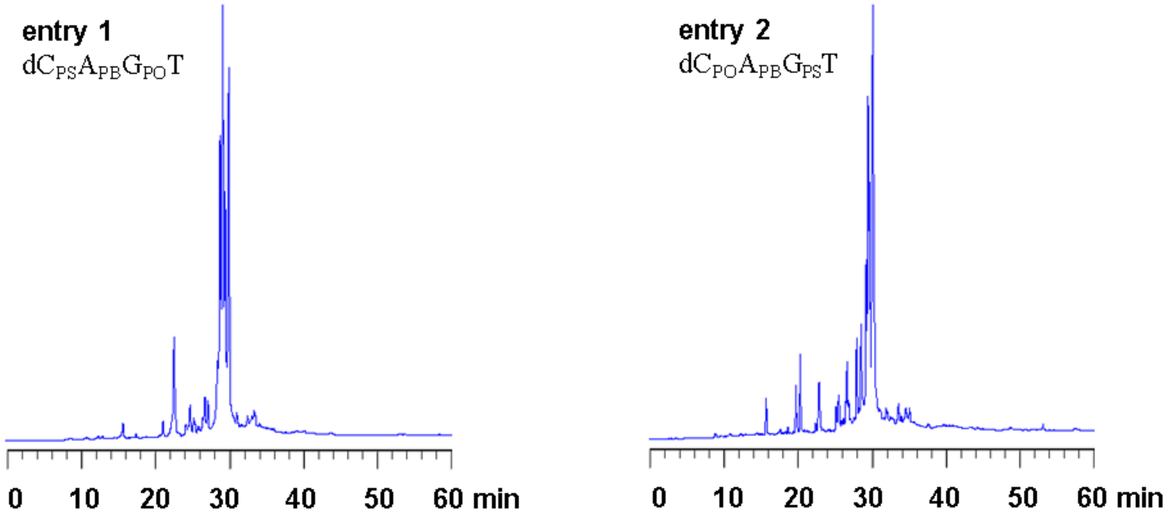

Figure S4. RP-HPLC profiles of crude tetramers (dC$_{PS}$A$_{PB}$G$_{PO}$T and dC$_{PO}$A$_{PB}$G$_{PS}$T). RP-HPLC was performed with a linear gradient of 0%–30% acetonitrile for 60 min in 0.1 M triethylammonium acetate buffer (pH 7.0) at 30 °C at a flow rate of 0.5 mL/min using a C18 column.

HPLC profiles of dodecamers (Table 4 entry 3)

crude

d$C_{PS}A_{PS}G_{PS}T_{PS}C_{PB}A_{PB}G_{PB}T_{PB}C_{PO}A_{PO}G_{PO}T$

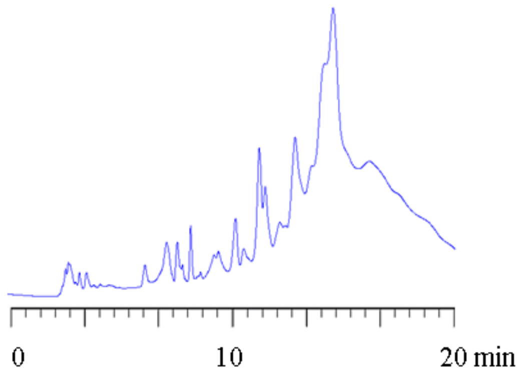

Figure S5. RP-HPLC profile of crude d$C_{PS}A_{PS}G_{PS}T_{PS}C_{PB}A_{PB}G_{PB}T_{PB}C_{PO}A_{PO}G_{PO}T$. RP-HPLC was performed with a linear gradient of 5%–40% MeOH in 100 mM HFIP, 8 mM TEA for 20 min at 60 °C at a flow rate of 0.5 mL/min using a C18 column.

pure

d$C_{PS}A_{PS}G_{PS}T_{PS}C_{PB}A_{PB}G_{PB}T_{PB}C_{PO}A_{PO}G_{PO}T$

RP-HPLC

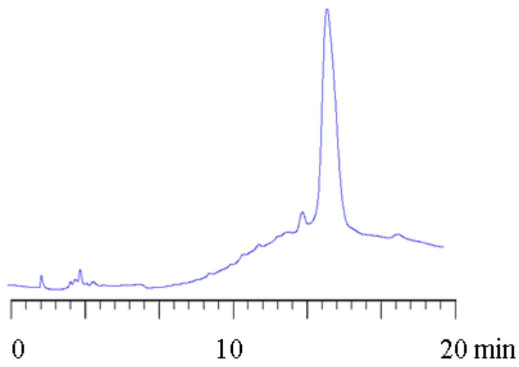

Figure S6. RP-HPLC profile of pure d$C_{PS}A_{PS}G_{PS}T_{PS}C_{PB}A_{PB}G_{PB}T_{PB}C_{PO}A_{PO}G_{PO}T$. RP-HPLC was performed with a linear gradient of 5%–40% MeOH in 100 mM HFIP, 8 mM TEA for 20 min at 60 °C at a flow rate of 0.5 mL/min using a C18 column.

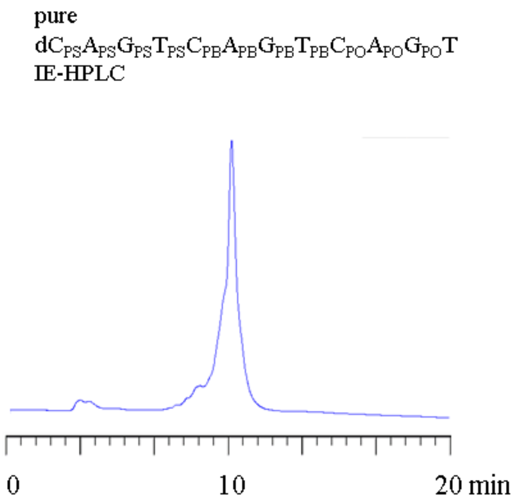

Figure S7. IE-HPLC profile of pure $dC_{PS}A_{PS}G_{PS}T_{PS}C_{PB}A_{PB}G_{PB}T_{PB}C_{PO}A_{PO}G_{PO}T$. IE-HPLC was performed with a linear gradient of 0 M–1M $NaClO_4$ in 50%–25% CH_3CN, 10 mM Tris-HCl (pH 7.5) for 20 min at 30 °C with a flow rate of 0.4 mL/min using a quaternary ammonium anion exchange resin column.

HPLC profiles of dodecamers (Table 4 entry 4)

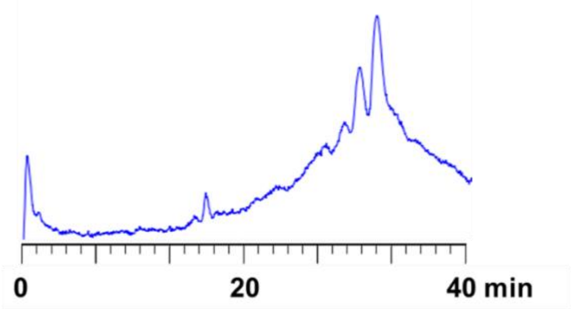

Figure S8. IE-HPLC profile of crude $dG_{PB}C_{PS}A_{PB}T_{PO}T_{PO}G_{PO}G_{PO}T_{PS}A_{PB}T_{PS}T_{PB}C$. IE-HPLC was performed with a linear gradient of 0 M–1 M NaCl in 30% *i*PrOH, 10 mM Tris-HCl (pH 7.5) for 40 min at a flow rate of 0.4 mL/min using a quaternary ammonium anion exchange resin column.

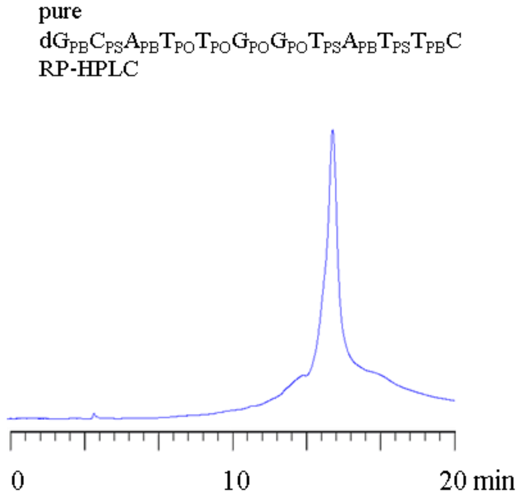

Figure S9. RP-HPLC profile of pure $dG_{PB}C_{PS}A_{PB}T_{PO}T_{PO}G_{PO}G_{PO}T_{PS}A_{PB}T_{PS}T_{PB}C$. RP-HPLC was performed with a linear gradient of 5%–40% MeOH in 100 mM HFIP, 8 mM TEA for 20 min at 60 °C at a flow rate of 0.5 mL/min using a C18 column.

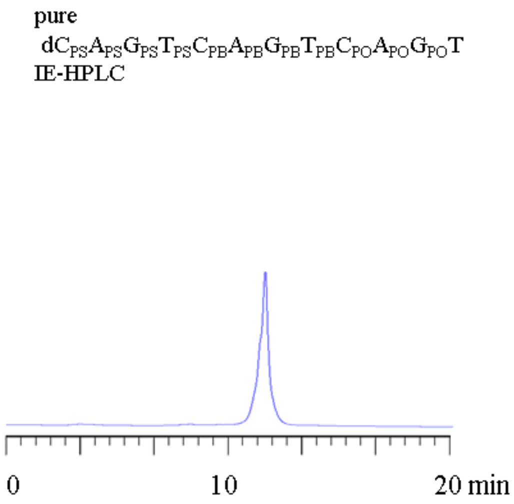

Figure S9. IE-HPLC profile of pure d$G_{PB}C_{PS}A_{PB}T_{PO}T_{PO}G_{PO}G_{PO}T_{PS}A_{PB}T_{PS}T_{PB}C$. IE-HPLC was performed with a linear gradient of 0 M–1 M $NaClO_4$ in 50%–25% CH_3CN, 10 mM Tris-HCl (pH 7.5) for 20 min at 30 °C with a flow rate of 0.4 mL/min using a quaternary ammonium anion exchange resin column.

HPLC profiles of dodecamers (Table 4 entry 5)

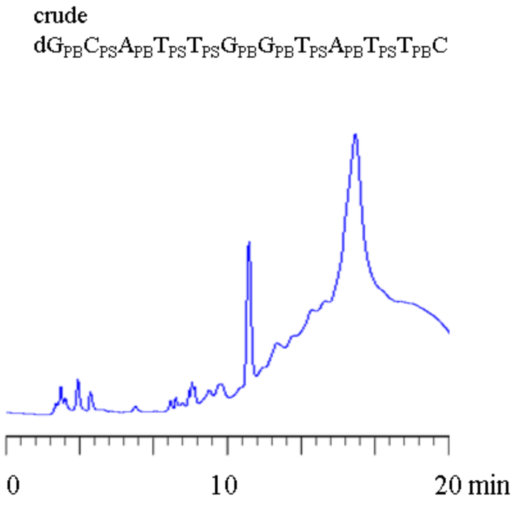

Figure S10. RP-HPLC profile of crude $dG_{PB}C_{PS}A_{PB}T_{PS}T_{PS}G_{PB}G_{PB}T_{PS}A_{PB}T_{PS}T_{PB}C$. RP-HPLC was performed with a linear gradient of 5%–40% MeOH in 100 mM HFIP, 8 mM TEA for 20 min at 60 °C at a flow rate of 0.5 mL/min using a C18 column.

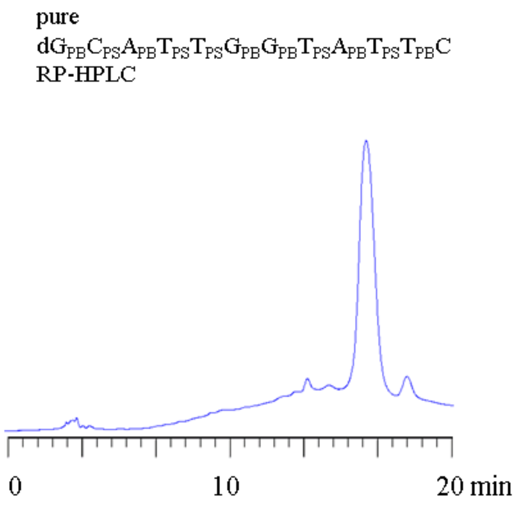

Figure S11. RP-HPLC profile of pure $dG_{PB}C_{PS}A_{PB}T_{PS}T_{PS}G_{PB}G_{PB}T_{PS}A_{PB}T_{PS}T_{PB}C$. RP-HPLC was performed with a linear gradient of 5%–40% MeOH in 100 mM HFIP, 8 mM TEA for 20 min at 60 °C at a flow rate of 0.5 mL/min using a C18 column.

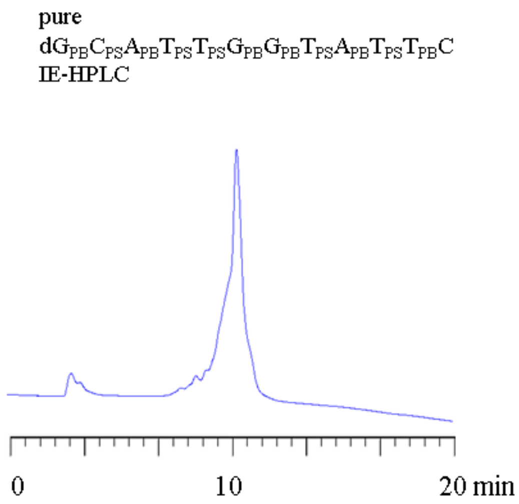

Figure S12. IE-HPLC profile of pure dG$_{PB}$C$_{PS}$A$_{PB}$T$_{PS}$T$_{PS}$G$_{PB}$G$_{PB}$T$_{PS}$A$_{PB}$T$_{PS}$T$_{PB}$C. IE-HPLC was performed with a linear gradient of 0 M–1 M $NaClO_4$ in 50%–25% CH_3CN, 10 mM Tris-HCl (pH 7.5) for 20 min at 30 °C with a flow rate of 0.4 mL/min using a quaternary ammonium anion exchange resin column.

HPLC profiles of dodecamers (Table 4 entry 6)

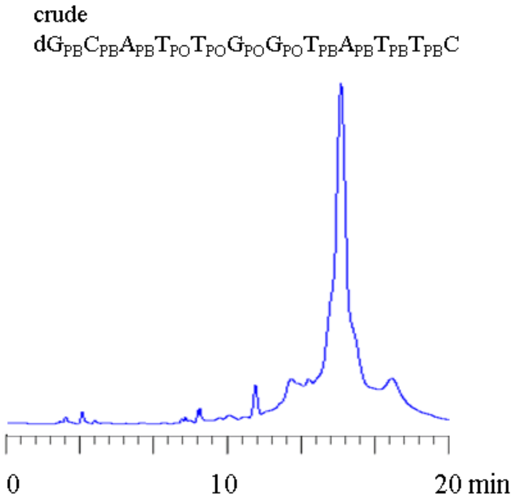

Figure S13. RP-HPLC profile of crude $dG_{PB}C_{PB}A_{PB}T_{PO}T_{PO}G_{PO}G_{PO}T_{PB}A_{PB}T_{PB}T_{PB}C$. RP-HPLC was performed with a linear gradient of 5%–40% MeOH in 100 mM HFIP, 8 mM TEA for 20 min at 60 °C at a flow rate of 0.5 mL/min using a C18 column.

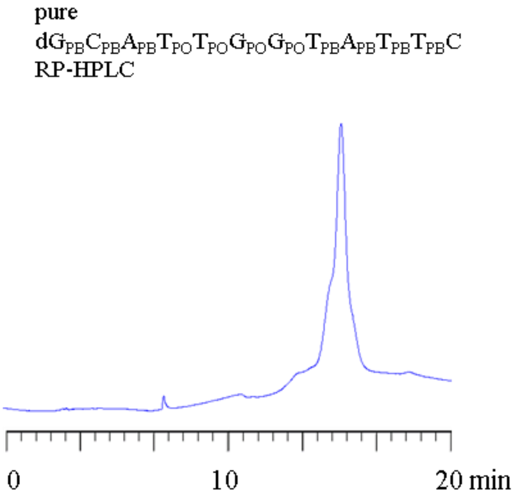

Figure S14. RP-HPLC profile of pure $dG_{PB}C_{PB}A_{PB}T_{PO}T_{PO}G_{PO}G_{PO}T_{PB}A_{PB}T_{PB}T_{PB}C$. RP-HPLC was performed with a linear gradient of 5%–40% MeOH in 100 mM HFIP, 8 mM TEA for 20 min at 60 °C at a flow rate of 0.5 mL/min using a C18 column.

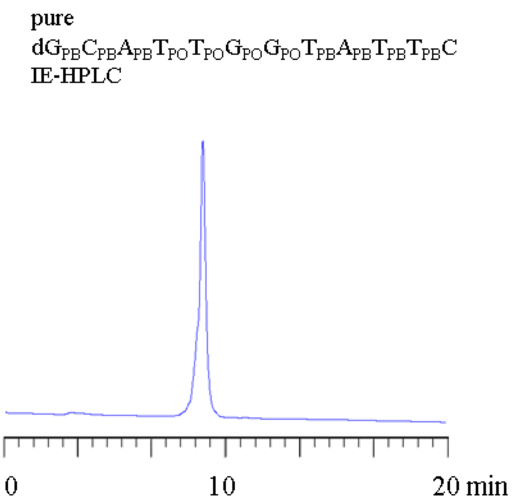

Figure S15. IE-HPLC profile of pure $dG_{PB}C_{PB}A_{PB}T_{PO}T_{PO}G_{PO}G_{PO}T_{PB}A_{PB}T_{PB}T_{PB}C$. IE-HPLC was performed with a linear gradient of 0 M–1 M $NaClO_4$ in 50%–25% CH_3CN, 10 mM Tris-HCl (pH 7.5) for 20 min at 30 °C with a flow rate of 0.4 mL/min using a quaternary ammonium anion exchange resin column.

HPLC profiles of dodecamers (Table 4 entry 7)

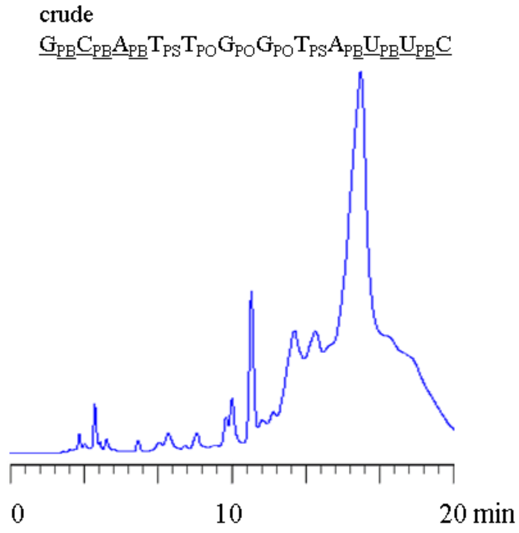

Figure S16. RP-HPLC profile of crude $\underline{G}_{PB}\underline{C}_{PB}\underline{A}_{PB}T_{PS}T_{PO}G_{PO}G_{PO}T_{PS}A_{PB}\underline{U}_{PB}\underline{U}_{PB}\underline{C}$. RP-HPLC was performed with a linear gradient of 5%–40% MeOH in 100 mM HFIP, 8 mM TEA for 20 min at 60 °C at a flow rate of 0.5 mL/min using a C18 column.

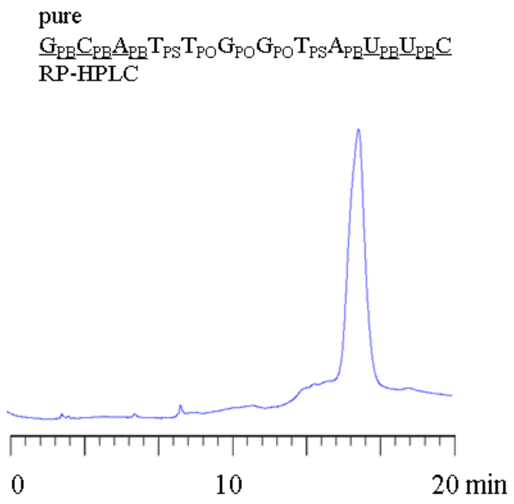

Figure S17. RP-HPLC profile of pure $\underline{G}_{PB}\underline{C}_{PB}\underline{A}_{PB}T_{PS}T_{PO}G_{PO}G_{PO}T_{PS}A_{PB}\underline{U}_{PB}\underline{U}_{PB}\underline{C}$. RP-HPLC was performed with a linear gradient of 5%–40% MeOH in 100 mM HFIP, 8 mM TEA for 20 min at 60 °C at a flow rate of 0.5 mL/min using a C18 column.

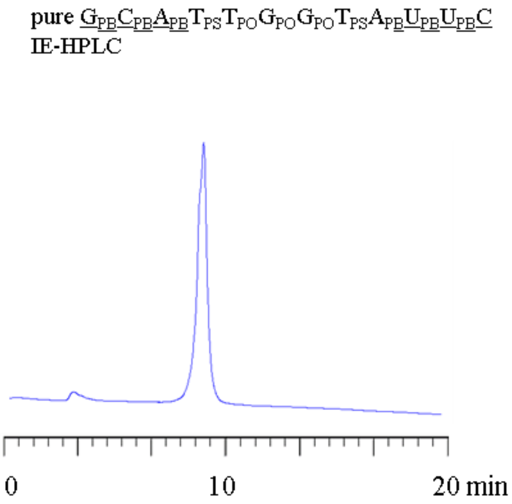

Figure S18. IE-HPLC profile of pure $\underline{G}_{PB}\underline{C}_{PB}\underline{A}_{PB}T_{PS}T_{PO}G_{PO}G_{PO}T_{PS}A_{PB}\underline{U}_{PB}\underline{U}_{PB}\underline{C}$. IE-HPLC was performed with a linear gradient of 0 M–1 M $NaClO_4$ in 50%–25% CH_3CN, 10 mM Tris-HCl (pH 7.5) for 20 min at 30 °C with a flow rate of 0.4 mL/min using a quaternary ammonium anion exchange resin column.

Thermal denaturation test

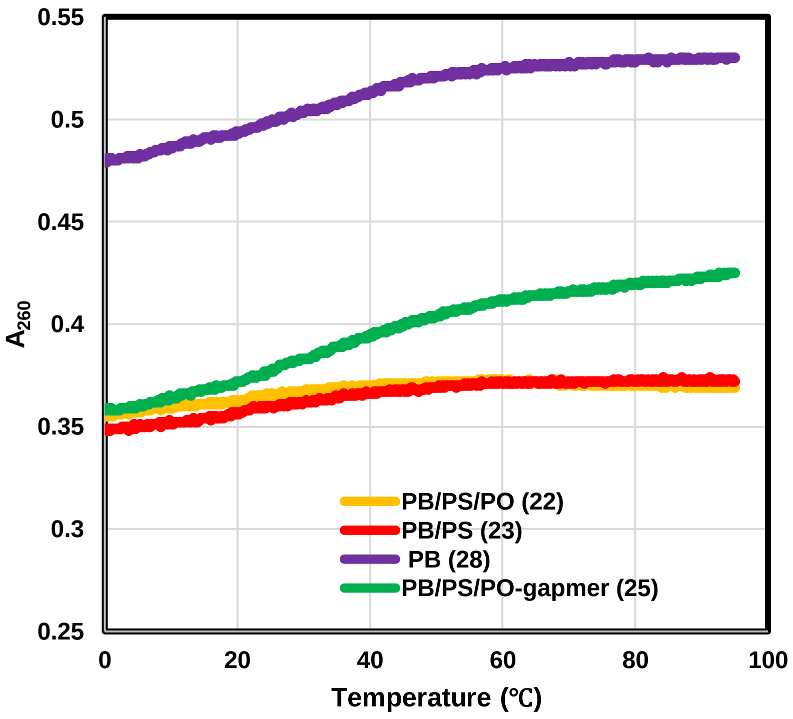

Figure S19. UV Melting curves of single stranded ODNs **22**, **23**, **25**, and **28**.

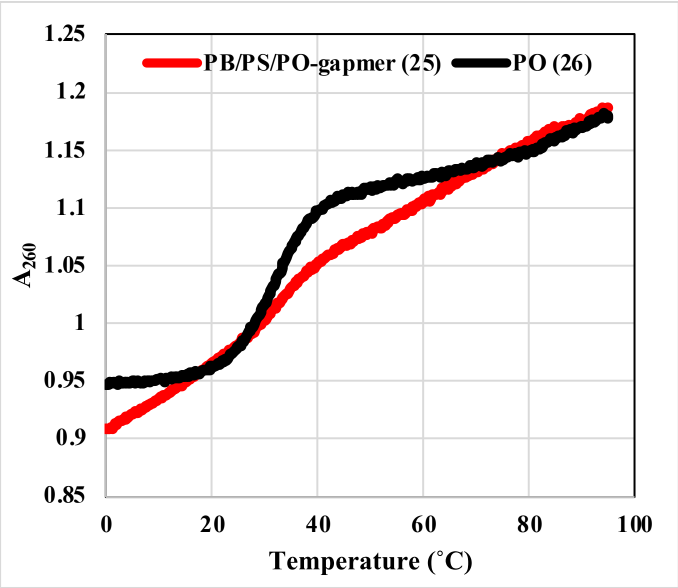

Figure S20. UV Melting curves of double stranded ODNs **25** and **26** with cRNA with a mismatched base.

Nuclease resistance

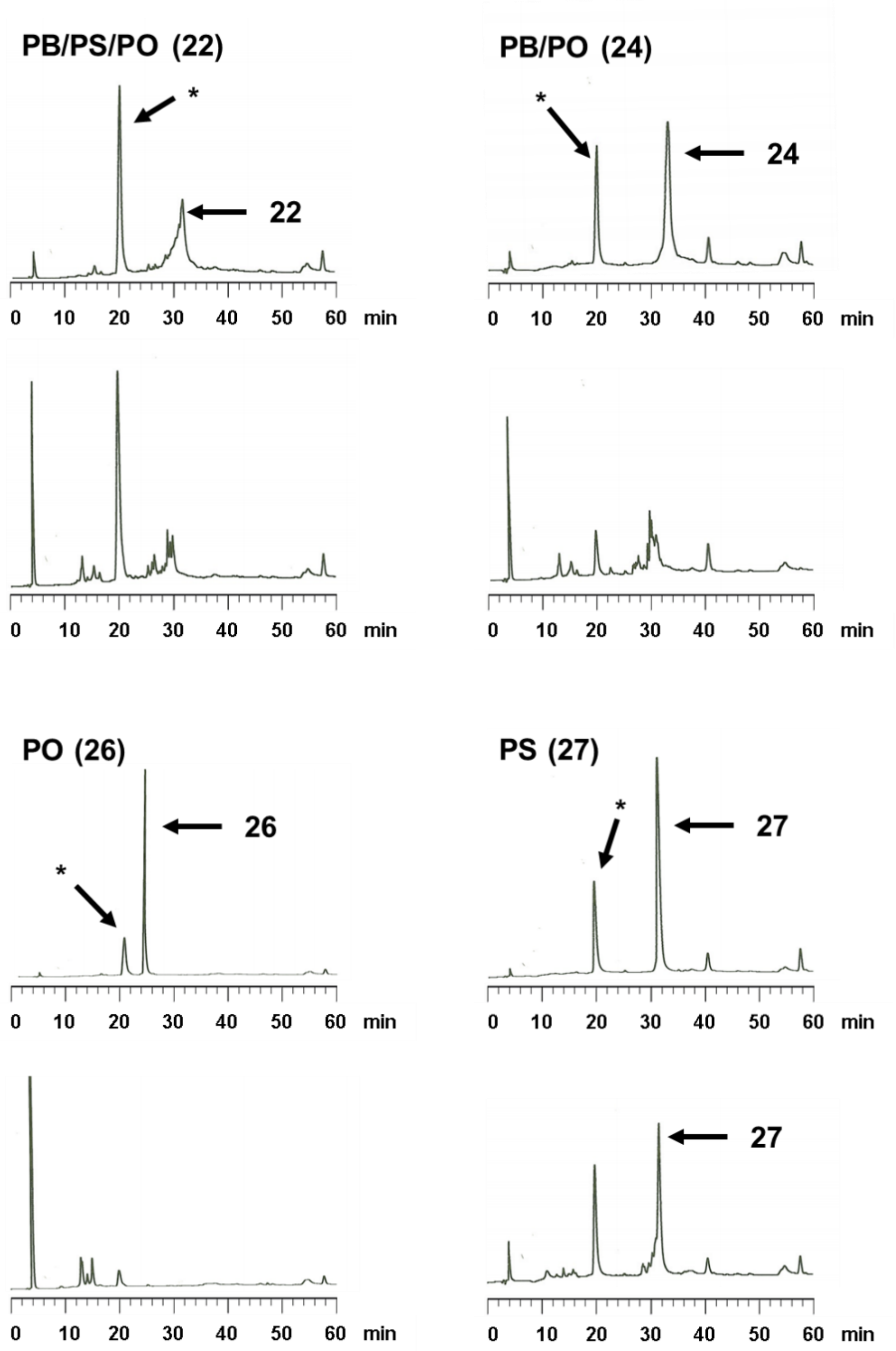

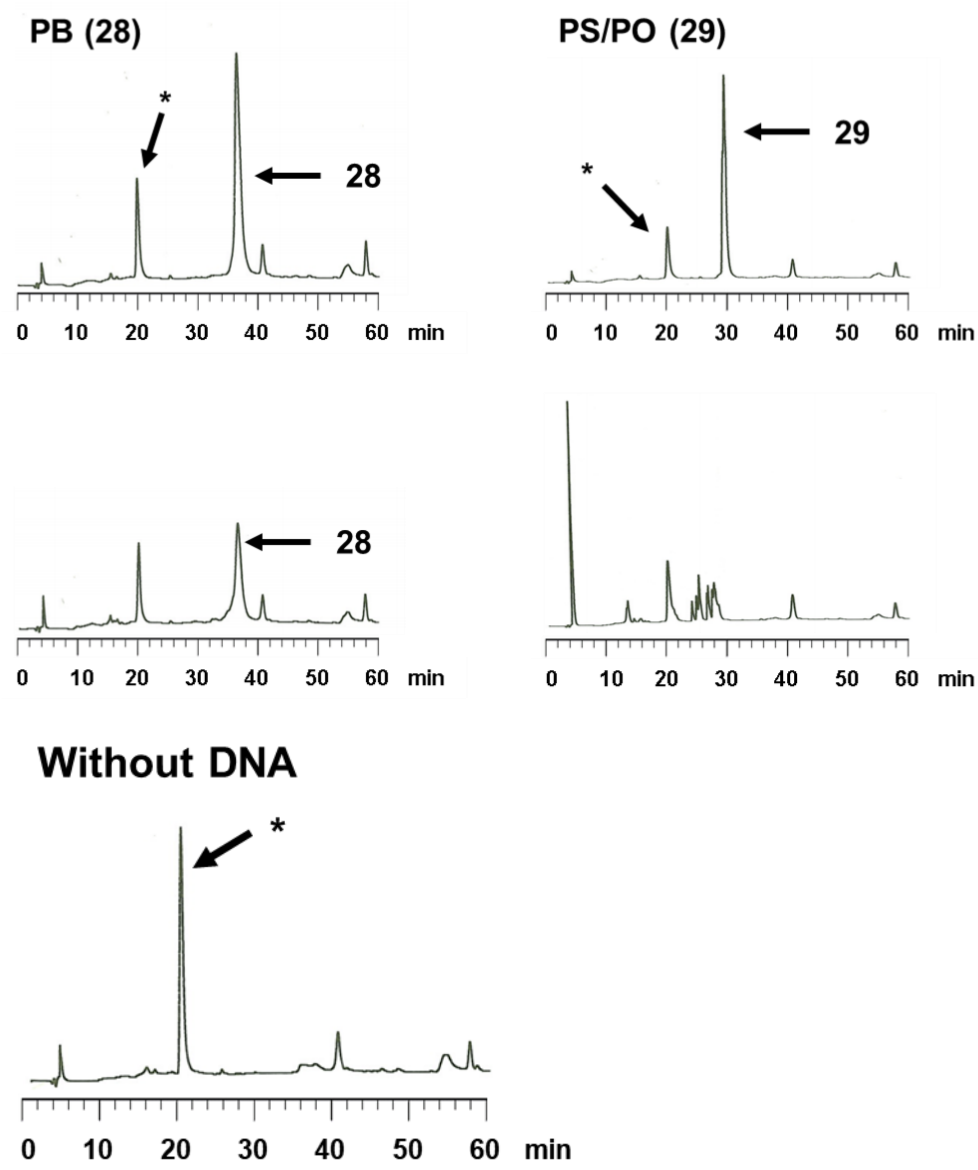

* indicates an artifact peak

Figure S21. RP-HPLC profiles of ODN before (upper) and after (lower) the treatment with snake venom phosphodiesterase (SVPDE) for 12 h at 37°C. RP-HPLC was performed with a linear gradient of 0%–40% acetonitrile for 60 min in 0.1 M triethylammonium acetate buffer (pH 7.0) at 30 °C at a flow rate of 0.5 mL/min using a C18 column

RNase H activity (25 U/mL)

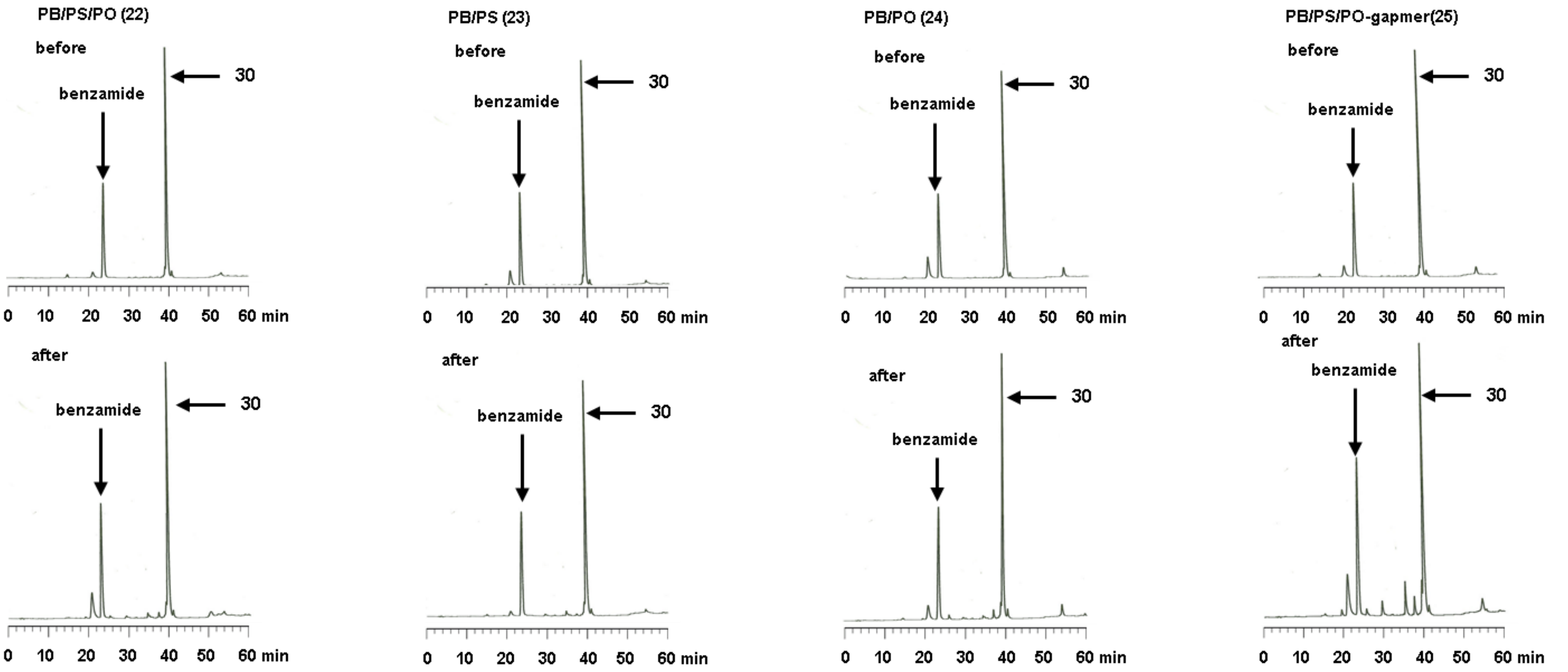

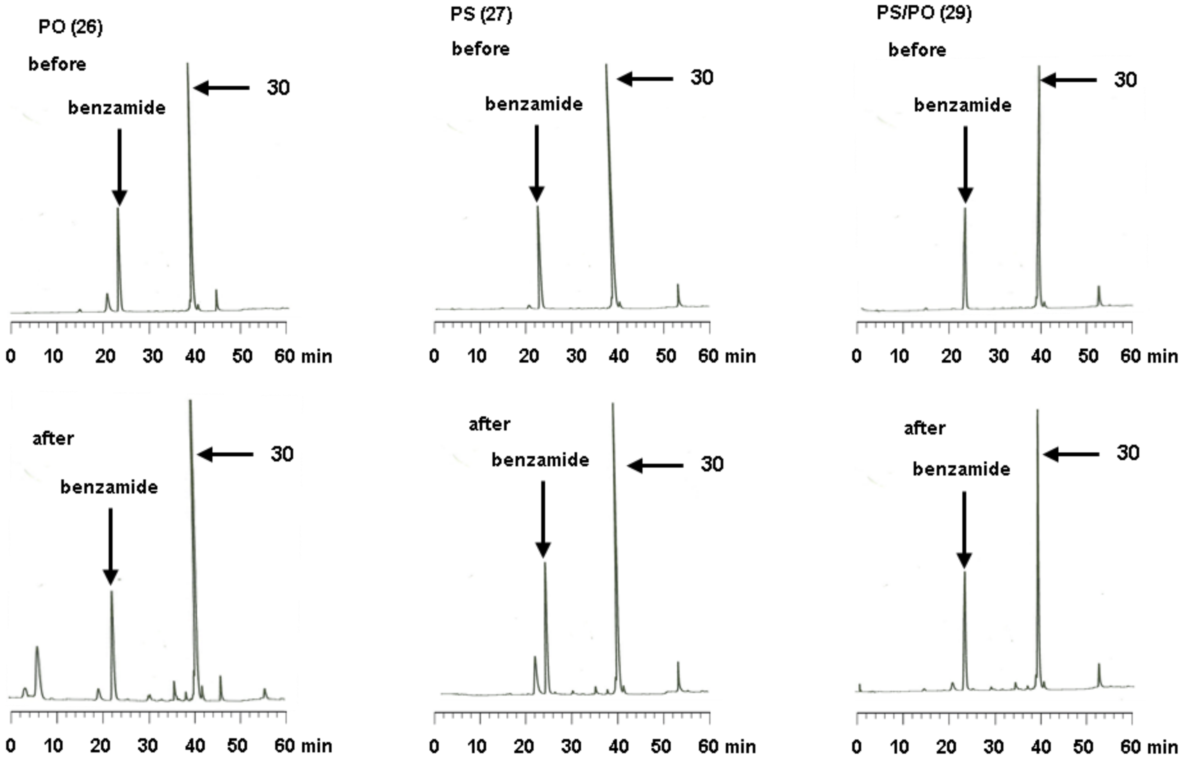

Figure S22. RP-HPLC profiles of ODN/cRNA duplexes before (upper) and after (lower) the treatment with 25 U/mL RNase H for 30 min at 37 °C RP-HPLC was performed with a linear gradient of 0%−11% MeCN over 44 min followed by 11−40% over 16 min in 0.1 M TEAA buffer (pH 7.0) at 50 °C with a flow rate of 0.5 mL/min.

RNase H activity (50 U/mL)

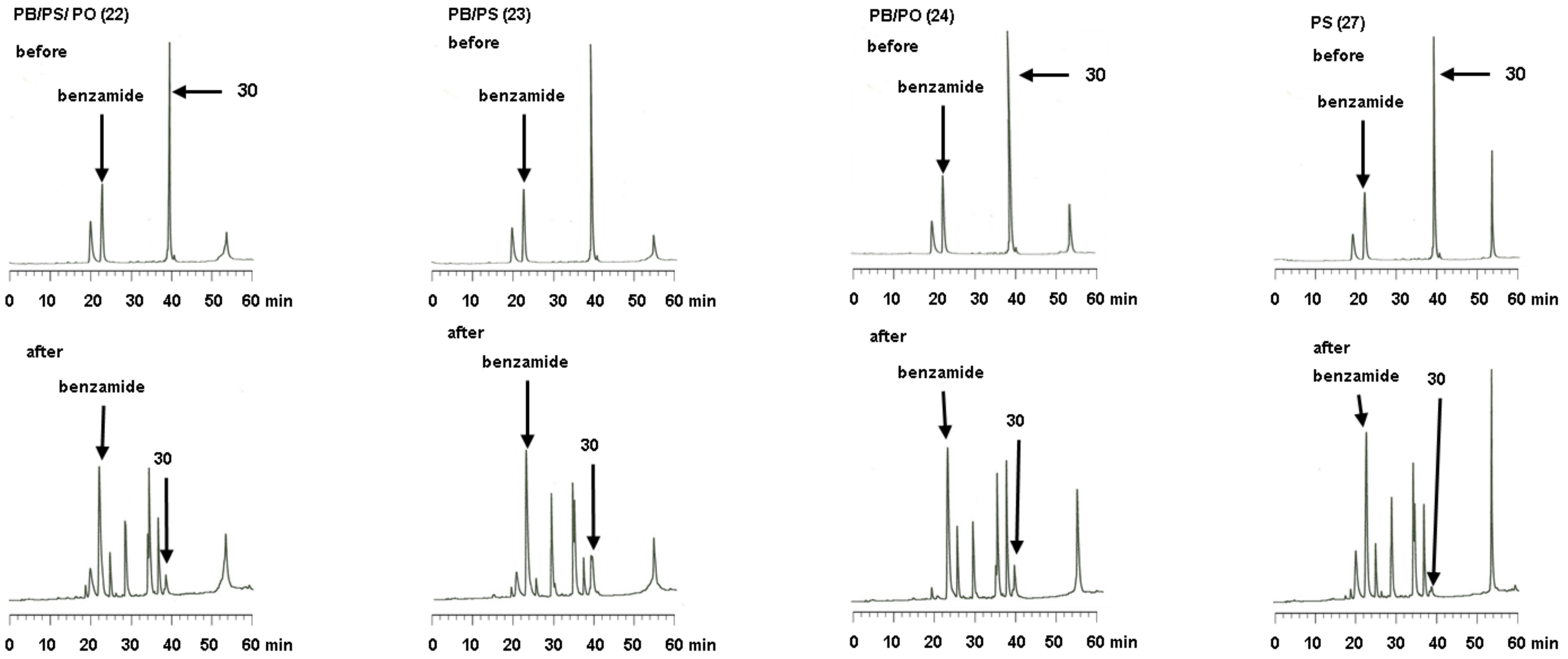

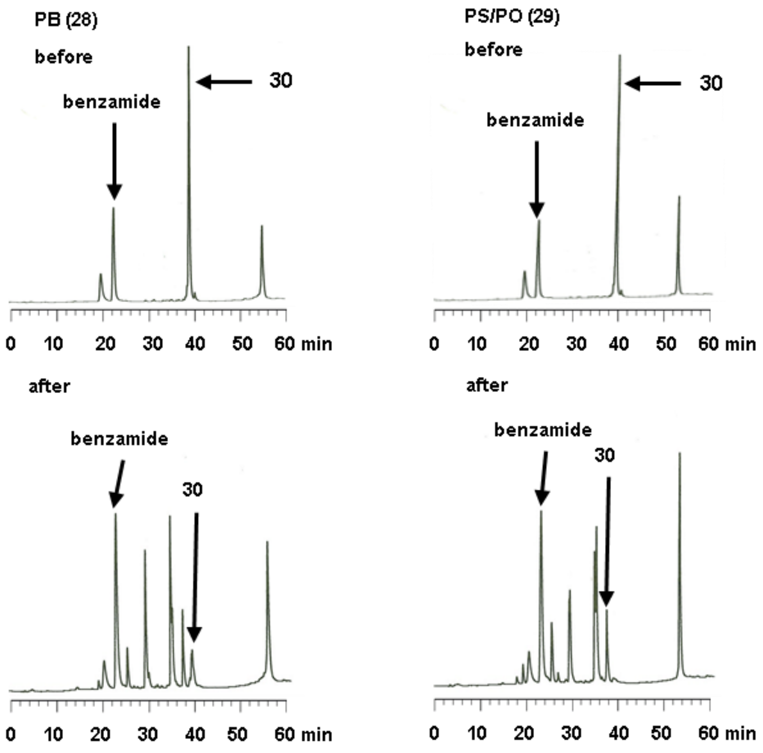

Figure S23. RP-HPLC profiles of ODN/cRNA duplexes before (upper) and after (lower) the treatment with 50 U/mL RNase H for 30 min at 37 °C RP-HPLC was performed with a linear gradient of 0%−11% MeCN over 44 min followed by 11%−40% over 16 min in 0.1 M TEAA buffer (pH 7.0) at 50 °C with a flow rate of 0.5 mL/min.

^{1}H, ^{13}C, ^{31}P NMR spectra

NMR spectra

^{1}H-NMR (400 MHz, $CDCl_3$)

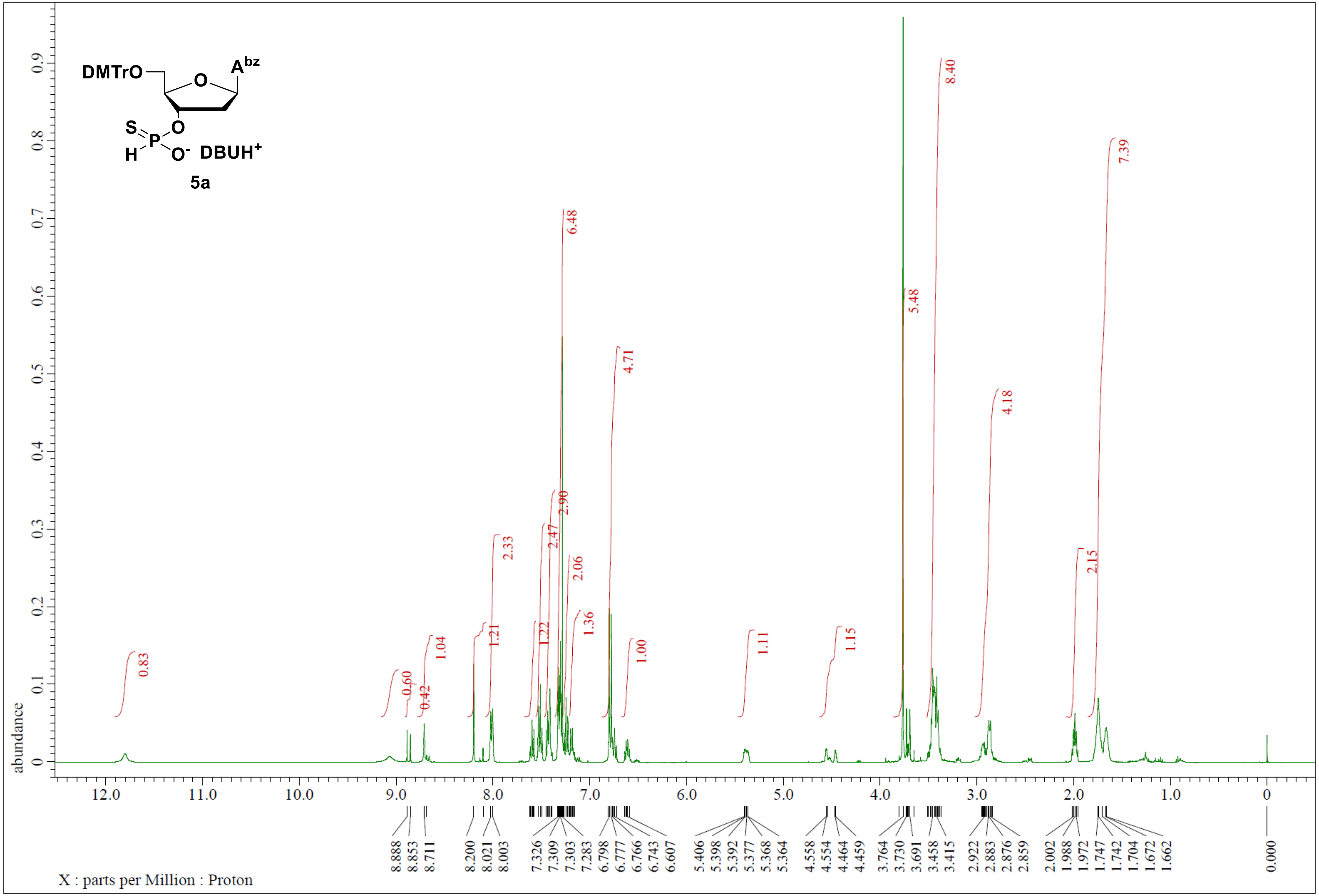

$^{13}C\{H\}$ NMR (100 MHz, $CDCl_3$)

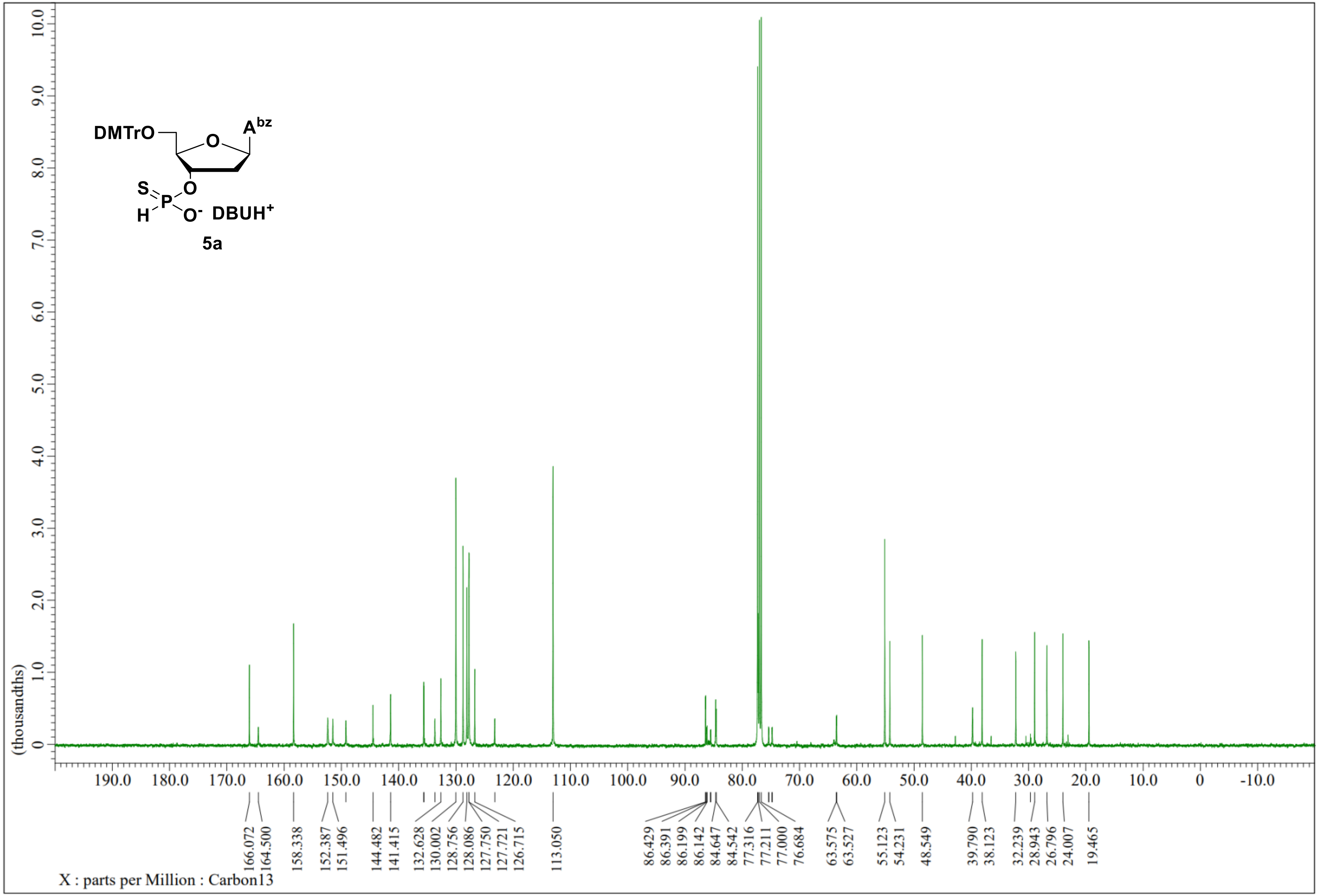

$^{31}P\{^{1}H\}$ NMR (162 MHz, $CDCl_3$)

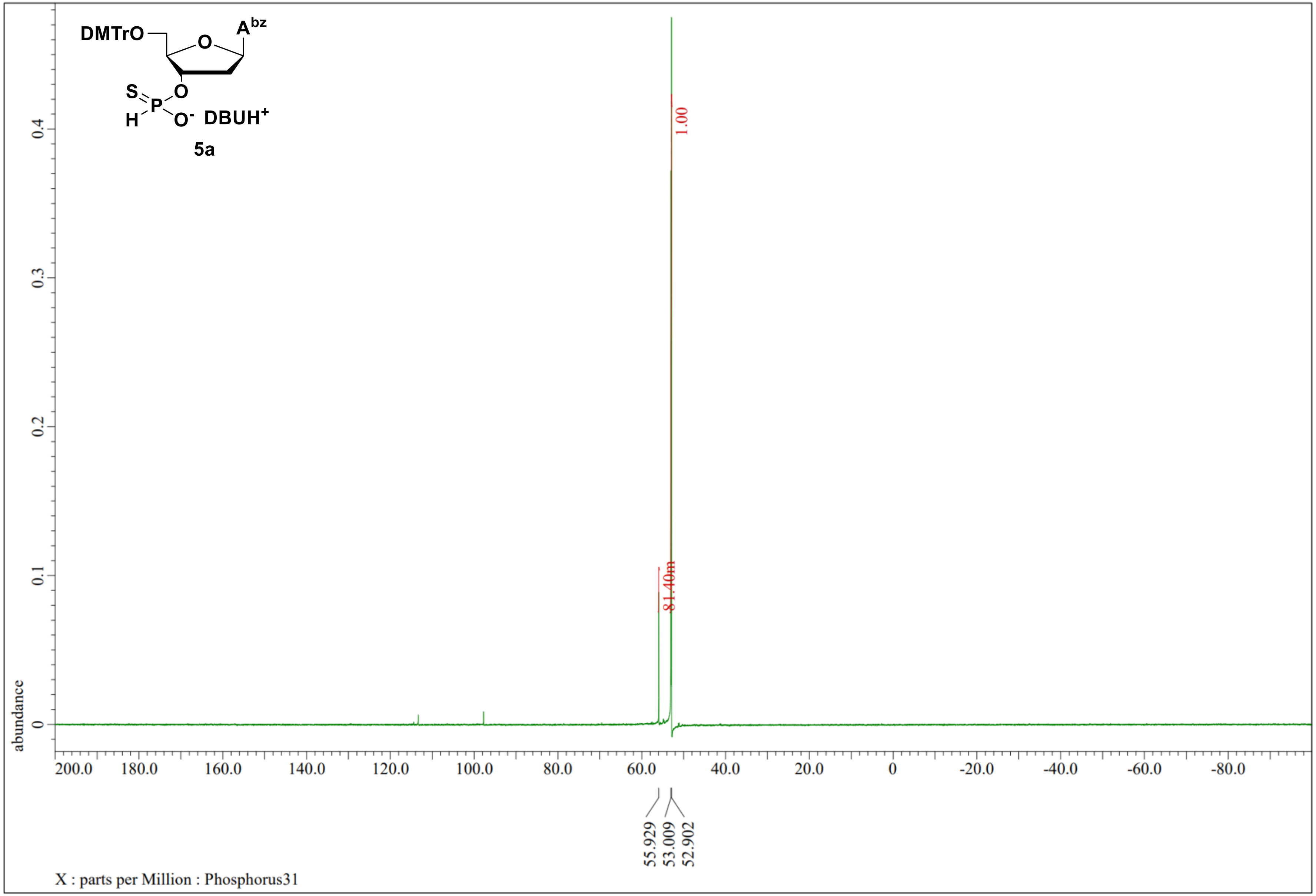

^{1}H-NMR (400 MHz, $CDCl_3$)

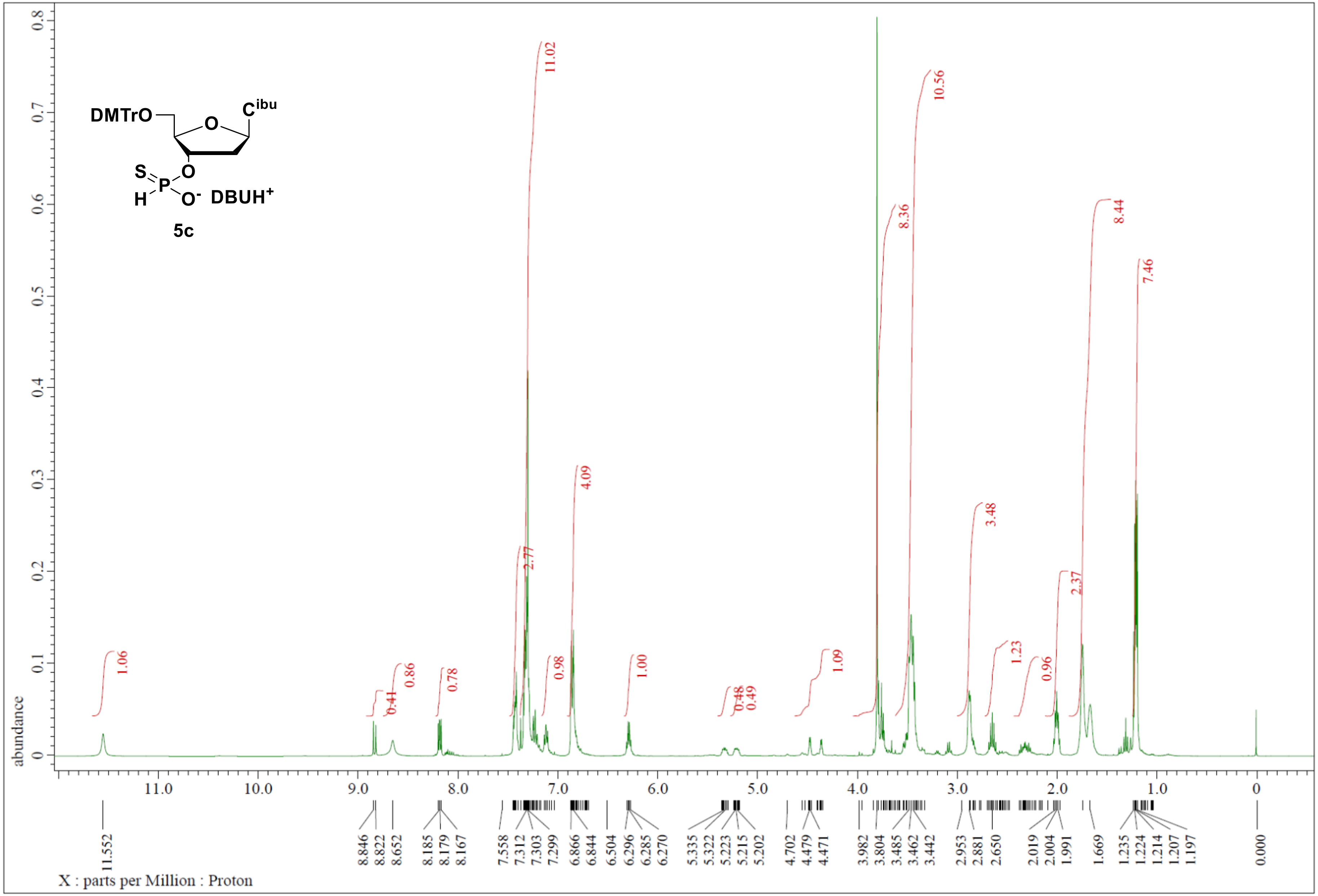

$^{13}C\{^{1}H\}$ NMR (100 MHz, $CDCl_3$)

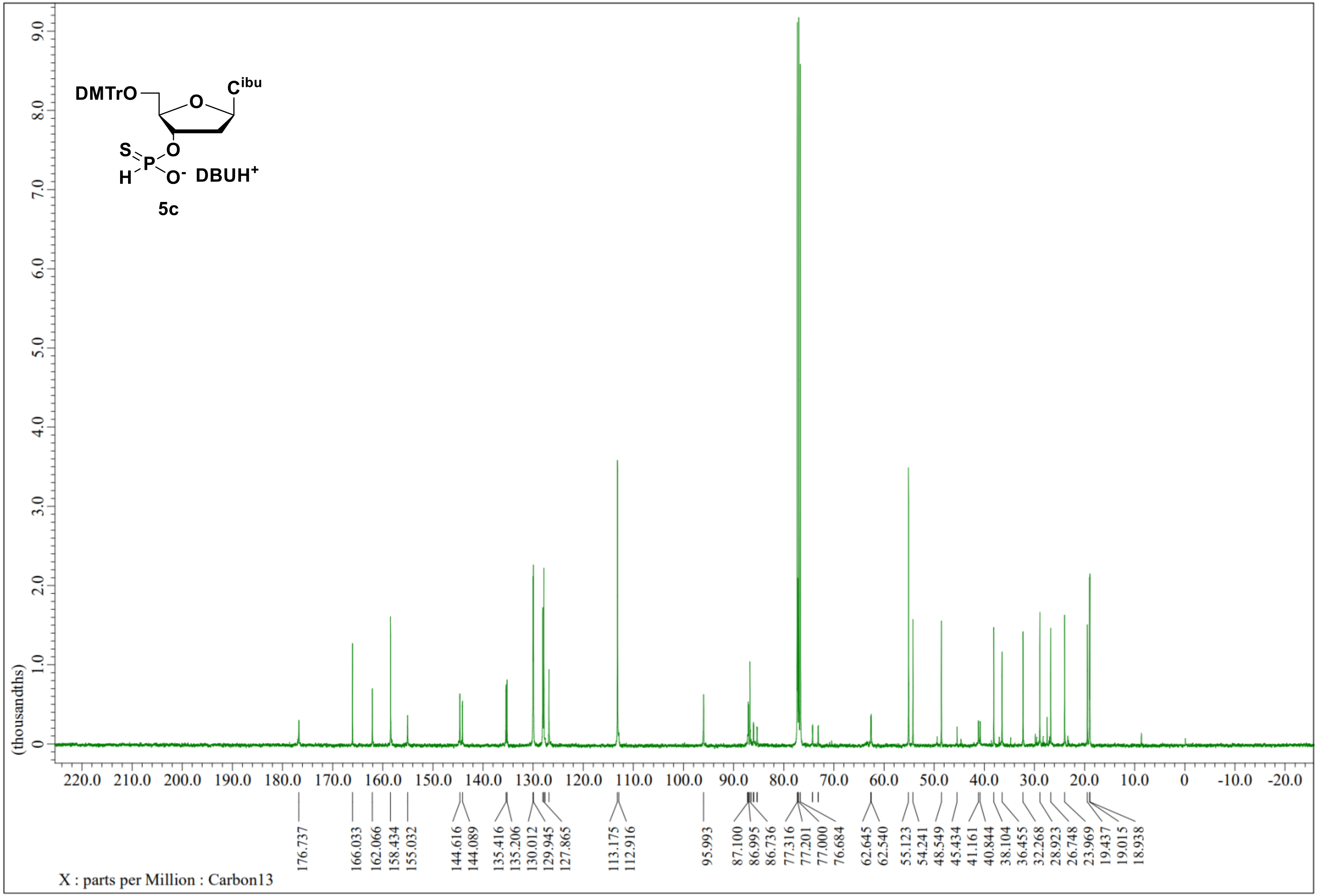

$^{31}P\{^{1}H\}$ NMR (162 MHz, $CDCl_3$)

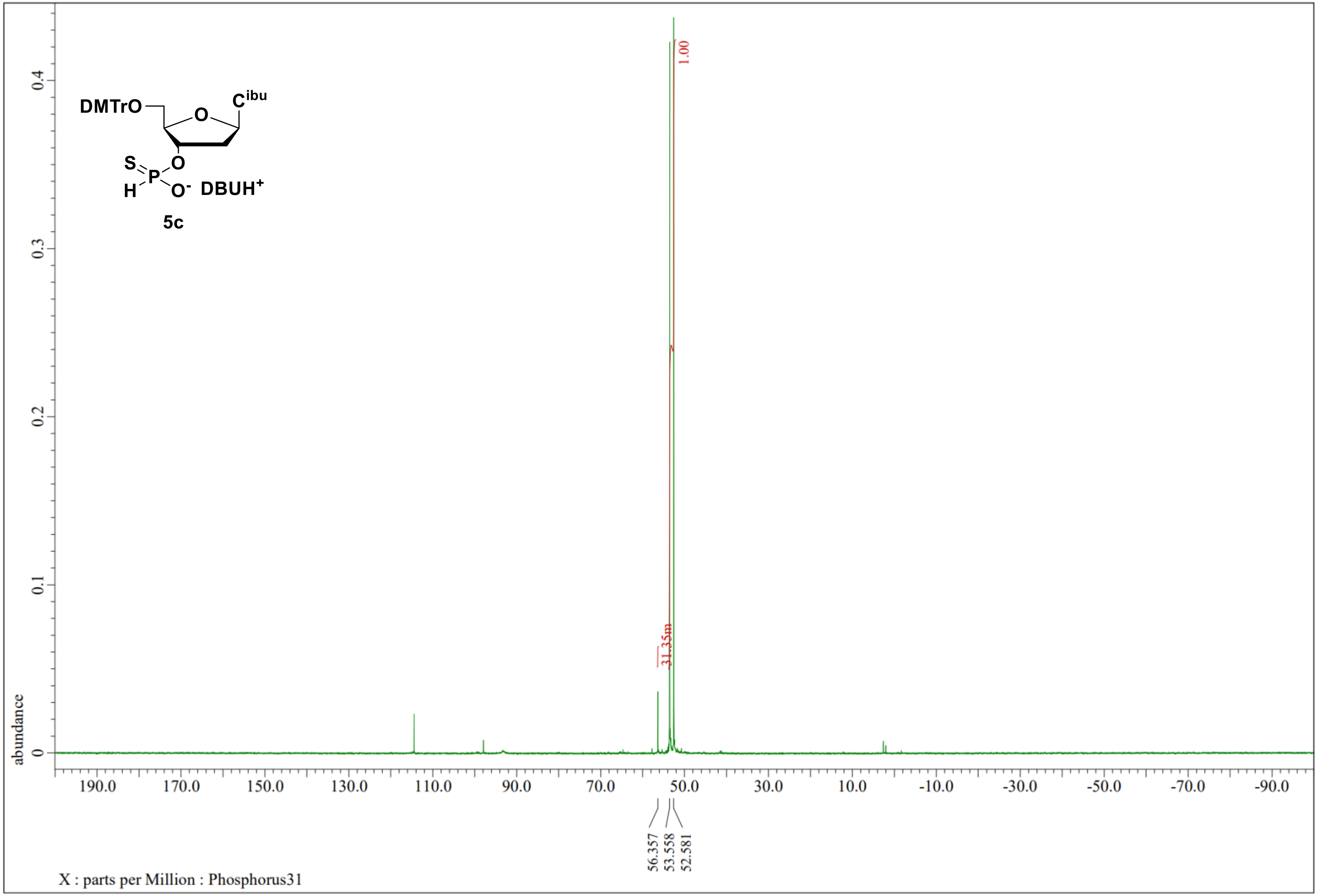

^{1}H-NMR (400 MHz, $CDCl_3$)

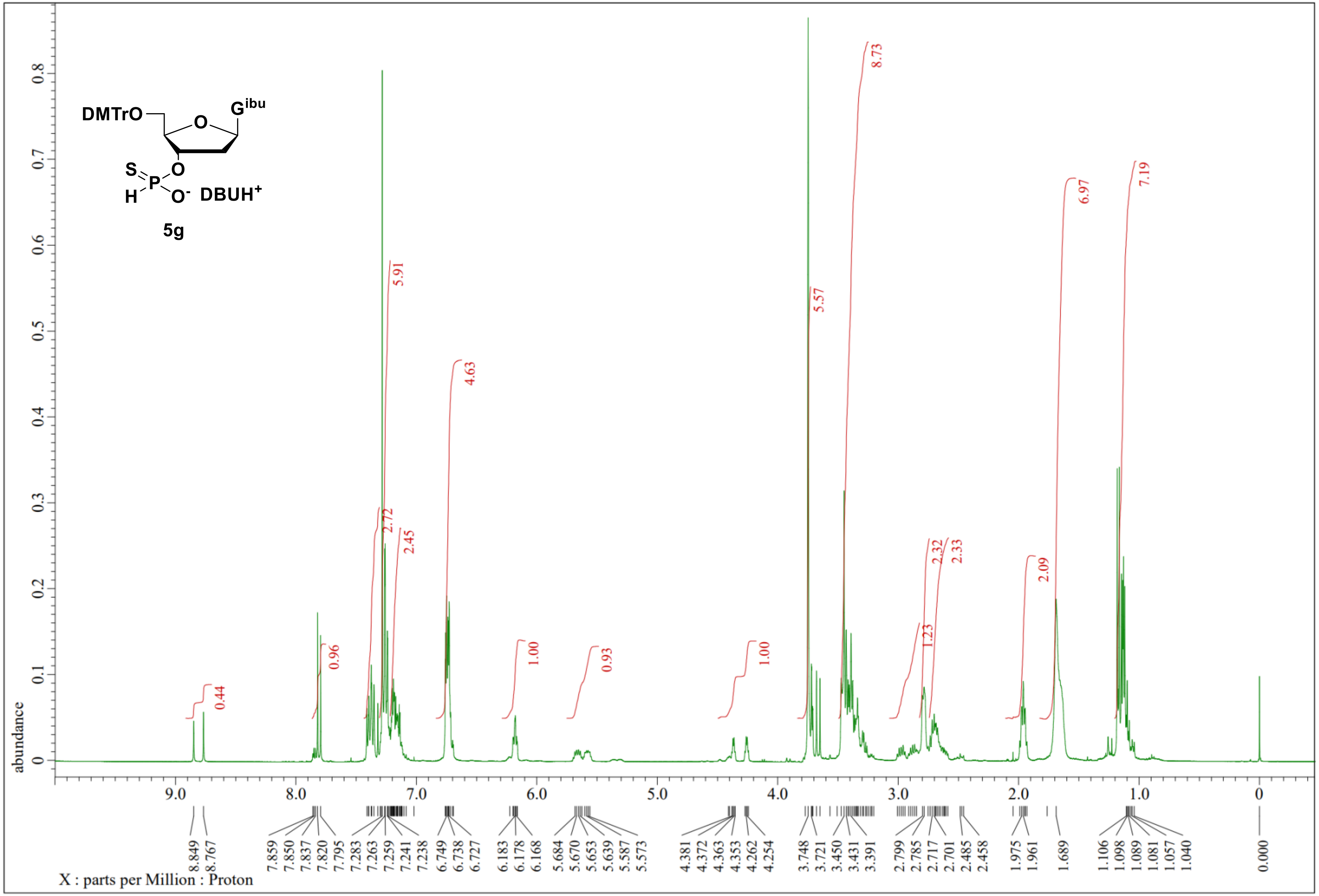

$^{13}C\{H\}$ NMR (100 MHz, $CDCl_3$)

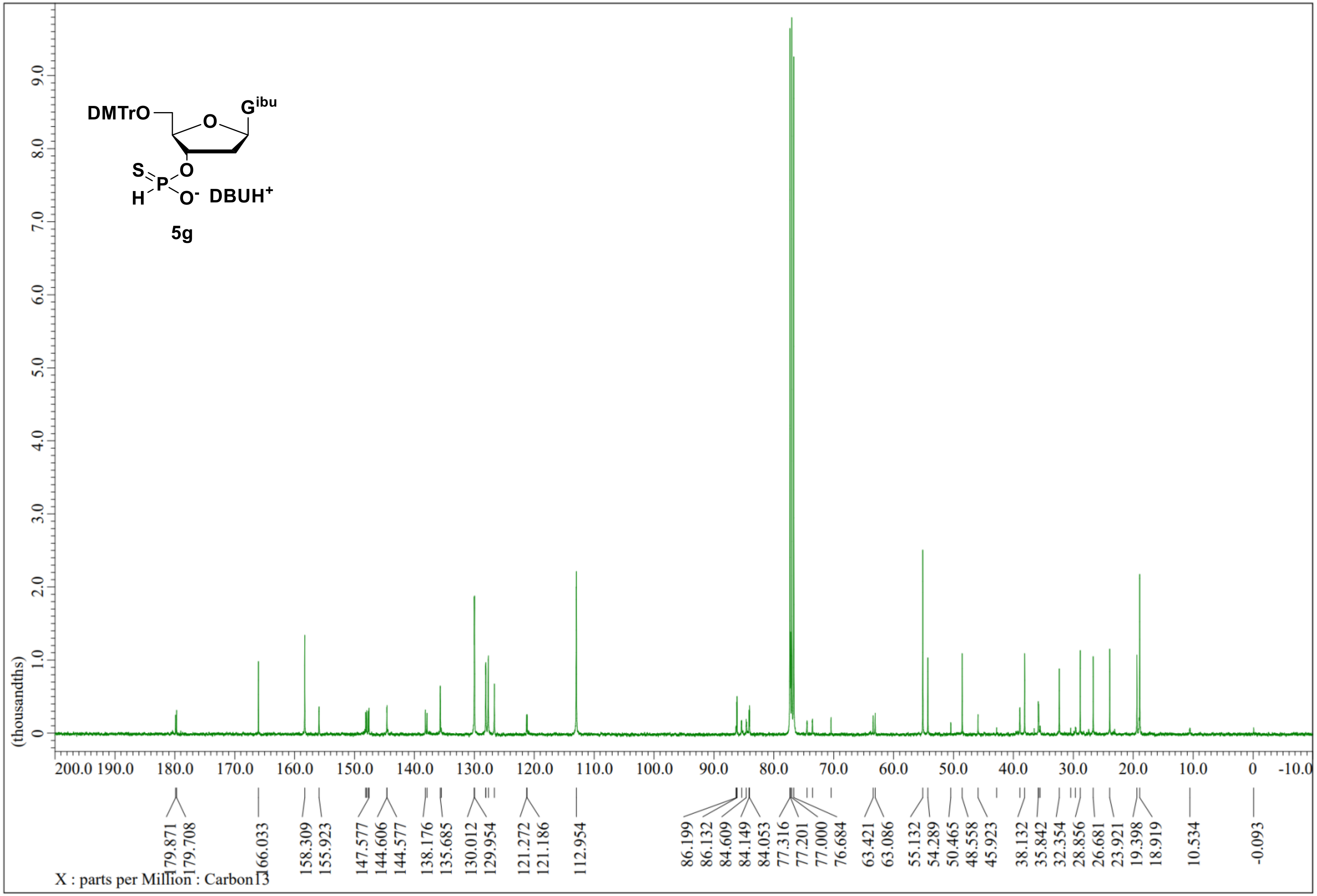

$^{31}P\{^1H\}$ NMR (162 MHz, $CDCl_3$)

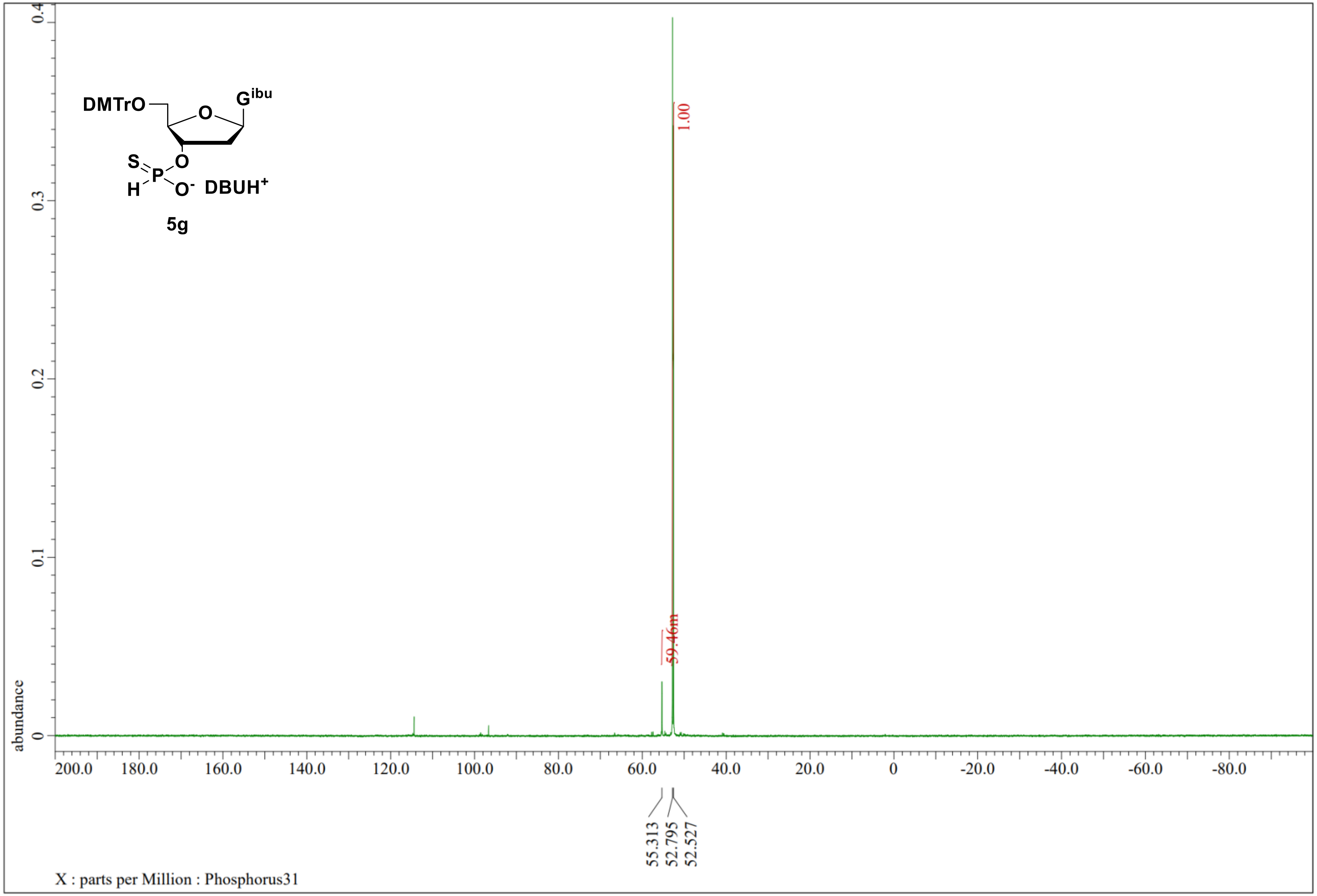

^{1}H-NMR (400 MHz, $CDCl_3$)

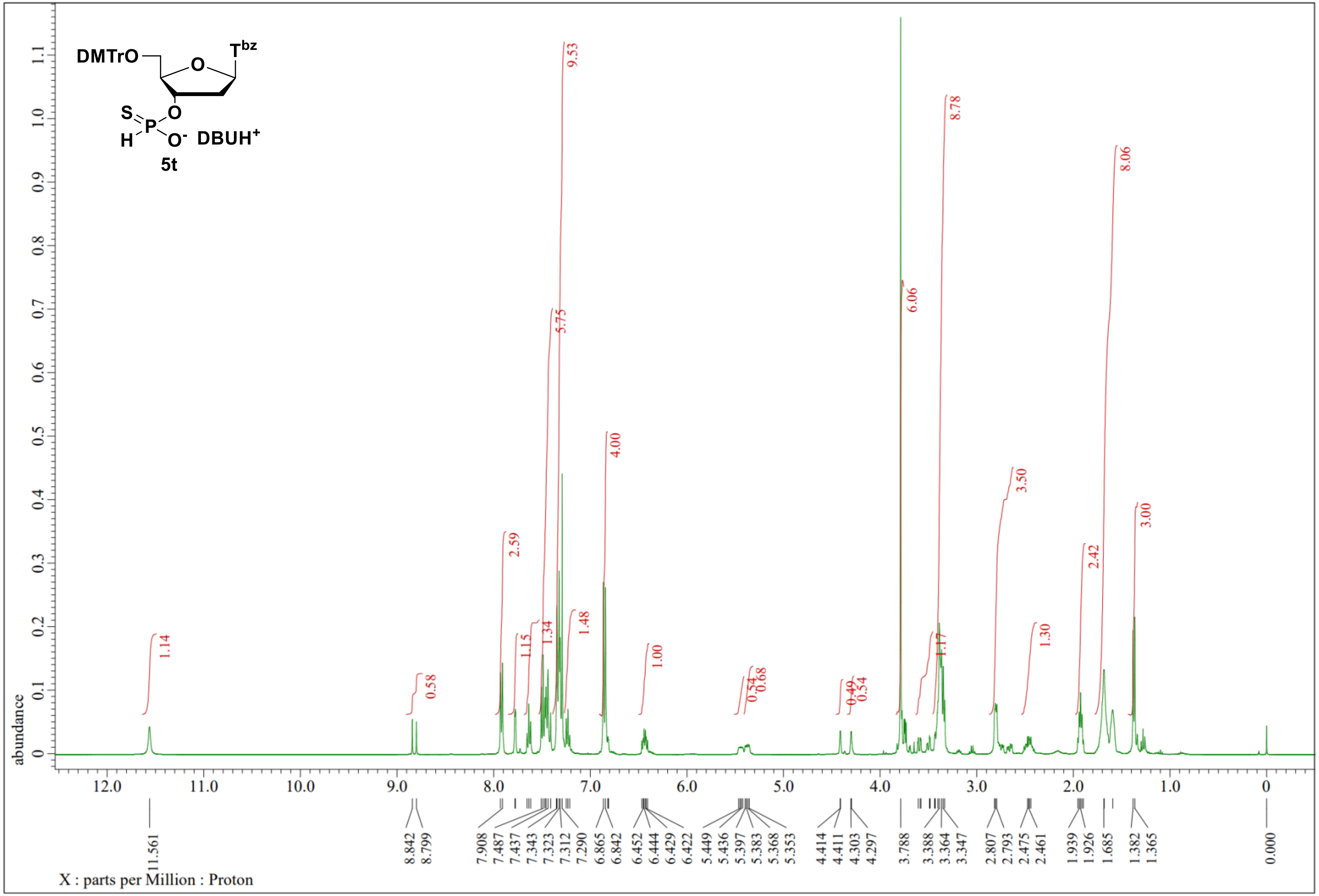

$^{13}C\{H\}$ NMR (100 MHz, $CDCl_3$)

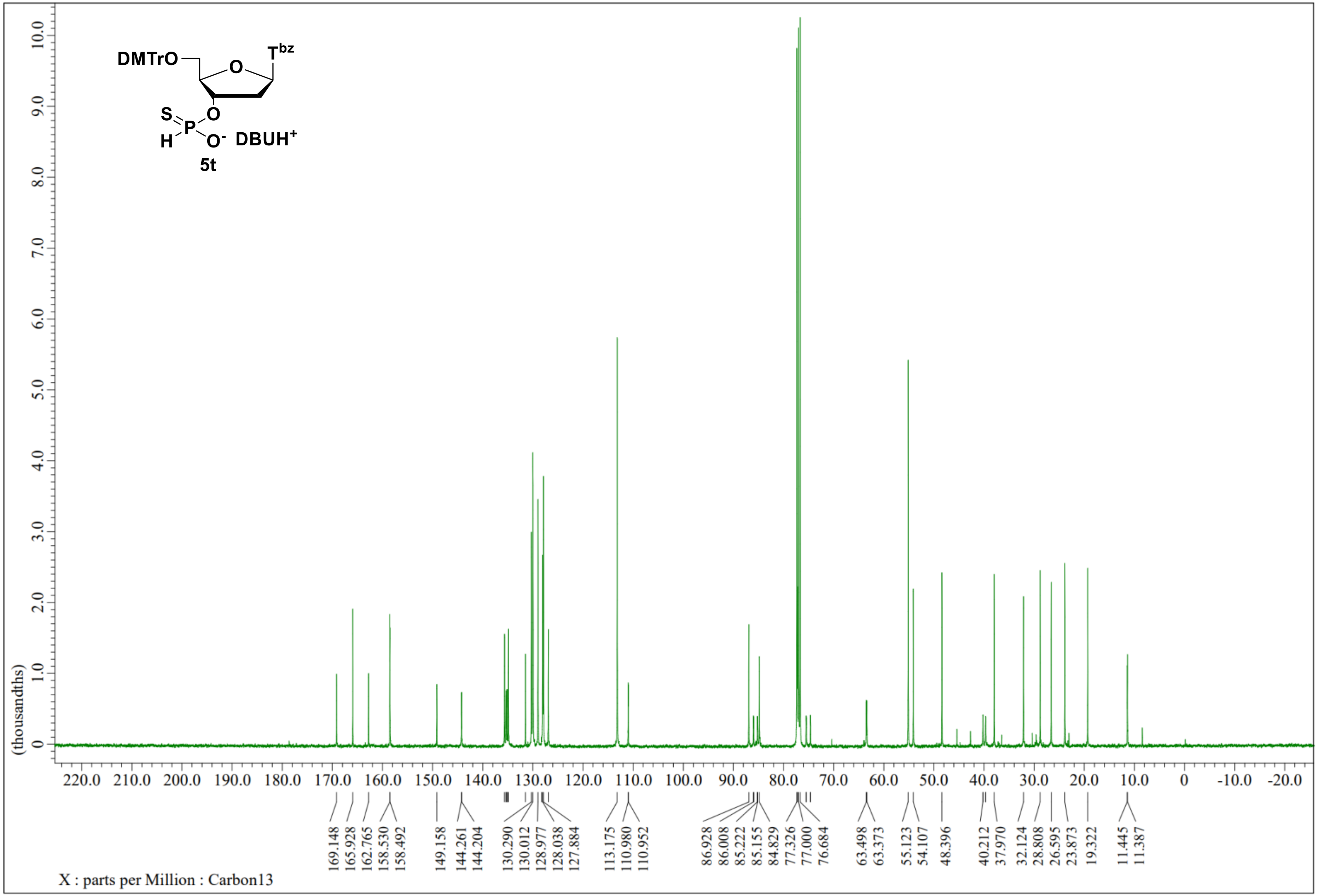

$^{31}P\{^{1}H\}$ NMR (162 MHz, $CDCl_3$)

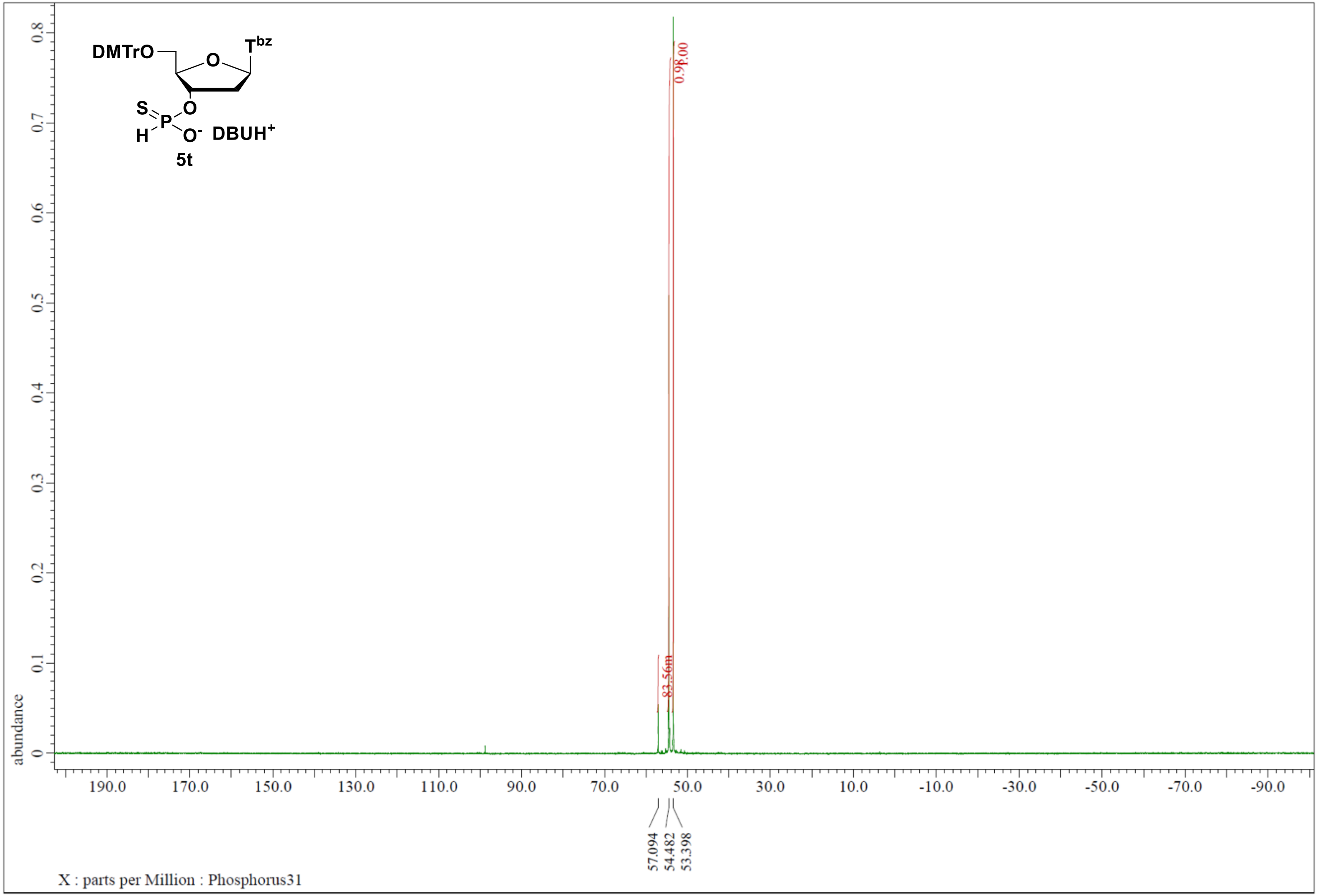

^{1}H-NMR (400 MHz, $CDCl_3$)

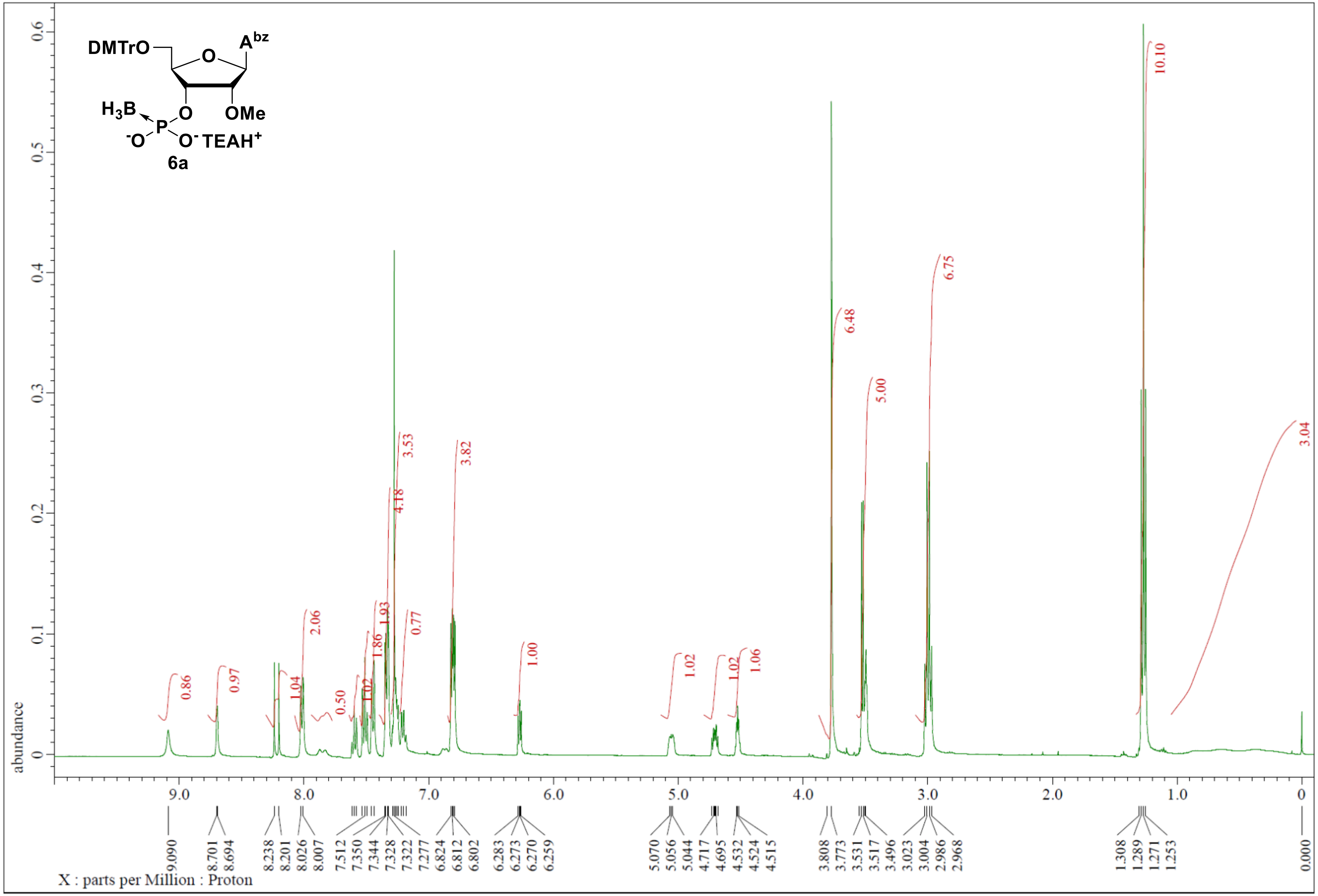

$^{13}C\{H\}$ NMR (100 MHz, $CDCl_3$)

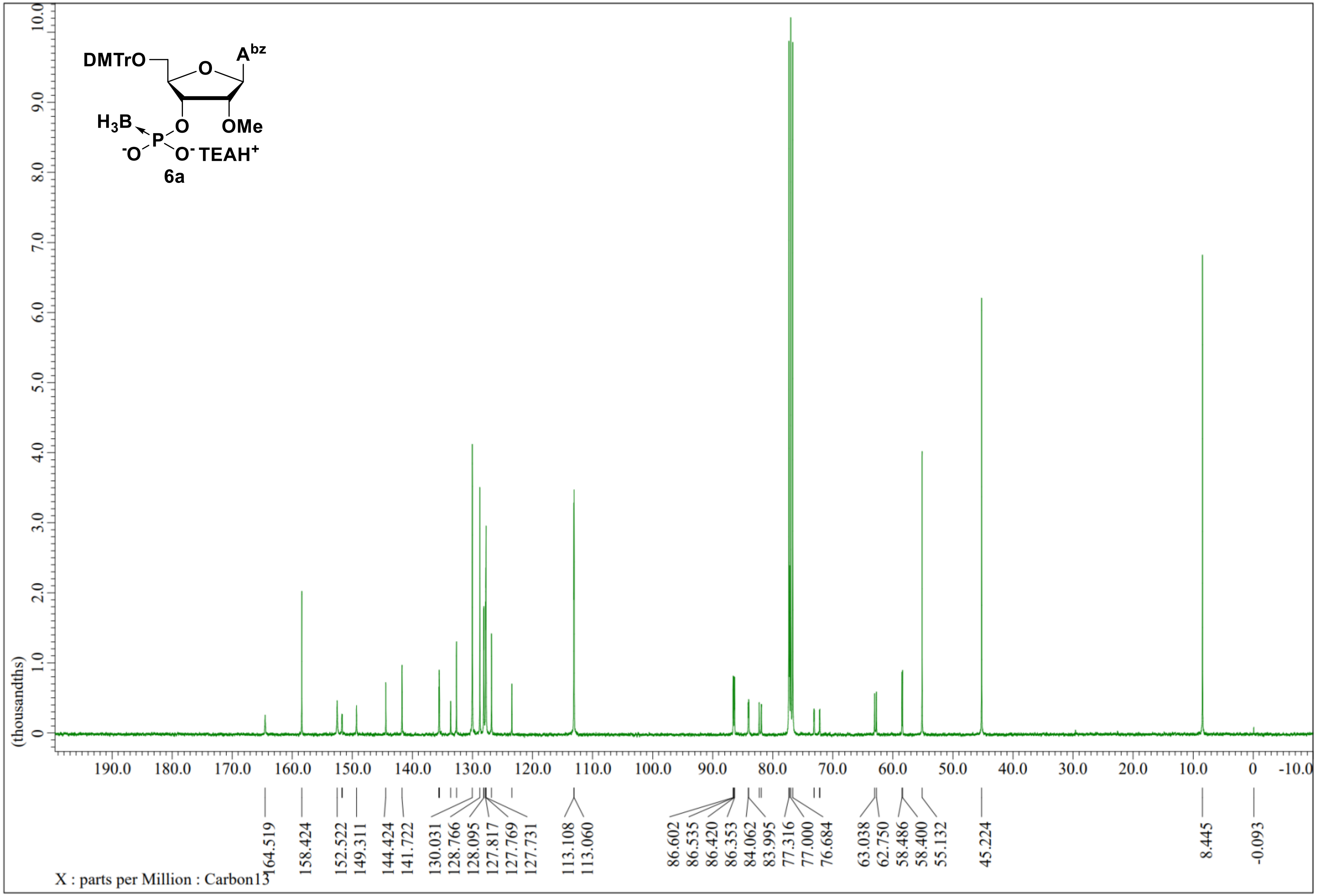

$^{31}P\{^{1}H\}$ NMR (162 MHz, $CDCl_3$)

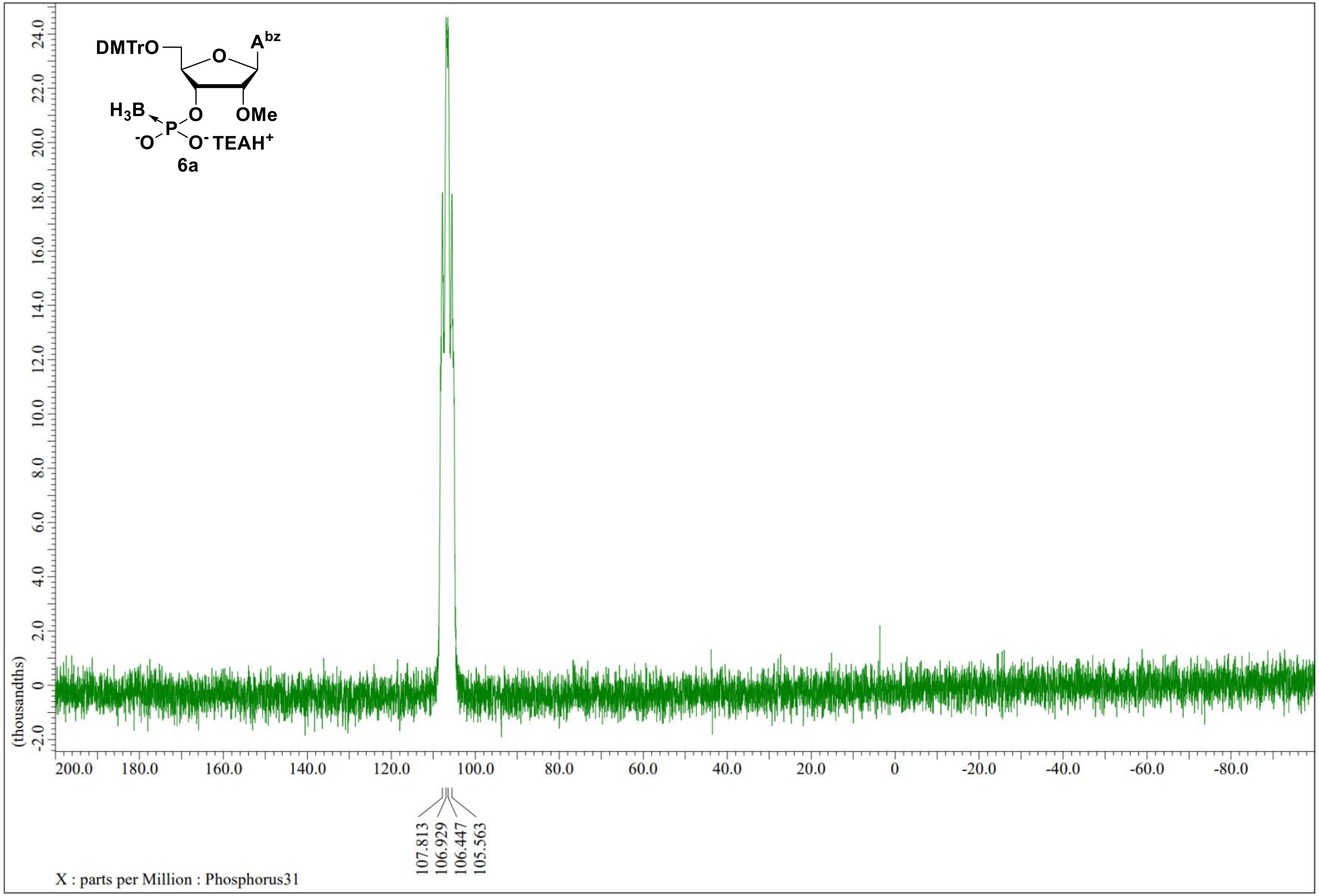

^{1}H-NMR (400 MHz, $CDCl_3$)

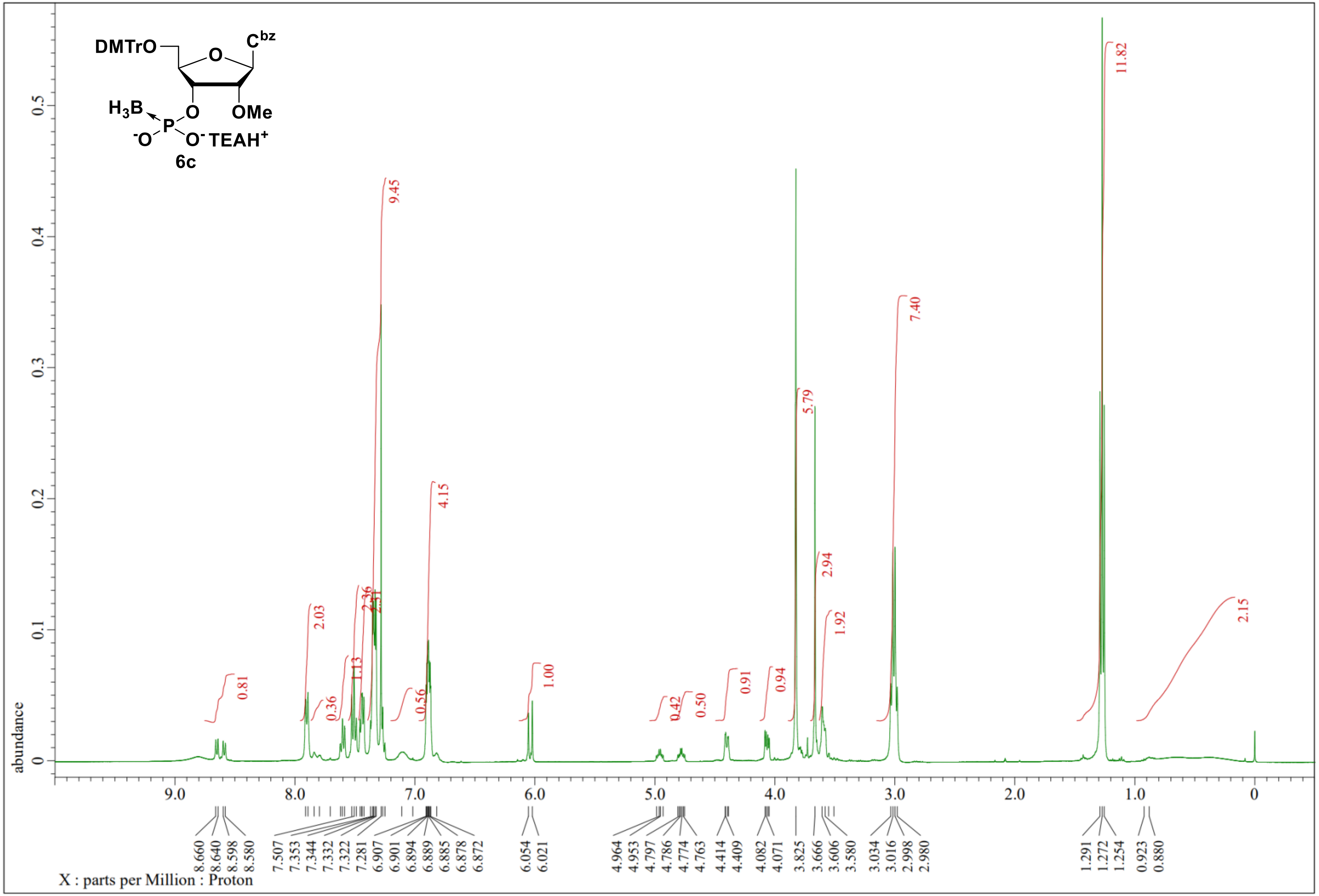

$^{13}C\{H\}$ NMR (100 MHz, $CDCl_3$)

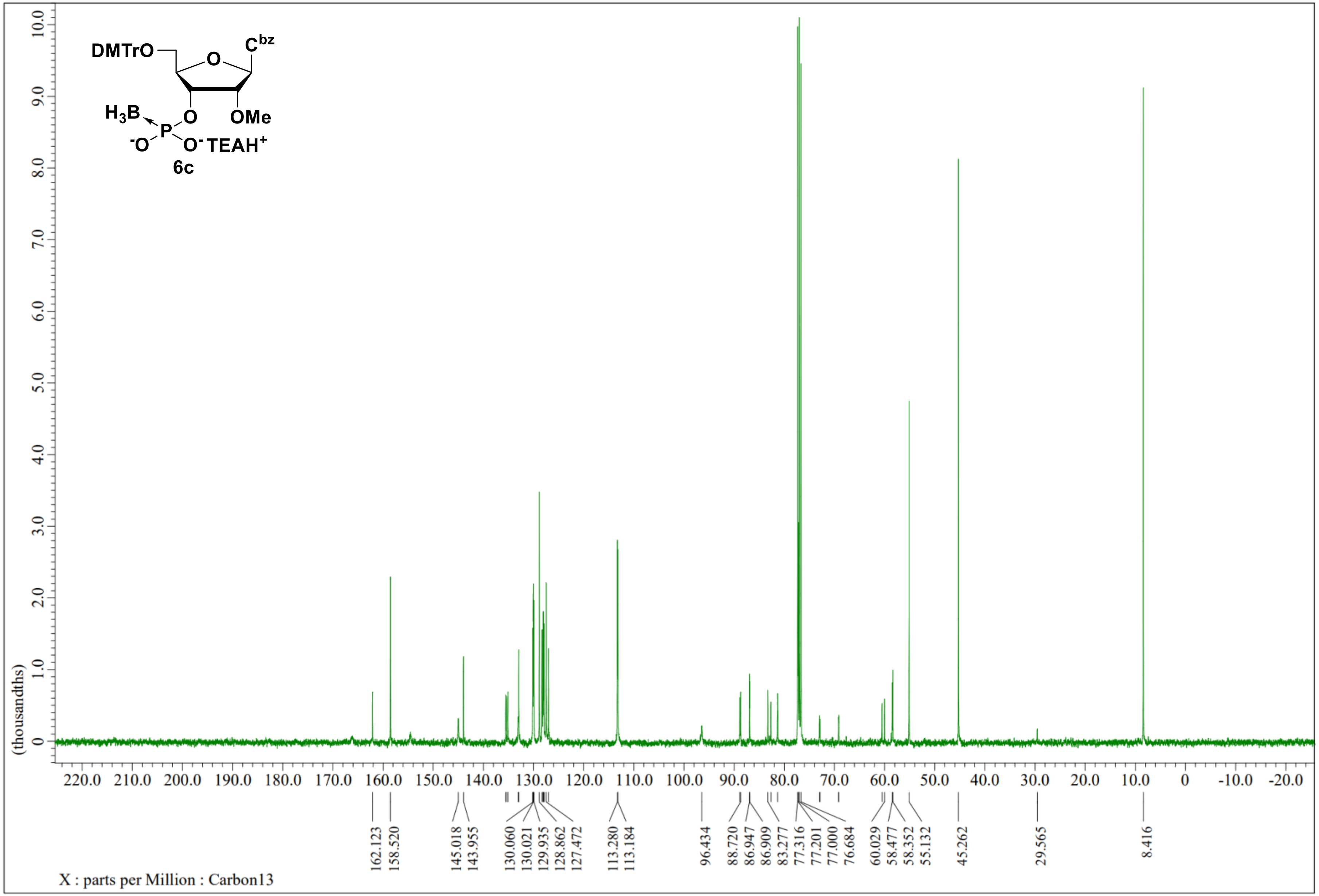

$^{31}P\{^1H\}$ NMR (162 MHz, $CDCl_3$)

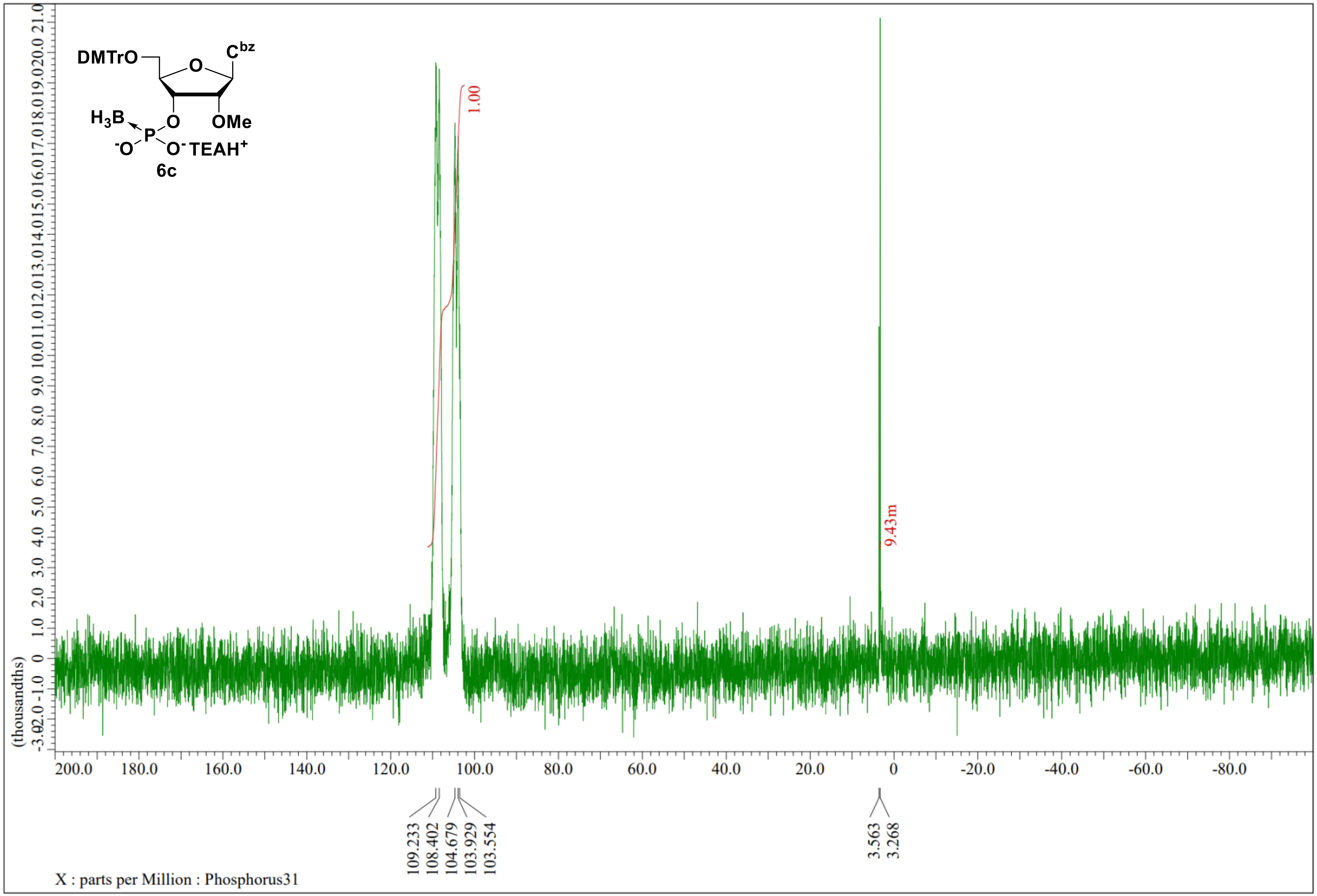

^{1}H-NMR (400 MHz, $CDCl_3$)

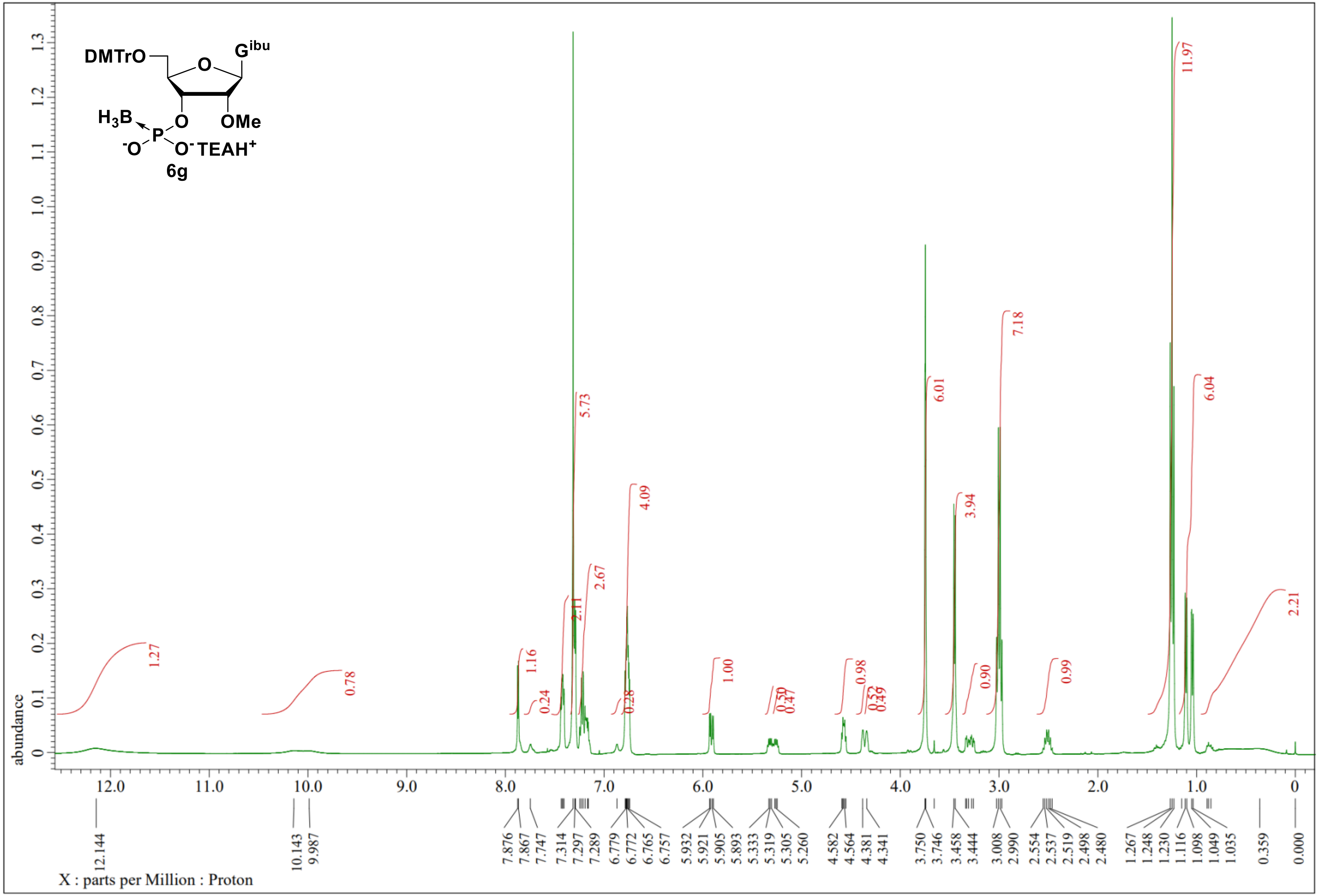

$^{13}C\{H\}$ NMR (100 MHz, $CDCl_3$)

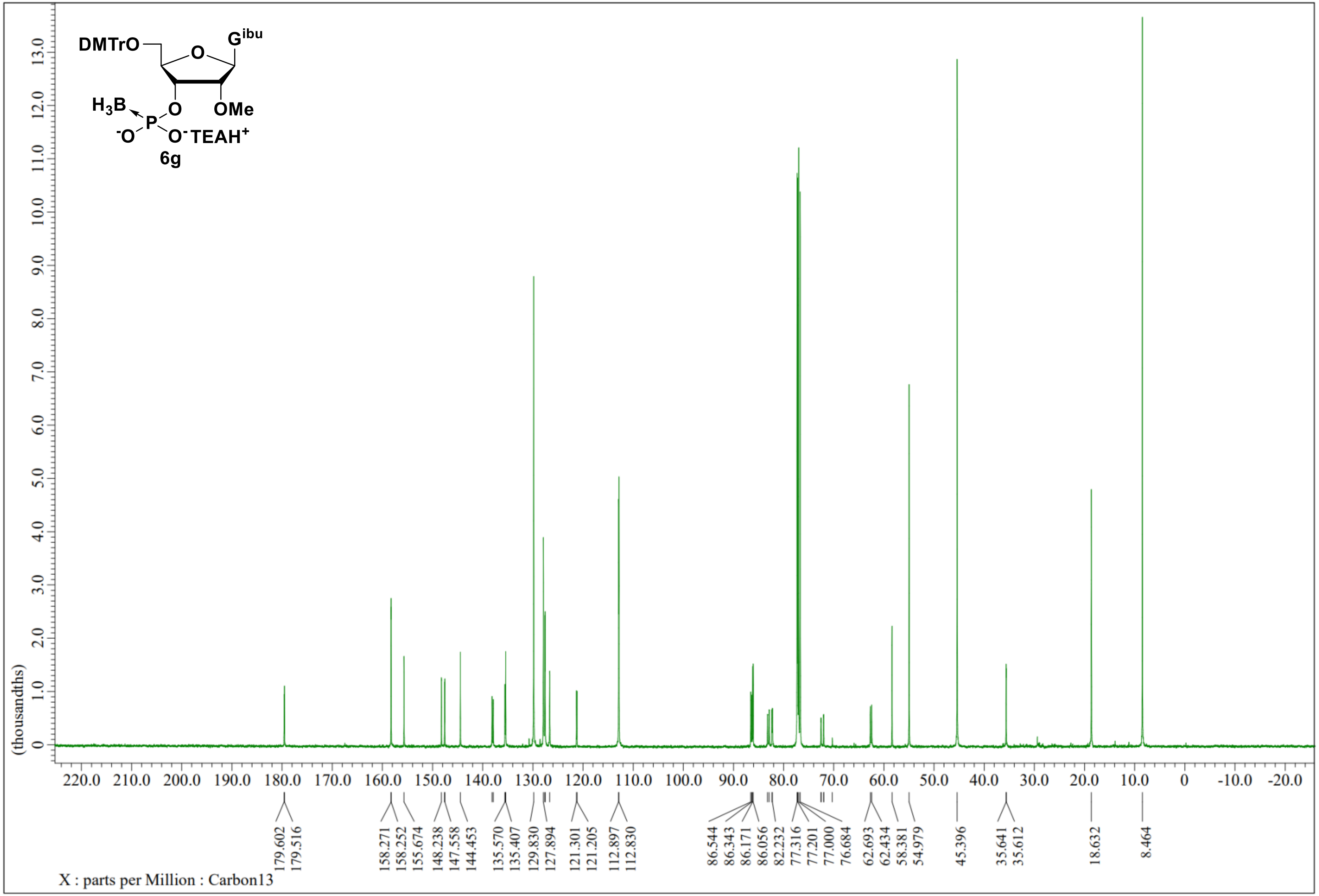

$^{31}P\{^{1}H\}$ NMR (162 MHz, $CDCl_3$)

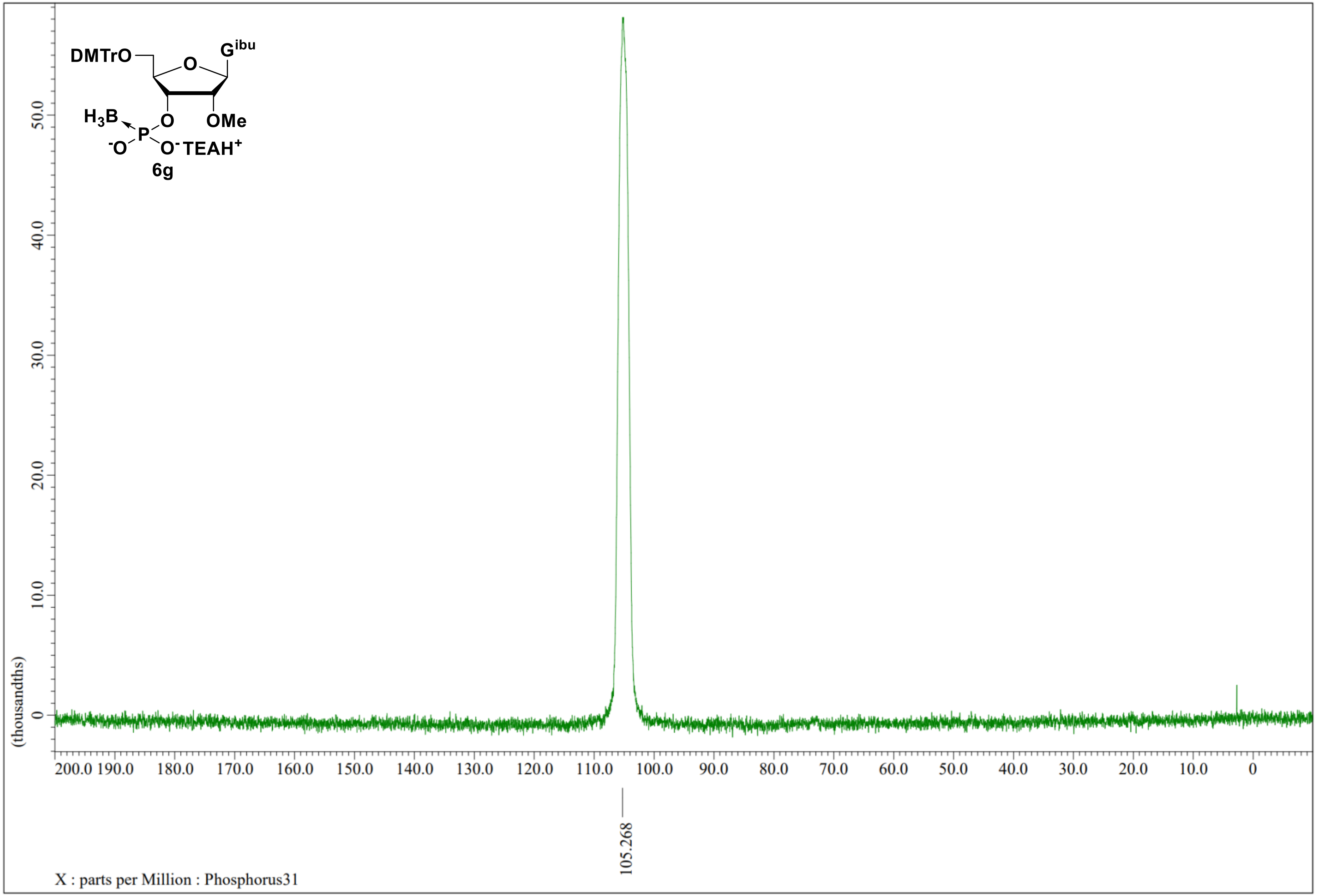

^{1}H-NMR (400 MHz, $CDCl_3$)

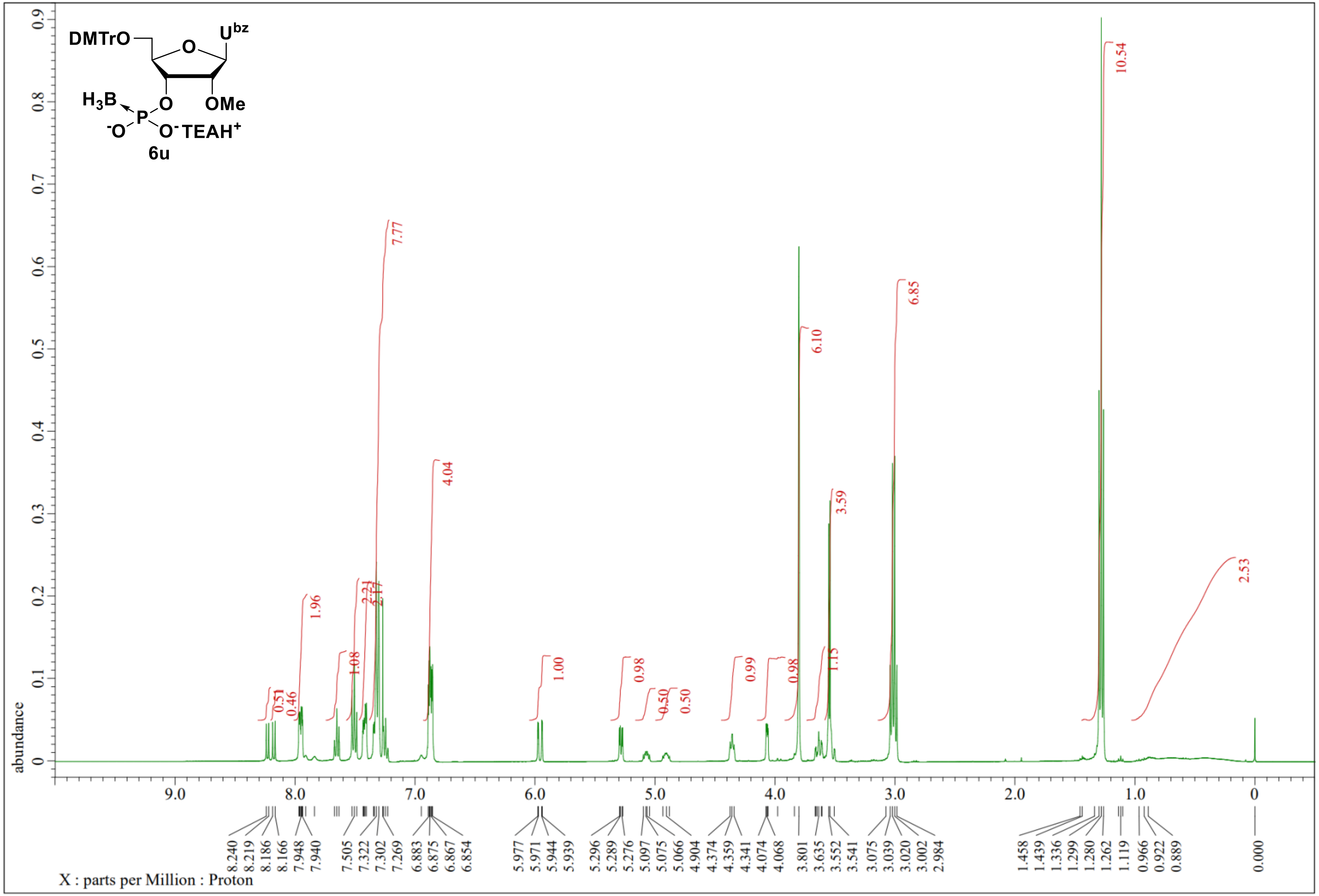

$^{13}C\{H\}$ NMR (100 MHz, $CDCl_3$)

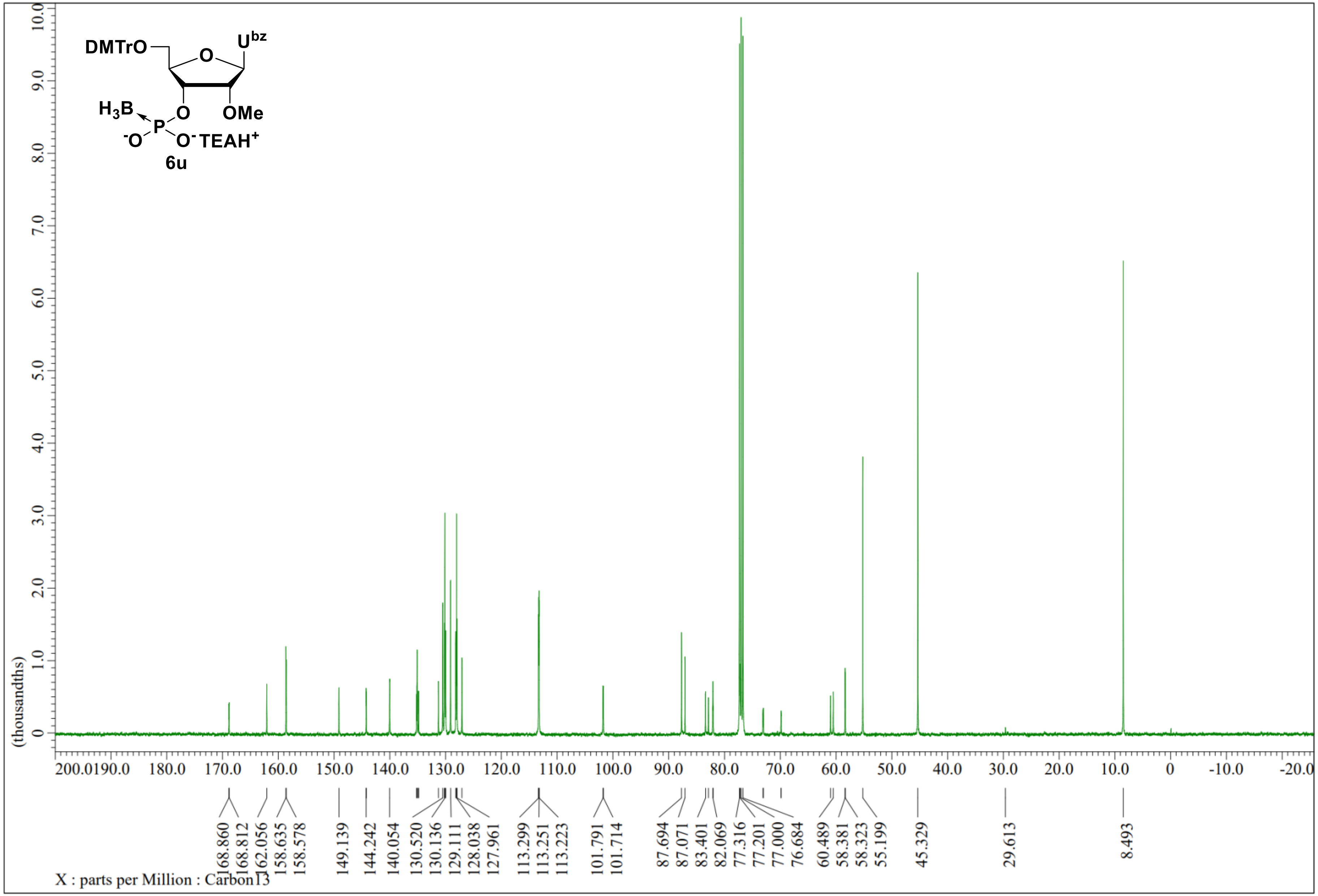

$^{31}P\{^1H\}$ NMR (162 MHz, $CDCl_3$)

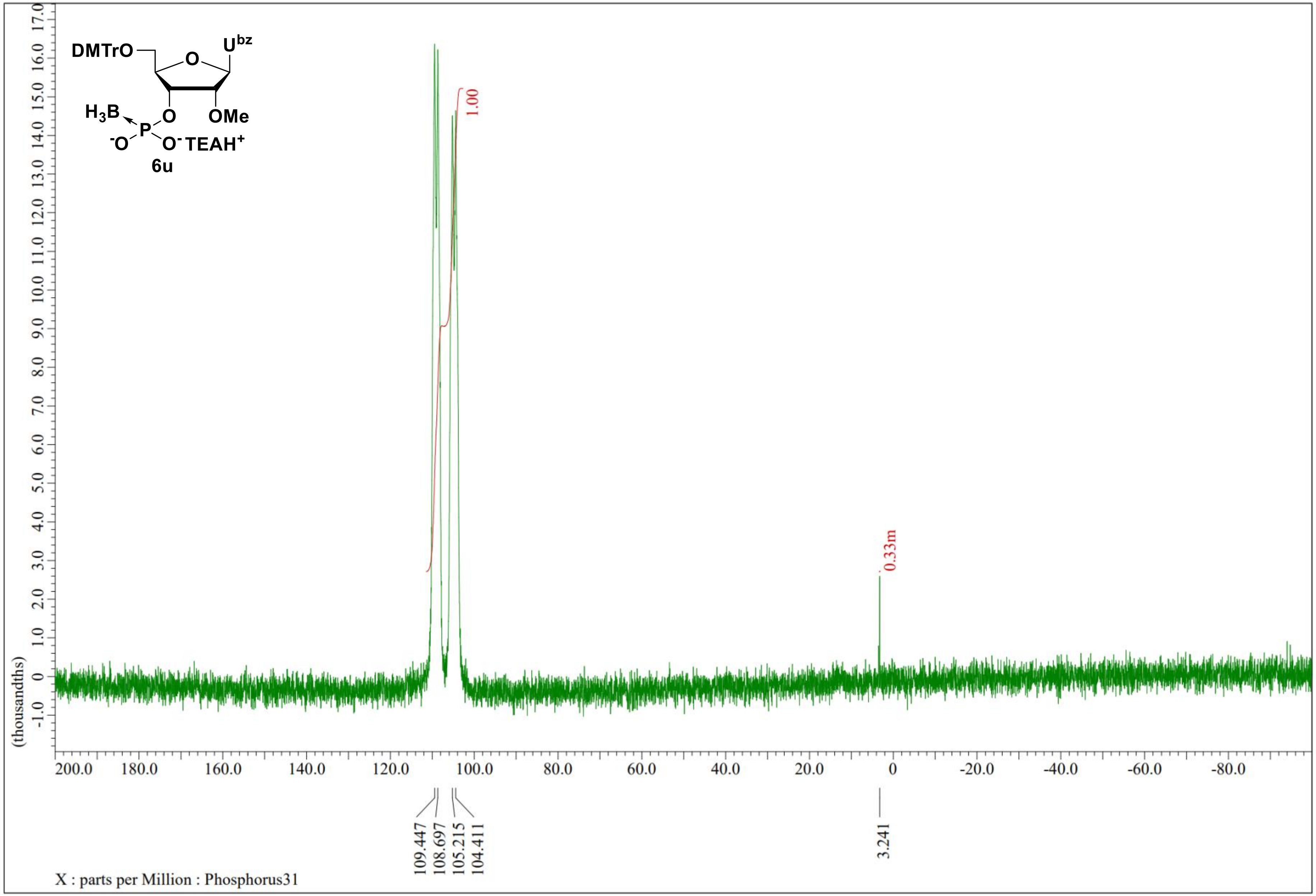

Supplement: Supplementary file 1 — jo1c01812_si_001.pdf [file jo1c01812_si_001.pdf]
